# Evaluation of an in vitro assay to screen for the immunotoxic potential of chemicals to fish

**DOI:** 10.1038/s41598-021-82711-5

**Published:** 2021-02-04

**Authors:** Kristina Rehberger, Beate I. Escher, Andreas Scheidegger, Inge Werner, Helmut Segner

**Affiliations:** 1 Centre for Fish and Wildlife Health, Vetsuisse Faculty, University of Bern, Bern, Switzerland; 2 UFZ-Helmholtz Centre for Environmental Research, Leipzig, Germany; 3 Centre for Applied Geoscience, Eberhard Karls University Tübingen, Tübingen, Germany; 4 Eawag: Swiss Federal Institute of Aquatic Science and Technology, Dübendorf, Switzerland; 5 Swiss Centre for Applied Ecotoxicology, Eawag, Dübendorf, Switzerland

**Keywords:** Environmental sciences, Limnology, Natural hazards

## Abstract

A wide variety of environmental contaminants has been shown to disrupt immune functions of fish and may compromise their defense capability against pathogens. Immunotoxic effects, however, are rarely considered in ecotoxicological testing strategies. The aim of this study was to systematically evaluate the suitability of an in vitro immuno-assay using selected fish immune parameters to screen for chemicals with known immunotoxic potential and to differentiate them from non-immunotoxicants. Non-stimulated and lipopolysaccharide-stimulated head kidney leukocytes of rainbow trout (*Oncorhynchus mykiss*) were exposed for 3 h or 19 h to chemicals with different modes of action. As immune parameters, phagocytosis activity, oxidative burst activity and cytokine transcription (*IL-1β*,* TNFα*,* IL-10*) were examined, accompanied by in silico modelling. The immunotoxicants dexamethasone, benzo(a)pyrene, ethinylestradiol and bisphenol A significantly altered the immune parameters at non-cytotoxic concentrations whereas diclofenac had only weak effects. However, the two baseline chemicals with no known immunotoxic potential, butanol and ethylene glycol, caused significant effects, too. From our results it appears that the in vitro fish leukocyte assay as performed in the present study has only a limited capacity for discriminating between immunotoxicants and non-immunotoxicants.

## Introduction

A wide variety of environmental contaminants has been shown to disrupt immune functions of wildlife species. Such immunotoxic activities bear ecological relevance since efficient immunocompetence is a critical fitness determinant enabling the organism to survive, to minimize the fitness costs of infection and to maintain good growth and reproduction^1–5^. For teleost fish populations living in polluted environments, numerous field observations indicate alterations of immune parameters and/or increased disease incidences^6–13^. Environmental contaminants that are known to disrupt the immune system of fish include dioxin-like chemicals or polycyclic aromatic hydrocarbons^14–21^, endocrine-disrupting compounds^22–29^ and pharmaceuticals like the pain killer, diclofenac (DCF) or the anti-inflammatory drug, dexamethasone (Dex)^30–33^.

Valid diagnostic and testing methods are crucial for assessing the hazard and risk arising from immunotoxic chemicals. In human toxicology, regulatory guidelines containing standardized and validated methods for the assessment of immunotoxic activities of chemicals have evolved from the 1980s (cf.^34–37^). In line with the recent paradigm shift in toxicology (Toxicology in the twenty-first century^38^), increasing attention is given to the use of in vitro assays^39–42^. Although they cannot provide a definitive assessment of adverse immune effects, they can screen potential immunotoxic activities and prioritize positive compounds for further testing (e.g.^40,41,43,44^).

We recently reviewed the literature for tests and endpoints used in immunotoxicity studies with fish^45^. The most frequently analyzed immune parameters were (I) phagocytosis activity (II) respiratory burst activity and (III) immune genes, mainly immune mediators like cytokines at mRNA level. Phagocytosis and oxidative burst are of physiological importance since they are key mechanisms of the innate immune system, which is of particular relevance in fish and, additionally, is well conserved across species. Randelli et al.^46^ mentioned phagocytosis and respiratory burst activity as suitable marker for fish innate immunity and Fournier et al.^47^ highlighted the phagocytosis assay as a suitable biomarker for immunotoxic effects in wildlife populations. The most frequently measured immune genes in fish immunotoxicity studies were cytokines, i.e. interleukins (IL) and tumor necrosis factor (TNF)^45^. Cytokines are important signaling proteins orchestrating the immune response^48^. Among the pro-inflammatory cytokines, IL-1 was evaluated most frequently in fish immunotoxicity studies; among the anti-inflammatory cytokines, it was IL-10^45^. All these parameters used in fish immunotoxicity studies—phagocytosis activity, oxidative burst activity, transcription of cytokines—can principally be assessed in in vitro assays using isolated fish leukocytes. Such an assay therefore could be a prime candidate for screening of chemicals with immunotoxic potential in fish. However, a systematic evaluation of the ability of an in vitro fish leukocyte assay to detect immunotoxicants and to discriminate them from non-immunotoxicants has not been undertaken to date.

The aim of the present study was to examine if an in vitro fish leukocyte assay is a suitable tool to screen for immunotoxic potentials of environmental chemicals to fish. We hypothesised that at least one immune parameter measured in this assay—phagocytosis, respiratory burst activity or cytokine transcripts—would be responsive to immunotoxicants while none of the immune parameters would respond to non-immunotoxicants (Table 1). We used leukocytes isolated from the head kidney (HK, a major fish immune organ) of female, non-exposed rainbow trout (*Oncorhynchus mykiss*). Since the immune system reactivity can differ between the resting and activated state^49^ evaluations were carried out concurrently with non-stimulated leukocytes and with leukocytes stimulated by bacterial lipopolysaccharide (LPS). Chemical exposure was conducted in vitro for 3 h or 19 h. The tested immunotoxic chemicals included compounds that interfere with the immune system through different modes of action. All tests were performed at sub-cytotoxic concentrations in order to avoid false positive responses of the immunological parameters caused by adverse effects of high concentrations of test chemicals on cell viability (cf.^50^). In vitro effect concentrations were compared with in silico predictions for baseline cytotoxicity^51^ to identify how much more potent the specific effects were in comparison to baseline cytotoxicity^52^. Importantly, the purpose using this in vitro assay was neither to predict adverse outcomes nor toxic effect concentrations in whole fish but to screen for chemicals with immunotoxic potential and to discriminate them from non-immunotoxic chemicals.

**Table 1:** Nominal concentrations of chemicals for assessing the immune parameters, and their molecular initiating event (MIE)/mode of action (MoA).

Test chemical	Test concentrations (nominal)	MIE/MoA	Reference supporting the MIE/MoA in fish	Reported immunotoxic activity in fish	Reference supporting the immunotoxic action in fish
Dexamethasone (Dex)	0, 1, 5, 10 µM solvent: MeOH	GR	^124,125^	Yes	^31–33^
Diclofenac (DCF)	0, 1, 5, 10, 50 µM	COX	^107^	Yes	^30,32^
Benzo(a)pyrene (BaP)	0, 0.1, 0.5, 1 µM solvent: AcN	AhR and via metabolites	^16–18^	Yes	^20,71,126,127^
Bisphenol A (BPA)	0, 0.5, 1, 2.5, 5 µM	ER, AhR, PPAR, NF-κB	^95,96^	Yes	^97,103,104^
17α-Ethinylestradiol (EE2)	0, 0.25, 2.5, 5 µM solvent: EtOH	ER	^25–27^	Yes	^22,24,91,128^
Ethylene glycol (EG)	0, 10, 25, 50, 75 mM	polar narcotic; Verhaar class 2	^129^	No	–
1-Butanol (But)	0, 2.5, 5, 7.5, 10 mM	narcotic/baseline; Verhaar class 1	^130–132^	No	–
1,2,4-Trichlorobenzene (TCB) *	0, 2.5, 7.5, 25 µM solvent: EtOH	narcotic/baseline; Verhaar class 1	^130–135^	No	–

*MeOH* methanol, *AcN* acetonitrile, *EtOH* ethanol, *GR* glucocorticoid receptor, *COX* cyclooxygenase, *AhR* aryl hydrocarbon receptor, *ER* estrogen receptor, *PPAR* peroxisome proliferator-activated receptor, *NF-κB* nuclear factor kappa-light-chain-enhancer of activated B cells. *TCB: the immuno-assay was conducted but results were excluded retrospectively since QASR modelling revealed a loss to air of about 90%.

## Methods

### Test chemicals and reagents

Test chemicals are listed in Table 1 including references providing information on their molecular initiating events (MIE)/mode of action (MoA) as well as references describing the immunotoxic activity of these compounds in fish. Dexamethasone (Dex) was used as reference compound since it is a well-established immunosuppressive agent for fish. Other immunotoxicants were diclofenac (DCF), benzo(a)pyrene (BaP), bisphenol A (BPA) and 17α-ethinylestradiol (EE2). The selection criteria for the three non-immunotoxic chemicals—ethylene glycol (EG), 1-butanol (But), 1,2,4-trichlorobenzene (TCB)—are provided in Table 1, too. For those chemicals we did not find any indication that they possess an immunotoxic activity (key words for the literature search in the Supplementary Material S1a) and they are categorized as baseline/narcotic compounds. For trichlorobenzene, the immuno-assay was conducted but the results were excluded retrospectively, since the QASR modelling revealed a loss to air of about 90%. Detailed information on the chemicals and reagents used is provided in Supplementary Material S1b. For the chemical exposure, stock solutions were prepared as followed (chemicals in alphabetic order): benzo(a)pyrene: 1 mM in acetonitrile, stored in aliquots at − 20 °C; bisphenol A: 500 µM in medium, stored in aliquots at − 20 °C; butanol: no stock solution prepared; dexamethasone: 25.45 mM in methanol; diclofenac: 5 mM in water, stored in aliquots at − 20 °C; ethinylestradiol: 8 mM in ethanol; ethylene glycol: no stock solution prepared; trichlorobenzene: 27.56 mM in ethanol. The required test concentrations were achieved by dilution with medium. If a solvent was required for preparation of the chemical solutions, a solvent-containing (below 0.1%) control was included in the immuno-assay in addition to the solvent-free control. Although presented data was normalized to the solvent control (if a solvent was used), the solvent-free control was still assessed in the viability assay to exclude toxic effects due to the solvent itself (see Supplementary Material S6).

### Terminology

For better reader-friendliness, the phrase “chemicals with immunotoxic potential” is shortened to “immunotoxicants”, the phrase “chemicals with no reported immunotoxic potential” to “non-immunotoxicants” through the paper. Besides, in this article we use the terms “immunomodulation” and “immunotoxicity” synonymously. Nevertheless, we are aware that “immunomodulation” would be the more accurate term for chemical-induced alterations of fish immune parameters since such alteration does not necessarily imply an adverse, toxic effect on immunocompetence of the organisms. The term “cytotoxicity” is used to describe an acutely lethal effect of the test chemical on cell viability.

### Fish husbandry and leukocyte isolation

Head kidney (HK) leukocytes from all-female, not yet reproductively active rainbow trout (*Oncorhynchus mykiss*) of 777 ± 229 g body weight and 38 ± 4 cm total length were used in this study. Fish were obtained from “Pisciculture de Vionnaz Hess SA” (Switzerland) and maintained at the Centre for Fish and Wildlife Health, University of Bern, Switzerland. The experiment was approved by the ethical committee of the Canton of Bern under permit number BE 44/15. The study was performed in accordance with relevant guidelines and regulations. The crucial assay parameters such as cell isolation, cell culture, cell density, incubation times and conditions for measuring phagocytosis, respiratory burst and cytokine transcription, including the steps for qPCR analysis and lipopolysaccharide (LPS) stimulation were optimized in the context of the applied study conditions. The protocol for HK leukocyte isolation was modified from Garduño and Kay^53^ and Braun-Nesje et al.^54^: trout were euthanized with an overdose of buffered MS222 (tricaine mesylate) followed by a gill cut. The HK was removed, weighed and mechanically disaggregated using nets of 105 µm mesh size. The disaggregation was carried out under sterile conditions in an organ-weight dependent volume of medium (RPMI including 50 mM HEPES and 7 mM NaHCO3, pH 7.4) supplemented with 0.5% fetal bovine serum (FBS, heat inactivated, charcoal-stripped, sterile) and 10 U/ml heparin. The resulting suspension was centrifuged (450 rcf, 35 min, 4 °C) over a discontinuous Percoll gradient with 1.04 and 1.075 density. Leukocytes were collected from the interphase, washed twice (250 rcf, 10 min, 4 °C) with 10 U/ml heparin supplemented medium and once with non-supplemented medium. Cell number was adjusted to 1 × 10^6^ viable cells/ml medium using trypan blue staining and a hemocytometer. Cells were then plated in 96-well plates (outer rows filled with cell-free medium) using medium supplemented with 0.5% FBS. The cell number per well depended on the effect parameter, see below for details. Cells were incubated overnight in a humid and aerated cell incubator at 17 °C and further processed the next day.

### LPS stimulation and test chemical exposure

The conditions for stimulation were optimized in a series of prior tests using three different sources of LPS (O111:B4, O55:B5 and O26:B6) and beta-glucan and combinations thereof at different concentrations and exposure durations as well as prior and parallel to the chemical exposure (data not shown). Based on those results LPS from *Escherichia coli* 0111:B4 (final concentration 10 µg/ml medium^55^) was added to half of the wells after the overnight incubation and incubated for 3 h to activate the immune cells. Subsequently, medium was removed from both non-stimulated and LPS-stimulated cells. Leukocytes were then exposed to a range of concentrations of the test chemicals in fresh, LPS- and FBS-free medium for a short-term (3 h) or long-term (19 h) period. Each test chemical was measured in five to seven independent biological replicates (= fish). The numbers of biological replicates (fish n-numbers) utilized for statistical analysis are listed in Supplementary Material S4 for each test chemical and immune parameter. LPS treatment served as positive and quality control (see “Data analysis”). Within each individual biological replicate, leukocyte exposure was performed in three technical replicates (= wells) for each treatment. The values of the technical replicates were averaged for statistical analysis. The amount of HK leukocyte obtained from one fish was quite limited, but we were still able to measure all parameters in each exposure scenario (short- and long-term exposure, without and with LPS stimulation) for one test chemical in parallel. Separate 96-well plates were used for long- and short-term exposure as well as for every parameter assessed.

### Cytotoxicity assay to determine cell viability

To avoid interference of cytotoxicity with specific immunotoxic effects we determined the cytotoxic concentration range for each chemical prior to the main experiments. To this end, the cells were exposed for 3 and 19 h to a concentration series of each test chemical, with and without prior LPS stimulation. During the main experiment, cell viability was monitored in parallel to the immune parameters. Cell viability was measured using Calcein-AM (calcein acetoxymethyl ester, final concentration 1 µM, 30 min incubation, 1.5 × 10^5^ cells/well, modified from Lilius et al.^56^). The value was normalized to the total number of cells as determined by means of DAPl nuclei staining (4′,6-diamidino-2-phenylindole dihydrochloride, final 5 µg/ml, 2 h incubation). Dye intensity was scanned over the entire well-bottom in a 2300 EnSpire Multilabel Plate Reader (PerkinElmer) at λ 495 nm/517 nm for Calcein-AM and λ 358 nm/461 nm for DAPI. Data was normalized against the blanks (cells without dye). Test chemical concentrations that resulted in more than 80% cell viability (equivalent to < 20% cytotoxicity, EC20_Cytotox_) in the range finding were used to evaluate the immune parameters and to run the in silico modelling in the main experiment.

### Phagocytosis activity

The phagocytosis activity was evaluated with a method adapted from Harford et al.^57^. Leukocytes (2.5 × 10^5^ cells/well) were simultaneously exposed to the test chemical and 1 µm yellow-green-fluorescently labeled latex beads (cell:bead ratio of 1:12). Accordingly, cells were allowed to ingest beads for 3 h or 19 h, depending on the exposure conditions. Afterwards, cells were placed on ice to prevent further phagocytosis. Beads were then added to three wells of non-exposed, control leucocytes which represented the blanks. The blanks were used to correct for potential uptake or attachment of beads to the surface of the cells that may have happened during the short time between placing the cells on ice and the flow cytometry measurement. Next, the supernatant of each well was transferred into an individual 1.5 ml tube and cells were detached from culture plates using trypsin. The resulting cell suspension was added to the supernatant and centrifuged at 200 rcf and 4 °C for 8 min. The supernatant was discharged by pipetting and cells were re-suspended in fresh chemical- and bead-free medium. Subsequently, 10,000 cells were measured in a flow cytometer. Experiments with dexamethasone, ethinylestradiol and trichlorobenzene were analyzed on a BD FACSCanton II flow cytometer, the other experiments on a BD LSR II which broke during the study, but results were similar for both devices. This was confirmed by repeating a bisphenol A exposure and analyzing it on the BD FACSCanton II.

The percentage of phagocytosis-active cells was determined as follows using the software program FlowJo v10: cells of interest were selected (= gated) in a forward scatter (FSC) *vs*. side scatter (SSC) plot. Based on their forward and sideward properties, gates were placed around myeloid-like and lymphocyte-like cells as well as a third gate for both cell types together (“all cells”). Within the selection, propidium iodide negative (final concentration 3 µM), single cells (FSC-A vs*.* FSC-H) that additionally stained for FITC (fluorescein isothiocyanate, detection of the beads) were considered to represent the number of phagocytosis-active cells which ingested at least one bead. Data obtained was normalized against the blanks. The data presented in this study is based on the phagocytic activity of the myeloid-like cell fraction such as granulocytes and macrophages. Initially, the three gates of the FSC-SSC-plot were analyzed individually and data for myeloid-like cells yielded the clearest pattern although results were similar for all approaches. This is in accordance with results of other studies: cells that sense pathogens through receptors such as granulocytes (myeloid-like) are particularly active ingesting foreign particles^58^ even though fish lymphocytes conduct phagocytosis, too^59–61^. Initially, data for myeloid-like cells were additionally analyzed according to the number of ingested beads: (a) one, (b) two or (c) three and more beads. Results obtained were similar to analysis results on phagocytosis independent of the number of ingested beads. Thus, the latter is presented in this article.

### Respiratory burst activity

During a respiratory/oxidative burst, leukocytes generate reactive oxygen species (ROS) to attack phagocytosed pathogens. The impact of chemical exposure on the respiratory burst activity of leukocytes was assessed using a nitro blue tetrazolium (NBT) assay adapted from Rymuszka and Adaszek^62^: after chemical exposure the 96-well plates were centrifuged (300 rcf, 2 min) and leukocytes (2 × 10^5^ cells/well) were incubated for 2 h in fresh, chemical-free medium containing 1 mg/ml NBT. Afterwards, plates were centrifuged (300 rcf, 2 min) and medium was replaced by 100% MeOH for 2 min. MeOH was removed after additional centrifugation (300 rcf, 2 min). 2 M KOH and DMSO were added to the dried wells, mixed to dissolve crystals and plates were centrifuged (300 rcf, 2 min). An aliquot (150 µl) of the supernatant was transferred into a clean well and photometrically measured at λ 630 nm. Data was normalised against the blanks (cells without NBT).

### qRT-PCR analysis of cytokine mRNA transcript levels

To assess the effect of chemical exposure on the cytokine mRNA transcript level (*IL-1β, TNFα* and *IL-10*), quantitative real-time PCR (qRT-PCR) analysis was conducted. In a series of prior tests, we evaluated the suitability of different RNA extraction kit, optimized the RNA extraction, assessed the quality of the extracted RNA by using the bioanalyzer methodology and we optimized the cDNA synthesis as well as the qPCR protocol. For the measurements, the 96-well plates (2 × 10^5^ cells/well) were centrifuged (300 rcf, 2 min) after chemical exposure, medium was removed and plates were subsequently stored at – 80 °C. Only cells from the control and the highest chemical concentration were analyzed. RNA was extracted using the ReliaPrep RNA Cell Miniprep System (Promega) with two modifications: (I) RNA was extracted from pooled cells of two technical replicates per treatment by consecutive centrifugation of replicates on the same extraction column. (II) RNA was eluted from the extraction column using two times 7.5 µl nuclease-free water. RNA yield was estimated using the NanoDrop 1000 spectrophotometer (Witec AG). For cDNA synthesis, 100 ng RNA were used and the GoScript Reverse Transcription Mix, Random Primers (Promega) protocol was followed. For qRT-PCR analysis the SYBR-green-type GoTaq qPCR Master Mix (Promega) protocol was applied with the following modifications: hot-start activation 5 min and 45 cycles. Sample composition are listed in Supplementary Material S1c, primer sequences are listed in Table 2 including references which evaluated the rainbow trout primers in the same lab as in which the present study was conducted. All samples were measured in technical duplicates using the 7500 Fast Real-Time PCR System (Applied Biosystems) and the SDS Software version 1.3.1 (Applied Biosystems). NTCs (negative controls, containing no cDNA but water) and melting curves were carried out for each plate to exclude contamination or erroneous amplification. For data analysis the threshold (i.e. signal exceeds background level) was set at Ct 0.2 and the baseline between cycle 3–15. Calculations were made using the 2^−ΔΔCt^ method. We analyzed transcript levels of two reference genes, *18S* and *EF-1α*. Although expression of both reference genes was stable, *EF-1α* expression showed less variability between time points and between treatments and controls, thus data was normalized to *EF-1α*. Mean Ct values for *EF-1α* are displayed for all test chemicals in Supplementary Material S1d. The effects on cytokine expression are expressed as fold change in relation to the control (= 1) of each fish to account for possible variation between fish in the basal expression level.

**Table 2:** Primer sequences and corresponding references.

Gene	Forward primer (5′–3′)	Reverse primer (5′–3′)	References
*18S*	TGCGGCTTAATTTGACTCAACA	CAACTAAGAACGGCCATGCA	^136^
*EF-1α*	TGCCCCTGGACACAGAGATT	CCCACACCACCAGCAACAA	^137^
*IL-10*	CTGCTGCTCCTTCGTAGAGG	CTCGTCATTAGCCTCGTAGTAGTCTC	^138^
*IL-1β*	AGTGCTGTGGAAGAACATATAGTGTTG	CATCAGGACCCAGCACTTG	^138^
*TNFα1*+*2*	AGGGGACAAACTGTGGACTG	GTGCAAACACACCAAAGAAGTT	^138^

### In silico modelling to determine baseline toxicity and degree of specificity of the effect

**Figure 1:**
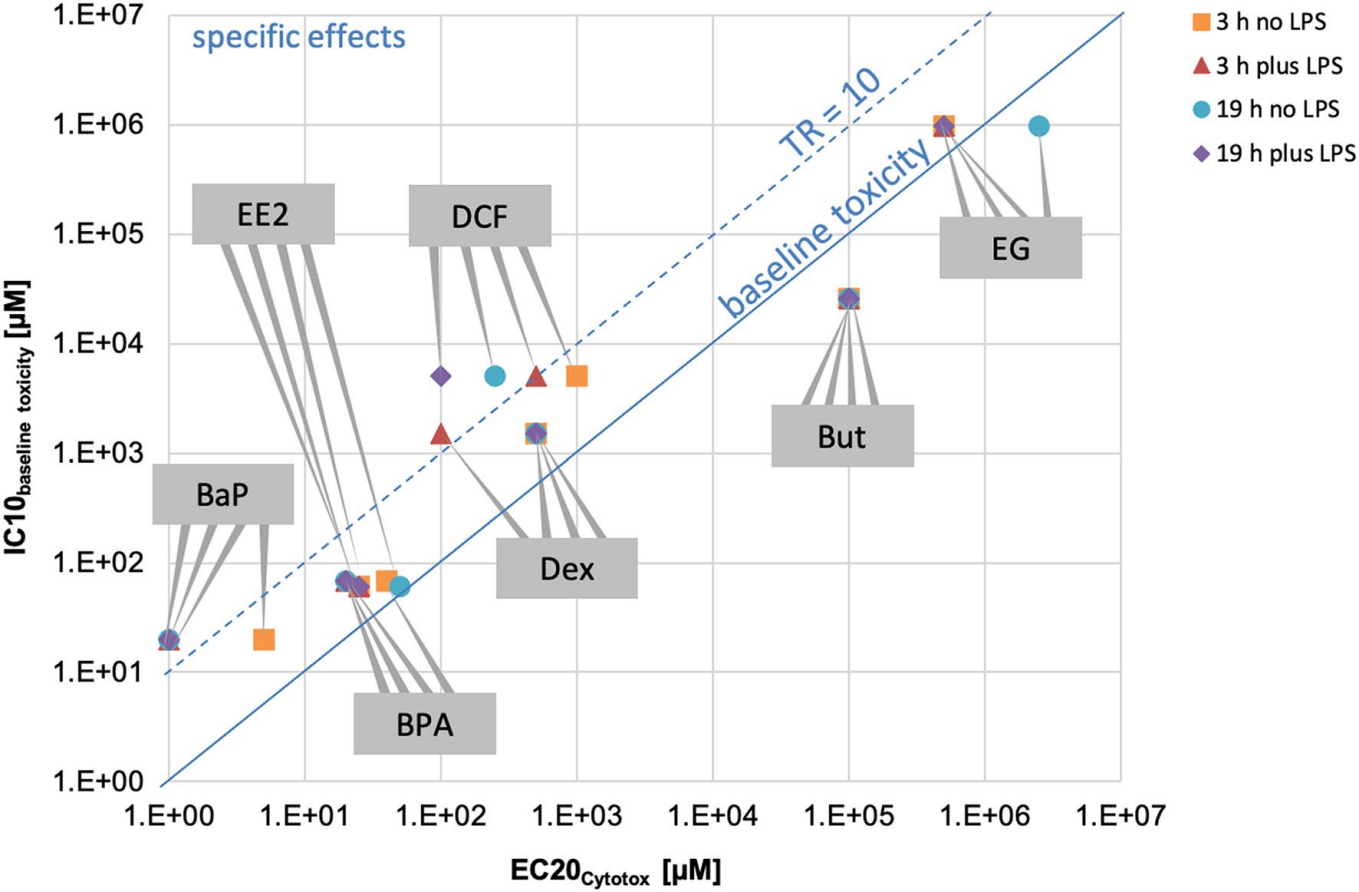
Comparison of the EC20_Cytotox_ for measured cytotoxicity with baseline toxicity predictions (IC10_baseline toxicity_). The immunotoxic chemicals are dexamethasone (Dex), diclofenac (DCF), benzo(a)pyrene (BaP), bisphenol A (BPA) and ethinylestradiol (EE2), the non-immunotoxicants are ethylene glycol (EG) and butanol (But). For BaP the highest tested concentration was used since all chemical concentrations were in the non-cytotoxic range, thus, the EC20_Cytotox_ was not determinable. TR ≤ 10 indicates that cytotoxicity was caused by a non-specific mode of action. *TR* toxic ratio, *EC* effect concentration.

In order to confirm if the immune responses were specific or caused by non-specific toxicity (baseline toxicity or narcosis) we predicted the inhibitory concentrations for baseline toxicity of the test chemicals and compared them to their experimentally determined effect concentrations. All baseline toxicants have a critical membrane concentration of approximately 69 mmol/L_membrane lipid_ that triggers 10% cytotoxicity^51^. This critical membrane concentration can be converted to nominal IC10_baseline_ _toxicity_ with a mass balance model that accounts for the distribution of the chemicals between medium components and uptake into cells and cell membranes^51^. The IC10_baseline_ _toxicity_ depends on the medium composition and the cell make-up in terms of lipid and protein content. Model input parameters such as protein and lipid content were taken from Fischer et al.^63^ and adapted to the cells and medium used here. The distribution constants between protein and water *D*_proteinw_ and between membrane lipids and water *D*_lipw_ at pH 7.4 for all test chemicals are listed in the Supplementary Material S2. This model does not account for evaporative losses, so the trichlorobenzene (TCB), whose loss to air might be as large as 90%, was omitted from this analysis. All other chemicals were not expected to be lost to the headspace (calculated according to Escher et al.^51^, data not shown). The model also does not account for sorption to the plastic of well-plates because, as we recently showed, binding to polystyrene is a kinetically controlled process and in presence of sufficient lipids and proteins the loss is negligible for the chemicals tested because the freely dissolved concentration that would bind to the polystyrene is very small^64^. The EC20_Cytotox_ represents the concentration at which the measured cell viability decreased below 80%—equivalent to < 20% cytotoxicity (EC: effect concentration). This threshold was used to determine the non-cytotoxic concentrations range of test chemicals of the present study and thus, was used for the in silico modelling, too. This experimentally measured cytotoxicity (EC20_Cytotox_) was plotted *versus* the predicted IC10_baseline_ _toxicity_ (Fig. 1). The toxic ratio (TR), which expresses the ratio between calculated IC10_baseline_ _toxicity_ and the measured EC20_Cytotox_ of the present study, was used to identify if the cytotoxicity was caused by a specific MoA (TR > 10) or was non-specific (TR ≤ 10) ^cf.^^65^. The specificity ratio (SR) is an indicator of how much more potent the specific effect of the test chemicals is compared to the cytotoxicity (SR < 10 indicates non-specific action)^52^. The SR of the present study was calculated as the ratio of the EC20_Cytotox_ to the LOEC_immune parameter_. The LOEC_immune parameter_ corresponds to the lowest concentration for which the chemical-induced effect on an immune parameter, compared to the control, had a p value ≤ 0.05 (thus, the LOEC_immune parameter_ was not available for all endpoints and chemicals).

### Data analysis

For the final statistical analysis we assumed that the effects of LPS, the exposure period and the level of chemical concentration act additively to measured immune parameters. Usually, synergy and antagonism come from toxicokinetics and it is unlikely that this is noticeable in pure cell culture systems. In addition, a recently published study demonstrated that below 10% the prediction of concentration addition and independent action are identical^66^. The model applied in the present study includes a different base level of immune system activity for every fish in order to account for differences between the individuals. All other effects are estimated jointly for all fish. The model equation was as follows:

For an immune system activity parameter (or assay) Y, the *i*th observed value is modeled as

$$\begin{aligned} Y_{i} = & \beta_{0} + \mathop \sum \limits_{f \in F}^{{}} \beta_{f} I\left( {{\text{fish}}_{i} = f} \right) + \beta_{\left| F \right| + 1} I\left( {{\text{time}}_{i} = {\text{19h}}} \right) + \beta_{\left| F \right| + 2} I\left( {{\text{LPS}}_{i} = {\text{yes}}} \right) \\ & + \mathop \sum \limits_{c \in C}^{{}} \beta_{\left| F \right| + 2 + c} I\left( {{\text{conc}}_{i} = c} \right) \\ & + \beta_{\left| F \right| + \left| C \right| + 3} I\left( {{\text{time}}_{i} = {\text{19h}}} \right)I\left( {{\text{LPS}}_{i} = {\text{yes}}} \right) \\ & + \mathop \sum \limits_{c \in C}^{{}} \beta_{\left| F \right| + \left| C \right| + 3 + c} I\left( {{\text{time}}_{i} = {\text{19h}}} \right)I\left( {{\text{conc}}_{i} = c} \right) \\ & + \mathop \sum \limits_{c \in C}^{{}} \beta_{\left| F \right| + 2\left| C \right| + 3 + c} I\left( {{\text{LPS}}_{i} = {\text{yes}}} \right)I\left( {{\text{conc}}_{i} = c} \right) + \varepsilon_{i} \\ \end{aligned}$$

where

$${\varepsilon }_{i}\sim N\left(0, {\sigma }^{2}\right).$$

All ${\beta }_{i}$ are parameters to be estimated.

As the effects cannot be seen in isolation, the model includes interaction terms between all effects. This results in a maximum of 17 parameters: one intercept, six for the fish base level, one for the direct LPS effect, one for the effect of the time point, up to three for the concentration level and maximal five for the interactions. A direct interpretation of these model parameters is not intuitive. For this reason, we applied Wald tests to test meaningful linear combinations of the model parameters (e.g.^67^). This enabled us to test the difference between relevant conditions. The models were estimated with the linear model function in R 3.4.1^68^ and the R package “contrast”^69^ was applied to test the linear combination of the parameters. The R-code of the model can be found in the Supplementary Material S3. The LPS treatment served as positive and quality control: results were considered valid and included in the data analysis if LPS caused a stimulation of control cells which was higher than in non-stimulated cells and, additionally, exceeded the technical error (R-code step 2 line 23 in Supplementary Material S3). If those criteria were not fulfilled, fish were excluded from the data analysis. The calculated standard deviations for selecting valid data and the resulting n-numbers for each chemical and immune parameter are listed in the Supplementary Material S4. For the qRT-PCR analysis the statistical analysis was done based on the ΔCt values. Diagnostic plots confirmed that the model assumptions were fulfilled well (see Supplementary Material S5). If the p value was below 0.05 statistical significance was accepted.

## Results

The EC20_Cytotox_ value of each exposure scenario (short- and long-term exposure, without and with LPS stimulation) and test chemical was compared with respective predicted IC10_baseline_ _toxicity_ in Fig. 1 to determine the toxic ratio (TR). The predicted IC10_baseline_ _toxicity_ and the average EC20_Cytotox_ derived from the four exposure scenarios are given in Table 3, the individual values for each exposure scenario are displayed in Fig. 1. A TR ≤ 10 indicates that the cytotoxicity was caused by a non-specific MoA (see above). Since all TR values were below or very close to 10 (TR = 11 for diclofenac), the cytotoxicity of all chemicals was due to non-specific baseline toxicity (Table 3). Results of the cytotoxicity assays are shown in Supplementary Material S6.

**Table 3:** Predicted IC10_Cytotox_, average EC20_Cytotox_ (derived from short- and long-term exposure, without and with LPS) and the calculated toxic ratio (TR).

Test chemical	IC10_baseline toxicity_ (µM)	Average EC20_Cytotox_ (µM)	TR
Dexamethasone (Dex)	1515	400	3.8
Diclofenac (DCF)	5044	463	10.9
Benzo(a)pyrene (BaP)	20	> 2	–
Bisphenol A (BPA)	68	25	2.7
Ethinylestradiol (EE2)	60	31	1.9
Ethylene glycol (EG)	977,000	1,000,000	1.0
Butanol (But)	26,000	100,000	0.3

### Immuno-assay responses to the reference chemical dexamethasone

**Figure 2:**
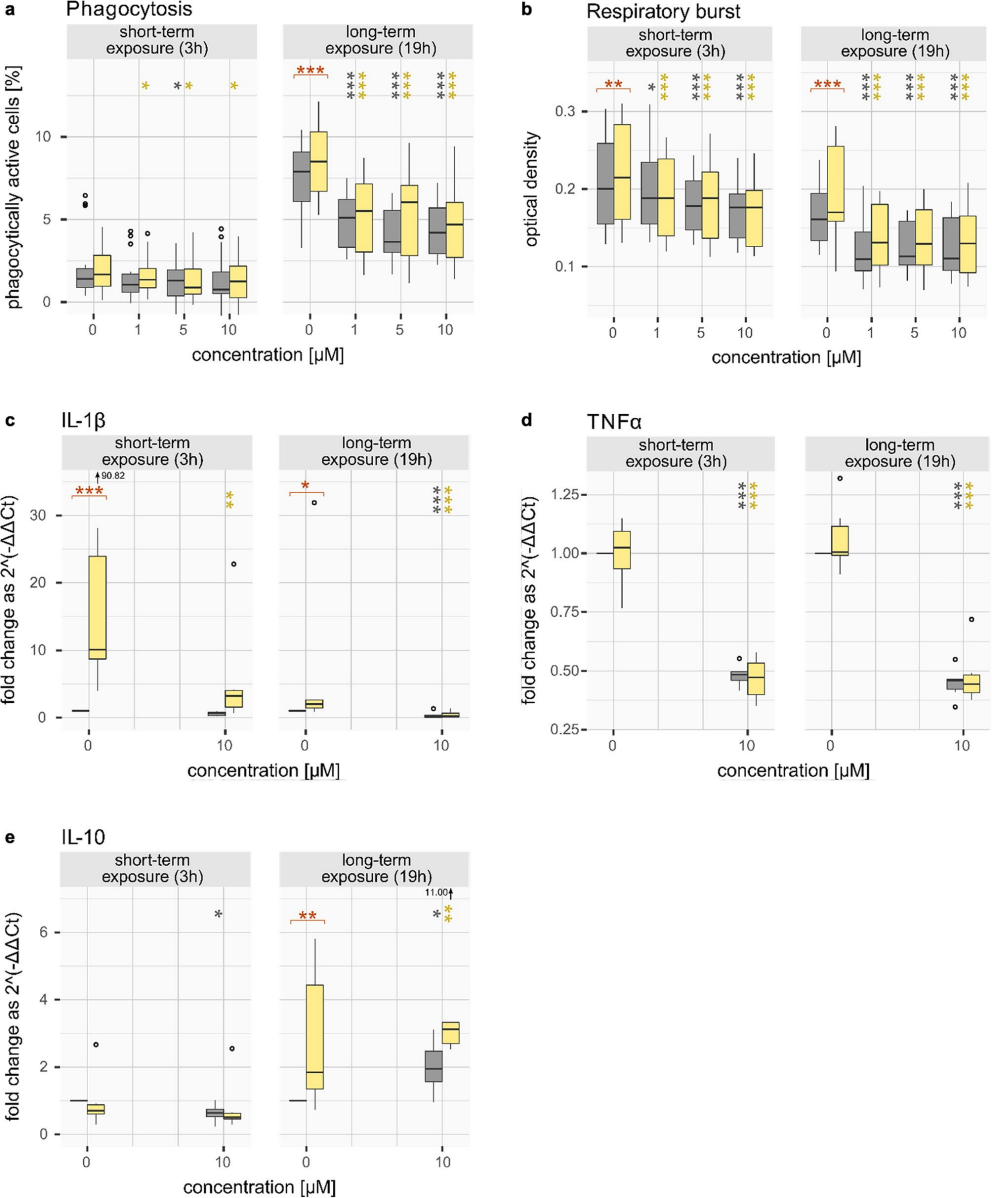
Effects of the reference compound dexamethasone on the immune parameters: phagocytosis (**a**), respiratory burst (**b**) and the mRNA transcript levels of *IL-1β* (**c**), *TNFα* (**d**) and *IL-10* (**e**)*.* Results are shown after short- and long-term exposure with dexamethasone—without (gray) or with (yellow) previous LPS stimulation. Significant differences of the chemical treatments compared to the respective controls are indicated with gray or yellow asterisks, respectively. Significant differences of the control without LPS compared to the control with LPS are indicated with orange asterisks. p values < 0.001 for ***; < 0.01 for ** and < 0.05 for *.

Dexamethasone was used as reference compound as it is a synthetic derivative of cortisol and a well-established immunosuppressive agent for fish. The immune parameter results for dexamethasone are presented as boxplots in Fig. 2. Detailed results of the statistical analyses are provided in Supplementary Materials S7–S9.

#### Phagocytosis

Dexamethasone significantly suppressed the phagocytosis activity in a concentration-dependent manner. This was the case for non-stimulated and for LPS-stimulated leukocytes as well as after short- (p values < 0.05, not significant for non-stimulated cells after 3 h of 1 μM and 10 μM) and long-term exposure (all p values < 0.001, Fig. 2a). LPS caused a significant increase in phagocytosis activity (p value < 0.001 for 19 h).

#### Respiratory burst activity

Dexamethasone significantly suppressed the respiratory burst activity in a concentration-dependent manner. This was the case for non-stimulated and for LPS-stimulated cells as well as after short- and long-term exposure (p value < 0.05 for 3 h, 1 μM without LPS, all other p values < 0.001, Fig. 2b). LPS caused a significant stimulation in the respiratory burst activity (p value < 0.01 and < 0.001 for 3 h and 19 h).

#### Cytokine transcription

Dexamethasone significantly decreased the mRNA transcript levels of the two pro-inflammatory cytokines, *IL-1β* and *TNFα*. This was the case for non-stimulated and for LPS-stimulated cells as well as after short- and long-term exposure (all p values < 0.001, except for *IL-1β*, 3 h, without LPS (not significant) and with LPS (p value < 0.01; Fig. 2c,d)). Transcription of the anti-inflammatory cytokine *IL-10* was significantly reduced in the short-term exposure scenario with non-stimulated cells (p value < 0.05). For the long-term exposure, the *IL-10* mRNA level was significantly increased in stimulated (p value < 0.01) and non-stimulated (p value < 0.05) leukocytes (Fig. 2e). LPS caused a significant induction of the gene transcription for *IL-10* (p value < 0.01 after 19 h) and *IL-1β* (p value < 0.001 and < 0.05 for 3 h and 19 h).

### Immuno-assay responses to all test chemicals

**Figure 3:**
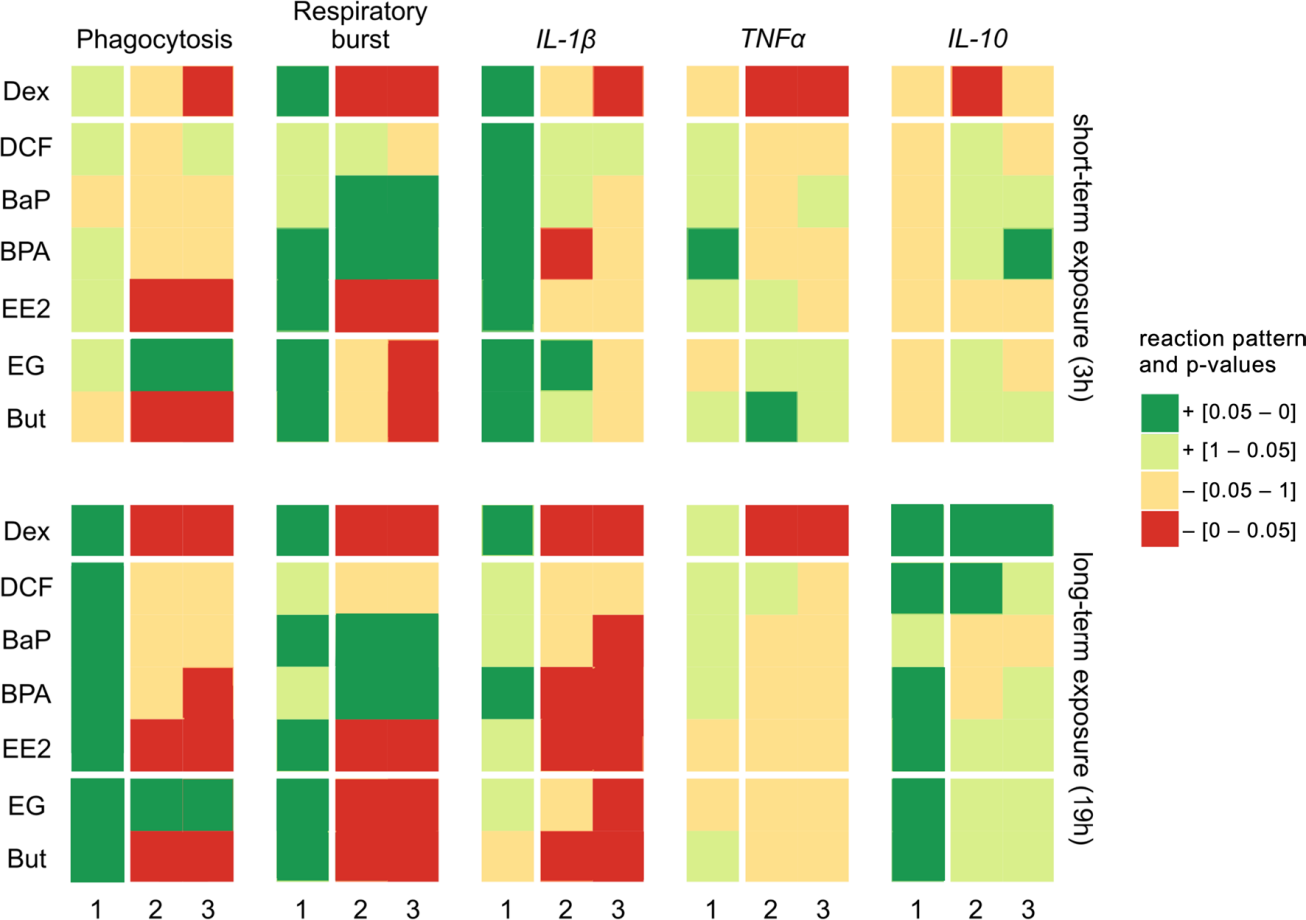
Overview of the in vitro immuno-assay results. Depictured are the results for the comparison of the LPS-stimulated control to the non-stimulated control (column 1 of each immune parameter) and the results for the comparison of the highest applied chemical concentration without (column 2) and with LPS stimulation (column 3) compared to the control without or with LPS stimulation, respectively. Data is displayed for short- (top) and long-term (bottom) chemical exposure separately. Colors represent the p values of the comparisons with underlying decreasing effects in red and for increasing effects in green in relation to the corresponding control. If the p value was below 0.05, statistical significance was accepted. The reference compound dexamethasone (Dex), the other immunotoxicants (*DCF* diclofenac, *BaP* benzo(a)pyrene, *BPA* bisphenol A and *EE2* ethinylestradiol) and the non-immunotoxicants (*EG* ethylene glycol and *But* butanol) were clustered with white dividing lines. A detailed, color-blind-, print-friendly version can be found in Supplementary Material S12.

Results of all test chemicals are summarized in Fig. 3 and provided in detail as boxplots in Supplementary Material S10. Detailed results of the statistical analyses are provided in Supplementary Material S7–S9 and additional information on the response pattern analysis is described in Supplementary Material S11.

#### Phagocytosis

Of the substances known to modulate the fish immune system, the compounds dexamethasone (see above), ethinylestradiol (EE2, p values < 0.01 and < 0.001 for 3 h and 19 h) and bisphenol A (BPA, p value < 0.05 for 19 h) significantly inhibited the phagocytosis activity (Fig. 3, column 2 and 3). For diclofenac (DCF) and benzo(a)pyrene (BaP), a trend towards reduced phagocytosis was observed (Fig. 3, column 2 and 3). Exposure to the non-immunotoxicant ethylene glycol (EG) resulted in a significant stimulation of phagocytosis activity (p values < 0.001 and < 0.05 for 3 h and < 0.001 and < 0.01 for 19 h). Butanol, in contrast, caused a significant inhibition of phagocytosis activity (p values < 0.01 and < 0.001 for 3 h and twice < 0.001 for 19 h). In general, the phagocytosis activity of leukocytes in all experiments was significantly higher after long-term than short-term exposure (p value < 0.001; S7: no. 3, 4, 11, 12), but this was independent of the general response pattern, i.e. if the test chemical lowered phagocytosis activity after short-term exposure, it also did after long-term exposure (Fig. 3). LPS treatment significantly increased phagocytosis activity in all long-term treatments (Fig. 3, column 1; p values < 0.001 except BPA and But with p values < 0.05).

#### Respiratory burst activity

Benzo(a)pyrene (p values < 0.001 for 3 h; < 0.01 and < 0.05 for 19 h) and bisphenol A (p values < 0.001 and < 0.05 for 3 h; twice < 0.001 for 19 h) significantly increased respiratory burst activity (Fig. 3, column 2 and 3). In contrast, ethinylestradiol (p values < 0.001 except p value < 0.01 for non-stimulated cells, 3 h) and dexamethasone (see above) exposure resulted in a significant inhibition of respiratory burst activity without and with LPS stimulation at both time points. Diclofenac had no significant effects. The non-immunotoxicants, ethylene glycol (p value < 0.05 for stimulated cells, 3 h; < 0.05 and < 0.01 for 19 h) and butanol (p value < 0.01 for stimulated cells, 3 h; < 0.05 and < 0.001 for 19 h), resulted in a significant inhibition of respiratory burst activity for both exposure periods. This effect was stronger in LPS-stimulated leukocytes. LPS significantly stimulated the respiratory activity (p values < 0.05 for But and EE2 (19 h), p values < 0.01 for BPA (3 h), EE2 (3 h), BaP (19 h), p values < 0.001 for EG; Fig. 3, column 1).

#### Cytokine transcription

For *IL-1β* transcription, significant inhibitions were detected after exposure to dexamethasone (see above), benzo(a)pyrene (p value < 0.001 for stimulated cells, 19 h), bisphenol A (p values < 0.01 for non-stimulated cells at 3 h, < 0.001 and < 0.05 for 19 h) and ethinylestradiol (p values < 0.05 and < 0.01 for 19 h) as well as after exposure to ethylene glycol (p values < 0.001 for stimulated cells, 19 h) and butanol (p values < 0.01 and < 0.001 for 19 h). The inhibition was more pronounced after long-term exposure than after short-term exposure (Fig. 3, column 2 and 3). *TNFα* transcription was significantly stimulated by butanol, after 3 h in non-stimulated cells (p value < 0.01) but inhibited by dexamethasone (see above). The mRNA transcript level of *IL-10* was significantly altered by dexamethasone (see above) and increased in response to bisphenol A (p value < 0.05 for stimulated cells, 3 h) and diclofenac (p value < 0.05 for non-stimulated cells, 19 h).

**Figure 4:**
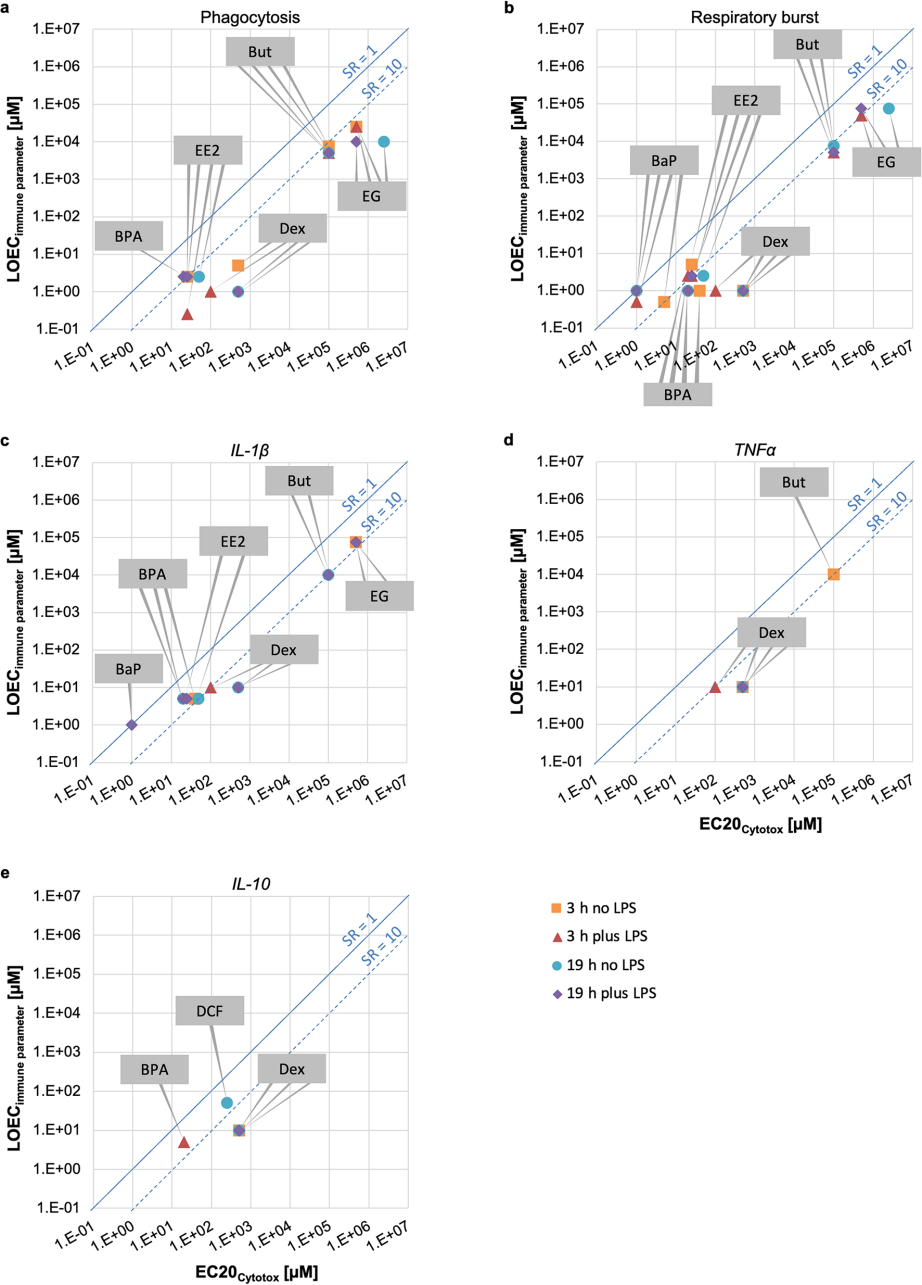
Comparison of the LOEC_immune parameter_ with the experimental cytotoxicity EC20_Cytotox_. The immunotoxicants are dexamethasone (Dex), diclofenac (DCF), benzo(a)pyrene (BaP), bisphenol A (BPA) and ethinylestradiol (EE2); non-immunotoxicants are ethylene glycol (EG) and butanol (But). The LOEC_immune parameter_ was not available for all endpoints and chemicals since only chemical-induced effect on immune parameter with a p value ≤ 0.05 were considered. SR > 10 indicates specificity for chemical effects on immune parameter. *SR* specific ratio, *LOEC* lowest observed effect concentration, *EC* effect concentration.

The LOEC_immune parameter_ were compared with the respective EC20_Cytotox_ for each exposure scenario (short- and long-term exposure, without and with LPS stimulation) to determine the specific ratio (SR, Fig. 4; numerical values listed in Table 4). The SR indicates how much more potent the immunotoxic activity of the test chemicals is compared to its cytotoxic activity. The average LOEC_immune parameter_ of the four exposure scenarios (short- and long-term exposure, without and with LPS stimulation) is given for each immune parameter and test chemical in Table 4, the average EC20_Cytotox_ for the four exposure scenarios in Table 3. The reference compound dexamethasone acted highly specific on the phagocytosis and respiratory burst activity, as indicated by SR > 100. Almost all other chemicals showed an SR > 10, being moderately specific on the phagocytosis (except BPA with SR = 8). For respiratory burst activity, SRs were lower than for phagocytosis activity, but mostly above 10. In contrast, SR values for the transcript level of the three cytokines had low SRs for the reference compound dexamethasone (SR ≤ 50) and did not show SR > 10 for the other test compounds, suggesting that chemical action was rather non-specific for those parameters.

**Table 4:** Average LOEC_immune parameter_ and average specific ratios (SR) for the immune parameters. The averages are derived from the values for short- and long-term exposure, each without and with LPS.

Test chemical	Phagocytosis activity		Respiratory burst activity		IL-1β transcription		TNFα transcription		IL-10 transcription	
	Average LOEC_immune parameter_ (µM)	Average SR	Average LOEC_immune parameter_ (µM)	Average SR	Average LOEC_immune parameter_ (µM)	Average SR	Average LOEC_immune parameter_ (µM)	Average SR	Average LOEC_immune parameter_ (µM)	Average SR
Dexamethasone (Dex)	2	300	1	400	10	37	10	40	10	50
Diclofenac (DCF)	n.d		n.d		n.d		n.d		50	5
Benzo(a)pyrene (BaP)	n.d		0.75	3.5	1	1	n.d		n.d	
Bisphenol A (BPA)	2.5	8	1.4	22	5	5	n.d		5	4
Ethinylestradiol (EE2)	2	35	3	11	5	7.5	n.d		n.d	
Ethylene glycol (EG)	17,500	85	66,667	17	75,000	7	n.d		n.d	
Butanol (But)	5,625	18	5,833	18	10,000	10	10,000	10	n.d	

*n.d.* not determinable, *SR* specific ratio, *LOEC* lowest observed effect concentration.

## Discussion

The aim of the present study was to examine if an in vitro assay using isolated fish leukocytes is suitable to screen for immunotoxic potentials of environmental chemicals to fish and to discriminate those against chemicals with no known immunotoxic action. We exposed the leukocytes at non-cytotoxic concentrations in a short- (3 h) and long-term (19 h) exposure scenario, each without and with previous LPS stimulation. As endpoints indicative of immunotoxic activity of the test compounds, we selected those parameters that were identified in a previous review^45^ as the most frequently measured parameters in fish immunotoxicity studies: phagocytosis activity, oxidative burst activity and cytokine production. While the former two parameters could be stably measured in our in vitro assay, the cytokine transcript levels showed high interindividual variability what limits their value as immunotoxicity indicators but does not affect the overall conclusion of our study.

Differences between short- and long-term exposures were found particularly for the phagocytosis activity and the cytokine responses. In the case of the immune gene transcription the difference between short- and long-term exposures is probably due to biological mechanisms such as the involvement of different signaling pathways: for instance, time-dependent differences of the immunomodulating effects of ER-ligands have been reported for carp leukocytes. The time dependency may relate to the differential activation of (fast) non-genomic and (slow) genomic signaling pathways^70^. The effects seen for the phagocytosis activity are likely due to a technical issue: after 3 h of bead-exposure the percentage of phagocytosis-active cells in the total cell population was still very low and this translated into an instable measurement signal. After 19 h the percentage of phagocytosis-active cells had clearly increased, giving rise to a more stable signal.

The LPS-treatment of the leukocytes caused stimulation of the phagocytosis, respiratory burst activity and cytokine transcripts. Our findings of the LPS effects on the immune parameters are in line with results of other studies and reflect the stimulatory action of bacterial antigens on the immune cells^71–80^. Thus, LPS treatment could serve as positive and quality control for the validity of cell isolations (see “Data analysis”).

The reference compound, dexamethasone, is an activator of the glucocorticoid receptor (GR) pathway. In our experiments, dexamethasone induced the expected responses. Regulation of GR by glucocorticoids in fish leukocytes has been demonstrated previously^81,82^. The significant inhibition of phagocytosis and respiratory burst activity observed in our study is in line with findings of other studies (e.g.^31–33,83–85^). For the transcript levels of the cytokines we observed a reduction of the pro-inflammatory *IL-1β* and *TNFα*, together with a significant stimulation of the anti-inflammatory *IL-10*. The anti-inflammatory IL-10 is mainly involved in the attenuation of activated immune responses, in contrast to the pro-inflammatory cytokines^86–90^ thus, the observed opposite reaction patterns of the pro- and anti-inflammatory cytokines were to be expected. Together with this LPS results the findings from the dexamethasone experiments confirm the validity of the in vitro assay as performed in the present study.

Ethinylestradiol and bisphenol A displayed immunomodulating activities in the in vitro leukocyte assay. In line with our findings, these two estrogen-active compounds were found to influence the immune system of fish in a number of previous studies^28,91–97^. They mediate their effects probably via estrogen receptors (ER), which are known to be expressed in fish immune cells^27,70,95,96,98^.

Benzo(a)pyrene significantly stimulated the respiratory burst activity and significantly inhibited the transcription of *IL-1β* in the in vitro immuno-assay. Benzo(a)pyrene has a well-documented immunomodulating activity in fish including rainbow trout^16–18^. Its MoA involves the activation of the arylhydrocarbon receptor (AhR) which is a central regulator of various immune pathways and functions^99^ and known to be expressed in fish leukocytes^100–102^. Moreover, our findings on the immunomodulating properties of selected ER and AhR ligands in isolated trout leukocytes are corroborated by findings on ER- and AhR-mediated effects in other fish species such as sea bream, rare minnow and carp^22,71,103–105^.

Diclofenac had a weak effect in our in vitro immuno-assay. This was unexpected since diclofenac is a widely used pain killer with anti-inflammatory and immunosuppressant properties (e.g.^106^). Studies with fish demonstrated that it indeed modifies the expression level of cyclooxygenase (COX) and further immune parameters^30,32,107^. Nevertheless, evidence from mammalian studies suggest that diclofenac may not directly interfere with phagocytotic and respiratory activity. In cultured mice macrophages, COX inhibitors such as diclofenac enhanced zymosan-stimulated phagocytosis but had only a minor effect on the respiratory burst^108^. Neumüller and Tohidast-Akrad^109^ found only a slight increase in phagocytosis activity after exposing human peripheral blood leucocytes to diclofenac. In an invertebrate species, the freshwater mussel *Elliptio complanate*, no correlation between the effect of diclofenac on COX and phagocytosis could detected^110^. The finding that diclofenac had only a weak effect in our leukocyte assay highlights how important it is to consider the application domain of an assay: the assay used in the present study appear to be well able to screen for chemicals such as dexamethasone, ethinylestradiol, bisphenol A or benzo(a)pyrene that exert their immunological activity through receptors, but it may be less suitable for chemicals such a diclofenac which modulate the immune cells through other MoA.

The non-immunotoxicants, butanol and ethylene glycol, induced significant responses of at least some of the immune parameters. We tested a third baseline toxicant, 1,2,4-TCB, which was retrospectively excluded from the study. Due to its high volatility, the active concentration of TCB in the assay was difficult to control. Nevertheless, TCB, like butanol and ethylene glycol, evoked reactions in the immuno-assay (Supplementary Material S10). The observation that these baseline toxicants with no known immune activity still induce responses of the immune parameters in the in vitro leukocyte assay is in contradiction to our original expectation that the assay would discriminate between immunotoxicants and non-immunotoxicants. On the contrary, the in vitro assay would falsely classify ethylene glycol, butanol and TCB as potential immunotoxicants. It is acceptable that a screening assay produces false positive classifications, but the question is what percentage of false positives is acceptable? A too high percentage would disqualify the assay. On the basis of our study this question cannot be conclusively answered. It would need an extended testing of additional chemicals to conclude on the specificity of the in vitro fish leukocyte assay.

Is there a possible explanation for this lack of discrimination between immunotoxicants and non-immunotoxicants by the in vitro assay? The simplest explanation would be that we tested at concentrations that cause cytotoxicity. This, however, does not apply since we selected non-cytotoxic concentrations. Thus, other mechanisms must be responsible for the immunological activities of low concentrations of narcotic chemicals in the leukocyte assay. The assumption is supported by our in silico modeling as the SR for the non-immunotoxicants indicated a rather specific but not a cytotoxicity-driven action. Since we did not find any indication in the literature that those chemicals act via direct interference with immune system components, the observed effects might be rather based on an indirect action. Baseline or narcotic toxicity arises from the non-specific intercalation of these chemicals into the (phospho-)lipid layers of biological membranes thereby interfering with their normal functioning^111^. At high concentrations the chemical-induced disturbances lead to the loss of the barrier function of the cell membrane, eventually resulting in cell death. At low concentrations, the induced alterations of the cell membrane might be subtle but sufficient to cause osmotic stress to the cell and, associated with this, the modulation of cell membrane-associated signaling cascades such as ERK (extracellular signal-regulated kinases), JNK (c-Jun N-terminal kinase), MAPK (mitogen-activated protein kinase) or NFAT5 (Nuclear factor of activated T-cells 5)^112–114^. These signaling cascades function as stress sensors of the cell^115–117^ and they often converge with NF-κB (Nuclear factor kappa-light-chain-enhancer of activated B-cells) signaling. NF-κB is involved in the cellular stress response^117^ but at the same time is a central regulator of cellular immune functions^118–120^. Besides, cell membrane disturbances can activate the heat shock/chaperone systems which can modulate immune pathways^121^ or it may affect special regions of the cell membrane, the so-called lipid rafts. Lipid rafts were shown to be important organizing elements for immune signaling receptors^122^. Hence, the observed immune effects of the non-immunotoxicants may be due to indirect mechanisms mediated via a non-specific disturbance of the membrane rather than via specific and targeted immunotoxic MoAs. In the present study, only three narcotics were tested thus, the aforementioned considerations are highly speculative, but it may be worthwhile to investigate the suggested mechanism in future studies.

In conclusion, the present study provides a systematic evaluation of a potential fish immunotoxicity screening assay. A set of test compounds containing non-immunotoxic chemicals and toxicants with different modes of immunotoxic action were assessed under the same test conditions using identical effect parameters. From the results it appears that the in vitro fish leukocyte assay, as performed in the present study, has only a limited capacity for discriminating between immunotoxicants and non-immunotoxicants. Thus, this assay is not suitable as “stand alone assay” for immunotoxicity screening. Since the fish immune system is too complex to be represented by only one assay, it will need a battery of complementary assays (cf.^34^), as it is realized in the guidelines for human immunotoxicity testing^123^. To identify a test battery for fish immunotoxicity screening the systematic evaluation as conducted in the present study needs to be applied to further candidate parameters and assays.

## Supplementary Information

Supplementary Information
