# Supplementary material for: Evaluation of an in vitro assay to screen for the immunotoxic potential of chemicals to fish
Source: Sci Rep. 2021 Feb 4;11:3167. doi: 10.1038/s41598-021-82711-5 (PMC7862612; doi:10.1038/s41598-021-82711-5)
Supplement: Supplementary file 1 — Supplementary Information [file 41598_2021_82711_MOESM1_ESM.pdf]

Supplementary material related to the following article:

Evaluation of an *in vitro* assay to screen for the immunotoxic potential of chemicals to fish

Kristina Rehberger[1*], Beate I. Escher[2,3], Andreas Scheidegger[4], Inge Werner[5], Helmut Segner[1]

[1] Centre for Fish and Wildlife Health, Vetsuisse Faculty, University of Bern, Bern, Switzerland

[2] UFZ-Helmholtz Centre for Environmental Research, Leipzig, Germany

[3] Centre for Applied Geoscience, Eberhard Karls University Tübingen, Tübingen, Germany

[4] Eawag: Swiss Federal Institute of Aquatic Science and Technology, Dübendorf, Switzerland

[5] Swiss Centre for Applied Ecotoxicology, Eawag, Dübendorf, Switzerland

[*]kristina.rehberger@vetsuisse.unibe.ch

Content

S1: Additional information on material and methods 2
S2: Physicochemical parameters for in silico predictions 4
S3: R-Model 5
S4: Standard deviation and fish n-number 12
S5: Diagnostic plots 14
S6: Cytotoxicity assays to determine cell viability 30
S7: p-values 34
S8: Test statistics 37
S9: Approaches for the data analysis / statistic 40
S10: Boxplots for each test compound 41
S11: Assay response patterns 49
S12: Heat map 49

S1: Additional information on material and methods

a) Key words for the literature search

Butanol+immun; Butanol+immun+fish; 1-butanol+immun; 1-butanol+immun+fish; TCB+immun; TCB+immun+ fish; Trichlorobenzene+immun; Trichlorobenzene+immun+fish; 1,2,4-Trichlorobenzene+immun; 1,2,4-Trichlorobenzene+immun+fish; Ethylene glycol+immun; Ethylene glycol+immun+fish (Google scholar; Dec. 2017)

b) Reagents

Sigma-Aldrich Switzerland: Benzo(a)pyrene: B1760, Bisphenol A: 239658, 1-Butanol: 19422, C-AM: 17783, DAPl: D9542, Dexamethasone: D4902, Diclofenac: 93484, 17α-Ethinylestradiol: E4876, Ethylene glycol: 85978, fluorescent-labelled latex beads 1 µm: L4655 (washed before application), HEPES: H4034, Lipopolysaccharide: L4391, $NaHCO_3$ (Fulka *via* Sigma): 71630, NBT: N6876, Percoll: P4937, propidium iodide: P4170, trypan blue: T6146, 1,2,4-Trichlorobenzene: 296104, RPMI medium R8755. Gibco by Life Technologies for trypsin 15400054. Mesh used for isolation: Sefar Petex 07-105/52 (3A07-0105-106-00)

c) qRT-PCR information

Each sample composed 6.25 µl of the GoTag qPCR MasterMix, 0.625 µl of 10 µM forward and reverses primer mix (final 500 nM each), 3.625 µl nuclease-free water and 2 µl cDNA. For the NTC, no cDNA but water was added. Elongation Factor 1 alpha (*EF-1α*) and *18S* were run as reference genes based on a literature review. For *18S*, the cDNA was diluted 1:500. Although the expression of 18S was stable, the expression of EF-1*α* was more stable and thus, latter was used for normalization of the cytokine expression. The cytokines *IL-1β, TNFα* and *IL-10* were selected based on Rehberger et al. 2017.

d) Mean Ct values for *EF-1a* of each test chemical (alphabetic order)

Legend

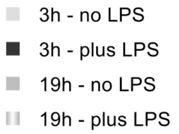

Benzo(a)pyrene

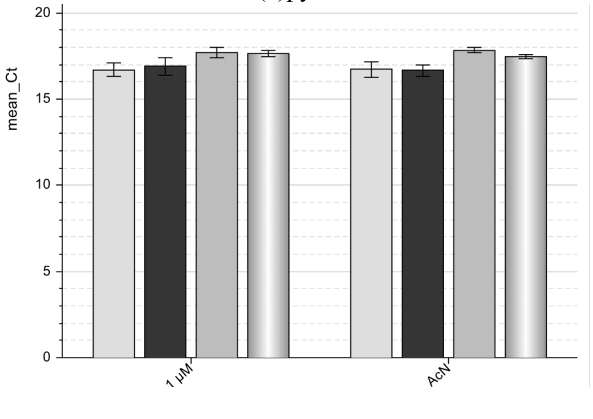

Bisphenol A

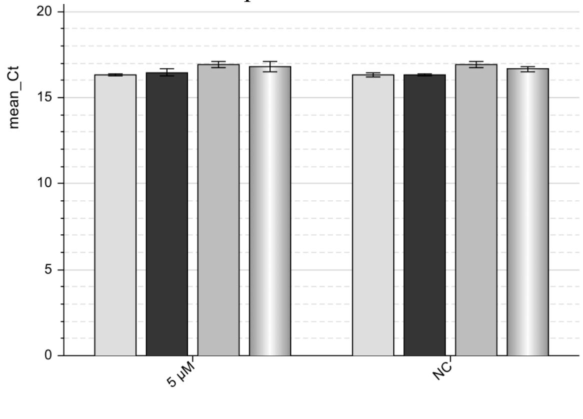

Butanol

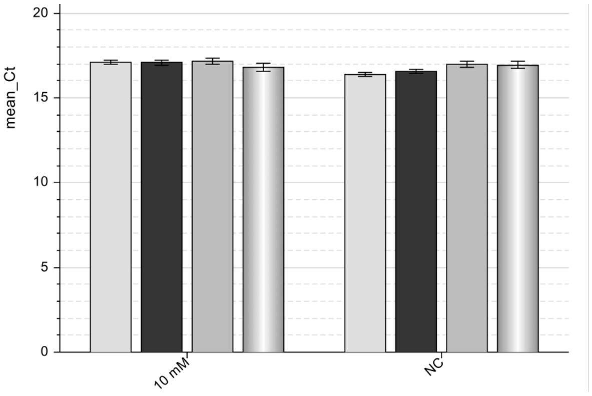

Ethinylestradiol

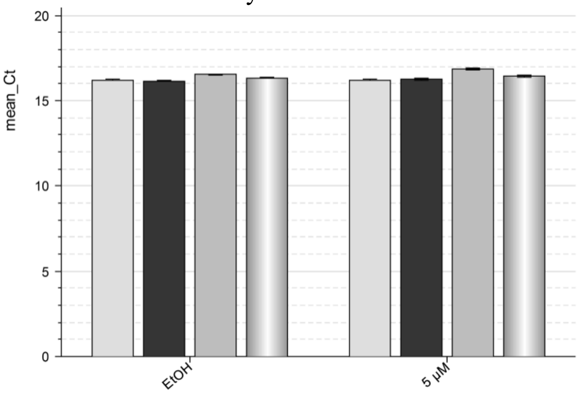

Diclofenac

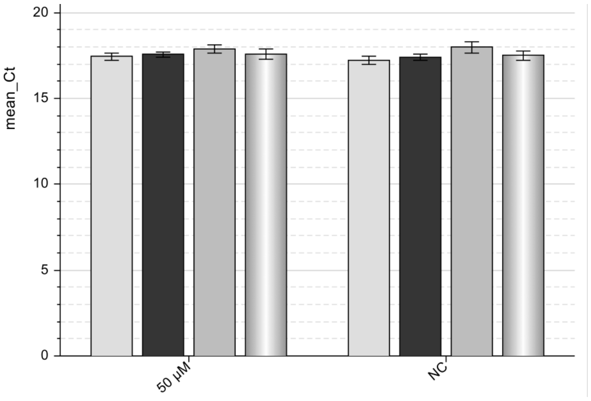

Ethylene glycol

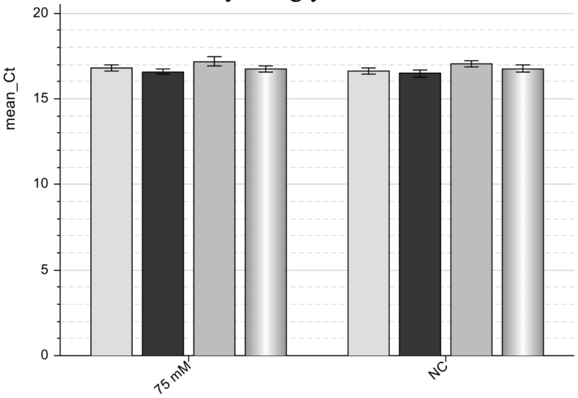

Dexamethasone

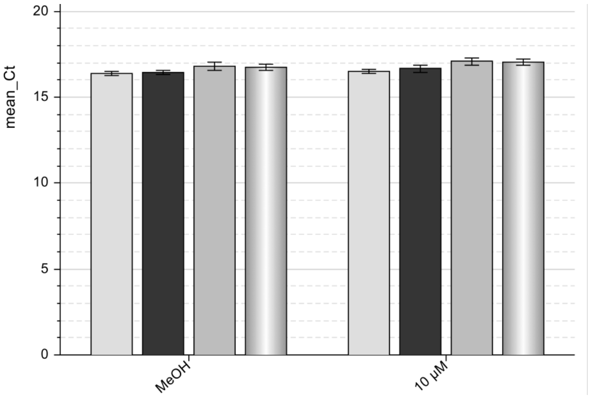

Trichlorobenzene

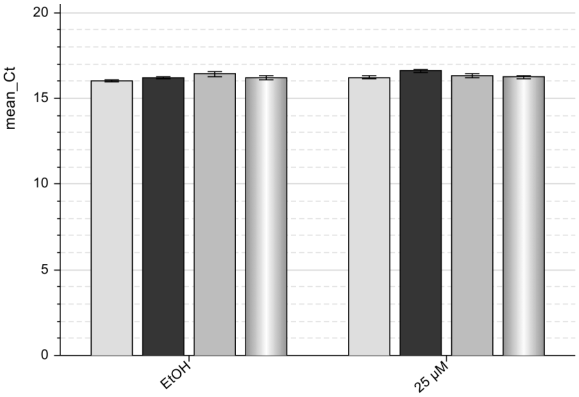

S2: Physicochemical parameters for *in silico* predictions

Tab. S2 Partition coefficients for liposome-water (D_{lipw}) and protein-water ($D_{proteinw}$)

**Test chemicals**	**log D_{lipw} [L/L] pH 7.4**	**Reference**	**log $D_{proteinw}$ [L/L] pH 7.4**	**Reference**
Dexamethasone (Dex)	1.73	PP-LSER prediction*	2.12	PP-LSER prediction*
Diclofenac (DCF)	2.64	Avdeef, A.; Box, K. J.; Comer, J. E. A.; Hibbert, C.; Tam, K. Y., pH-Metric logP 10. Determination of Liposomal Membrane-Water Partition Coefficients of lonizable Drugs. Pharm. Res. 1998, 15, 209-215.	4.40	Henneberger L, Mühlenbrink M, Fischer FC, Escher BI. 2019. C18-Coated Solid-Phase Microextraction Fibers for the Quantification of Partitioning of Organic Acids to Proteins, Lipids, and Cells. Chem Res Toxicol 32, 168-178.
Benzo(a)pyrene (BaP)	7.05	van der Heijden, S. A.; Jonker, M. T. O. Evaluation of liposome-water partitioning for predicting bioaccumulation potential of hydrophobic organic chemicals. Environ. Sci. Technol. 2009, 43, 8854-8859.	5.05	PP-LSER prediction*
Bisphenol A (BisA)	3.50	Kwon, J.-H.; Liljestrand, H. M.; Katz, L. E., Partitioning Thermodynamics of Selected Endocrine Disruptors between Water and Synthetic Membrane Vesicles: Effects of Membrane Compositions. Environmental Science & Technology 2007, 41, 4011-4018.	3.01	Endo, S.; Goss, K. U., Serum Albumin Binding of Structurally Diverse Neutral Organic Compounds: Data and Models Chem. Res. Toxicol. 2011, 24, 2293-2301.
17α-Ethinylestradiol (EE2)	3.85	PP-LSER prediction for estradiol*	3.42	PP-LSER prediction for estradiol*
1,2,4-Trichlorobenzene (TCB)	4.22	PP-LSER prediction*	3.47	PP-LSER prediction*
Ethylene glycol (EG)	-1.15	PP-LSER prediction*	-0.69	PP-LSER prediction*
1-Butanol (But)	0.45	Vaes, W.H.J., Ramos, E.U., Hamwijk, C., vanHolsteijn, I., Blaauboer, B.J., Seinen, W., Verhaar, H.J.M. and Hermens, J.L.M. (1997). Solid phase microextraction as a tool to determine membrane/water partition coefficients and bioavailable concentrations in in vitro systems. Chemical Research in Toxicology, 10, 1067-1072.	0.91	PP-LSER prediction*

*Ulrich N, Endo S, Brown TN, Watanabe N, Bronner G, Abraham MH, Goss K-U. 2018. UFZ-LSER database v 3.2.1 [Internet], Leipzig, Germany, Helmholtz Centre for Environmental Research-UFZ. 2017 [accessed on 20.07.2018]. Available from http://www.ufz.de/lserd

S3: R-Model

```
library(contrast)
library(dplyr)
write.textfile <- TRUE
all.compounds <- c("BaP", "BPA", "But", "DCF", "Dex", "EE2", "EG", "TCB")

     ## loop over all compounds
for(compound in all.compounds) {
    print(paste("-- Compound:", compound))

    ## 1) import data
    dat <- read.table(paste0("data/", compound, "_raw data2.csv"), header=T, sep=";")

    ## reorder levels
    if(any(levels(dat$LPS) != c("LPS", "no LPS"))) stop("check csv file!!!")
    levels(dat$LPS) <- c("yes", "no")
    dat$LPS <- relevel(dat$LPS, "no")
    dat$time <- relevel(dat$time, "3h")
    dat$conc <- as.factor(as.numeric(gsub("([0-9\\.]+).*$", "\\1",
                                          as.character(dat$conc))))
    dat$compound <- as.character(dat$compound)

    ## 2) add "select" columns for each assay -> select valid data

    ## calculate averaged standard deviations of all technical errors
    sd.tech <- dat %>% group_by(Fish, LPS, time, conc) %>%
        summarize(sd.i.tech.NBT=sd(NBT), sd.i.tech.PhagoG=sd(PhagoG), n=n()) %>%
        ungroup() %>%
        summarize(NBT=mean(sd.i.tech.NBT, na.rm=T),
                  PhagoG=mean(sd.i.tech.PhagoG, na.rm=T)) %>%
        as.data.frame()

    ## accepted probability that v1 < v2
    conf.level <- 0.2

    ## keep other assays if 3h control or 19 control has reacted
    for(assay in c("NBT", "PhagoG")) {
        dat[[(paste0(assay, ".included"))]] <- NA
        for(fish in levels(dat$Fish)) {

            ## 3h test
            v1 <- dat %>% filter(LPS=="yes", Fish==fish, time=="3h", conc=="0") %>%
                pull(assay)
            v2 <- dat %>% filter(LPS=="no", Fish==fish, time=="3h", conc=="0") %>%
                pull(assay)
            ## test probability to have a real effect
            p.neg <- pnorm(0, mean(v1-v2), sqrt(2)/sqrt(length(v1))*sd.tech[1,assay])
            include.3h <- p.neg < conf.level
            include.3h[is.na(include.3h)] <- FALSE

            ## 19h test
            v1 <- dat %>% filter(LPS=="yes", Fish==fish, time=="19h", conc=="0") %>%
                pull(assay)
            v2 <- dat %>% filter(LPS=="no", Fish==fish, time=="19h", conc=="0") %>%
                pull(assay)
            ## test probability to have a real effect
            p.neg <- pnorm(0, mean(v1-v2), sqrt(2)/sqrt(length(v1))*sd.tech[1,assay])
            include.19h <- p.neg < conf.level
            include.19h[is.na(include.19h)] <- FALSE

            ## write include
            dat[dat$Fish==fish, paste0(assay, ".included")] <- include.3h | include.19h
        }
    }
    for(assay in c("IL10.dCt", "TNFa.dCt", "IL1B.dCt")) {
        dat[[(paste0(assay, ".included"))]] <- NA
        for(fish in levels(dat$Fish)) {
```

```
            ## 3h test
            v1 <- dat %>% filter(LPS=="yes", Fish==fish, time=="3h", conc=="0") %>%
                pull(assay)
            v2 <- dat %>% filter(LPS=="no", Fish==fish, time=="3h", conc=="0") %>%
                pull(assay)
            ## test probability to have a real effect
            include.3h <- mean(v1, na.rm=T) < mean(v2, na.rm=T)
            include.3h[is.na(include.3h)] <- FALSE

            ## 19h test
            v1 <- dat %>% filter(LPS=="yes", Fish==fish, time=="19h", conc=="0") %>%
                pull(assay)
            v2 <- dat %>% filter(LPS=="no", Fish==fish, time=="19h", conc=="0") %>%
                pull(assay)
            ## test probability to have a real effect
            include.19h <- mean(v1, na.rm=T) < mean(v2, na.rm=T)
            include.19h[is.na(include.19h)] <- FALSE

            ## write include
            dat[dat$Fish==fish, paste0(assay, ".included")] <- include.3h | include.19h
        }
        dat[is.na(dat[,assay]), paste0(assay, ".included")] <- FALSE
    }

    ## keep other endpoints / assay if NBT or PhagoG reacted (was only tested)
    ##    dat$"IL10.dCt.included" <- dat$"TNFa.dCt.included" <- dat$"IL1B.dCt.included" <-
dat$NBT.included | dat$PhagoG.included

    ## print selected Fish
    cat("\nstandard deviation technical errors:\n")
    print(round(sd.tech,4))
    for(assay in c("NBT", "PhagoG", "IL10.dCt", "TNFa.dCt", "IL1B.dCt")) {
        select <- dat[,paste0(assay, ".included")] & !is.na(dat[,assay])
        cat("Selected for", assay, ": ",
            as.character(unique(dat$Fish[select])), "\n")
    }

    ## 3) fit models for each assay
    mycontrast <- function(fit, a, b, ...) {
        k <- contrast(fit, a, b, ...)
        cat(paste(paste(names(a), a, sep="="), collapse=", "), "\n")
        cat(paste(paste(names(b), b, sep="="), collapse=", "), "\n\n")
        print(k)
        return(k)
    }
    if(write.textfile){
        sink(paste0("results/" , compound, "_model_summaries.txt"))
    }
    cat("==========================================\n")
    cat(" Models for", dat$compound[1], "\n")
    cat("==========================================\n\n")
    models <- list()
    for(assay in c("NBT", "PhagoG", "IL10.dCt", "TNFa.dCt", "IL1B.dCt")) {

        ## select the valid data points
        select <- dat[,paste0(assay, ".included")] & !is.na(dat[,assay])

        ## fit model
        ff <- reformulate("(LPS+time+conc)^2 + Fish", assay)
        ## ff <- reformulate("LPS*time*conc + Fish", assay)  # with all interactions was tested, too
        cat(paste("\nFit this model for", assay, ":\n"))
        print(ff)
        ## fit the linear model
        mod <- lm(ff, data=dat[select,])
        cat("\n\n=====================")
        cat("\n", assay, "\n")
        cat("\n-----------\n1.) Summary\n")
        print(summary(mod))
```

```
## save model and select vector to list
models[[assay]] <- list(model=mod, select=select)

## calculate contrasts (i.e. tests)
## -- calculate & print contrasts
cat("\n-----------\n2.) Contrasts\n")

## Select a fish. It doesn't matter which one,
## because we don't have interactions with fish.
a.Fish <- levels(droplevels(dat$Fish[select]))[1]

## select all conc levels
all.conc.levels <- levels(droplevels(dat$conc[select]))
n.conc.levels <- table(droplevels(dat$conc[select]))
#max.level <- all.conc.levels[length(all.conc.levels)]
tests <- list()

## Comparisons:
## 1) LPS induction (3h)?
cat("\n2.1 ---\n")
a <- list(time="3h", LPS="no", conc="0", Fish=a.Fish)
b <- list(time="3h", LPS="yes", conc="0", Fish=a.Fish)
tests$"(01)" <- mycontrast(mod, a, b)

## 2) LPS induction (19h)?
cat("\n2.2 ---\n")
a <- list(time="19h", LPS="no", conc="0", Fish=a.Fish)
b <- list(time="19h", LPS="yes", conc="0", Fish=a.Fish)
tests$"(02)" <- mycontrast(mod, a, b)

## 3) time differences (noLPS)?
cat("\n2.3 ---\n")
a <- list(time="3h", LPS="no", conc="0", Fish=a.Fish)
b <- list(time="19h", LPS="no", conc="0", Fish=a.Fish)
tests$"(03)" <- mycontrast(mod, a, b)

## 4) time differences (yesLPS)?
cat("\n2.4 ---\n")
a <- list(time="3h", LPS="yes", conc="0", Fish=a.Fish)
b <- list(time="19h", LPS="yes", conc="0", Fish=a.Fish)
tests$"(04)" <- mycontrast(mod, a, b)

## 5) compound effect (3h, noLPS)?
cat("\n2.5 ---\n")
a <- list(time="3h", LPS="no", conc="0", Fish=a.Fish)
b <- list(time="3h", LPS="no", conc=all.conc.levels[-1], Fish=a.Fish)
tests$"(05)" <- mycontrast(mod, a, b)

## 6) compound effect (3h, yesLPS)?
cat("\n2.6 ---\n")
a <- list(time="3h", LPS="yes", conc="0", Fish=a.Fish)
b <- list(time="3h", LPS="yes", conc=all.conc.levels[-1], Fish=a.Fish)
tests$"(06)" <- mycontrast(mod, a, b)

## 7) compound effect (19h, noLPS)?
cat("\n2.7 ---\n")
a <- list(time="19h", LPS="no", conc="0", Fish=a.Fish)
b <- list(time="19h", LPS="no", conc=all.conc.levels[-1], Fish=a.Fish)
tests$"(07)" <- mycontrast(mod, a, b)

## 8) compound effect (19h, yesLPS)?
cat("\n2.8 ---\n")
a <- list(time="19h", LPS="yes", conc="0", Fish=a.Fish)
b <- list(time="19h", LPS="yes", conc=all.conc.levels[-1], Fish=a.Fish)
tests$"(08)" <- mycontrast(mod, a, b)

## 9) overall effect, +/- LPS (3h)
cat("\n2.9 --- (averaged)\n")
## effect averaged over all 'conc' levels (using equal weights)
a <- list(time="3h", LPS="no", conc=all.conc.levels, Fish=a.Fish)
```

```
        b <- list(time="3h", LPS="yes", conc=all.conc.levels, Fish=a.Fish)
        tests$"(09)" <- mycontrast(mod, a, b, type="average")

        ## 10) overall effect, +/- LPS (19h)
        cat("\n2.10 --- (averaged)\n")
        ## effect averaged over all 'conc' levels
        a <- list(time="19h", LPS="no", conc=all.conc.levels, Fish=a.Fish)
        b <- list(time="19h", LPS="yes", conc=all.conc.levels, Fish=a.Fish)
        tests$"(10)" <- mycontrast(mod, a, b, type="average")

        ## 11) overall effect, 3/19h (noLPS)
        cat("\n2.11 --- (averaged)\n")
        ## effect averaged over all 'conc' levels
        a <- list(time="3h", LPS="no", conc=all.conc.levels, Fish=a.Fish)
        b <- list(time="19h", LPS="no", conc=all.conc.levels, Fish=a.Fish)
        tests$"(11)" <- mycontrast(mod, a, b, type="average")

        ## 12) overall effect, 3/19h (yesLPS)
        cat("\n2.12 --- (averaged)\n")
        ## effect averaged over all 'conc' levels
        a <- list(time="3h", LPS="yes", conc=all.conc.levels, Fish=a.Fish)
        b <- list(time="19h", LPS="yes", conc=all.conc.levels, Fish=a.Fish)
        tests$"(12)" <- mycontrast(mod, a, b, type="average")

        models[[assay]]$tests <- tests
    }
    if(write.textfile){
        sink(NULL)
    }

    ## --- save data and results for further analysis
    save(models, dat, file=paste0("results/" , compound, "_models.Rdata"))
  }


library(ggplot2)
library(contrast)
library(dplyr)

##  boxplots
cairo_pdf(paste0("plots/boxplots.pdf"), width=7, height=5, onefile=TRUE)

# loop over all compounds (for creating the boxplots)
for(compound in all.compounds) {
    print(paste("-- Compound:", compound))

    ## 1) load fitted models and data
    load(paste0("results/" , compound, "_models.Rdata"), verbose=T)
    ## rename PhagoG > Phago
    colnames(dat)[c(10,14)] <- c("Phago", "Phago.included")

    ## 2) compute 2^(-ddCt) for the genes
    ## per fish and per time point...
    dat <- dat %>% group_by(Fish, time) %>%
        mutate(IL10.ratio=2^(- IL10.dCt + mean(IL10.dCt[conc==0 & LPS=="no"], na.rm=T)),
               TNFa.ratio=2^(- TNFa.dCt + mean(TNFa.dCt[conc==0 & LPS=="no"], na.rm=T)),
               IL1B.ratio=2^(- IL1B.dCt + mean(IL1B.dCt[conc==0 & LPS=="no"], na.rm=T))) %>%
        ungroup()

    ## 3) plot results for substances
    for(assay in c("NBT", "Phago", "IL10.ratio", "TNFa.ratio", "IL1B.ratio")) {
        mod <- models[[assay]]$model
        select <- models[[assay]]$select
        ## Select a fish. It doesn't matter which one,
        ## because we don't have interactions with fish.
        a.Fish <- levels(droplevels(dat$Fish[select]))[1]
        ## select all conc levels
        all.conc.levels <- levels(droplevels(dat$conc[select]))
        n.conc.levels <- table(droplevels(dat$conc[select]))
        ## define text for x/ylab
```

```
        xtext <- switch(compound,
                        EG = "concentration [mM]",
                        But = "concentration [mM]",
                        "concentration [\uB5M]")          #all others
        ytext <- switch(assay,
                        NBT = "optical density",
                        Phago = "phagocytically active cells [%]",
                        "fold change as 2^(-ddCt)")          #all others

        ## boxplot for publication (boxplot for each assay and compound, separated by time)
        p3 <- ggplot(dat, aes_string(x="conc", y = assay, fill="LPS")) +
            scale_fill_manual(values=c ("gray60", "lightgoldenrod1")) +
            geom_boxplot(size= 0.2, outlier.colour = "black", outlier.size = 0.5) +
            facet_wrap(~ time) +
            xlab(sprintf(xtext)) +
            ylab(ytext) +
            ggtitle(compound, assay) +
            theme(panel.background = element_rect(fill = 'gray98', colour = 'gray98'),
                  panel.grid.major = element_line(colour = "gray80", size = 0.2),
                  panel.grid.minor = element_line(colour = "gray90", size = 0.2),
                  panel.spacing.x = unit(5, "mm"),
                  strip.background = element_rect(fill = 'gray90'))
        ## theme(axis.text.x = element_text(angle = 60, hjust = 1))
        print(p3)
    }
 }

## 3) plots to summarize test
## to extract the contrasts and p-values
tests.df <- data.frame(compound=character(), assay=character(), test=character(),
                       Pvalue=double(), contrast=double(), stat=double(), maxlevel=logical())
for(compound in c("BaP", "BPA", "But", "DCF", "Dex", "EE2", "EG", "TCB")) {
    load(paste0("results/" , compound, "_models.Rdata"))
    for(assay in names(models)) {
        for(t in names(models[[assay]]$tests)) {
            n.contrasts <- length(models[[assay]]$tests[[t]]$Pvalue)
            for(i in 1:n.contrasts){
                tests.df <- rbind(tests.df,
                                  data.frame(compound=compound,
                                             assay=assay,
                                             test=paste0(t, ".", i),
                                             Pvalue=models[[assay]]$tests[[t]]$Pvalue[i],
                                             contrast=models[[assay]]$tests[[t]]$Contrast[i],
                                             stat=models[[assay]]$tests[[t]]$testStat[i],
                                             stringsAsFactors=F,
                                             maxlevel= i == n.contrasts))
            }
        }
    }
}
## separate time
tests.df$time <- NA
tests.df$time[grepl("03h_", tests.df$test)] <- "3h"
tests.df$time[grepl("19h_", tests.df$test)] <- "19h"
tests.df$time <- factor(tests.df$time, levels = c("3h", "19h"))
## clean up test names
m <- regexpr("[0-9]+", tests.df$test)
n1 <- regmatches(tests.df$test, m)
m <- regexpr("\\.[1-9]", tests.df$test)
n2 <- substring(regmatches(tests.df$test, m), 2, 2)
tests.df$testnumber <- as.factor(paste0(n1, "-", n2))
tests.df$testnumber2 <- as.factor(paste0(n1))
tests.df$testname <- gsub("\\([0-9]+\\)", "", tests.df$test)
tests.df$testname.notime <- gsub("\\([0-9]+\\)|03h_|19h_", "", tests.df$test)
## reorder compounds
tests.df$compound <- factor(tests.df$compound, levels = rev(c("Dex", "DCF", "BaP", "BisA",
                                                              "EE2", "TCB", "EG", "But")))
## reorder assays
tests.df$assay <- factor(tests.df$assay, levels = c("PhagoG", "NBT", "IL1B.dCt",
                                                    "TNFa.dCt", "IL10.dCt"))
```

```
tests.df$assay <- recode_factor(tests.df$assay, "PhagoG"="Phago")
## add P-value with effect sign. for color scale
tests.df$Pvalue.sign <- sign(tests.df$contrast) * (1 - tests.df$Pvalue)
## and for NBT and phago
tests.df$Pvalue.sign[tests.df$assay %in% c("NBT", "Phago")] <- -1*tests.df$Pvalue.sign[tests.df$assay %in%
c("NBT", "Phago")]
## rename tests
tests.df$testnumber <- recode_factor(tests.df$testnumber,
                                     "01-1" = "01_03h_control with LPS",
                                     "02-1"="02_19h_control with LPS",
                                     "03-1" = "03_time differences without LPS",
                                     "04-1" = "04_time differences with LPS",
                                     "05-1" = "05_03h_test compound_1", "05-2" = "05_03h_test compound_2",
"05-3" = "05_03h_test compound_3", "05-4" = "05_03h_test compound_4",
                                     "06-1" = "06_03h_test compound with LPS_1", "06-2" = "06_03h_test
compound with LPS_2", "06-3" = "06_03h_test compound with LPS_3", "06-4" = "06_03h_test compound with
LPS_4",
                                     "07-1" = "07_19h_test compound_1", "07-2" = "07_19h_test compound_2",
"07-3" = "07_19h_test compound_3", "07-4" = "07_19h_test compound_4",
                                     "08-1" = "08_19h_test compound with LPS_1", "08-2" = "08_19h_test
compound with LPS_2", "08-3" = "08_19h_test compound with LPS_3", "08-4" = "08_19h_test compound with
LPS_4",
                                     "09-1" = "09_overall LPS differences at 3h",
                                     "10-1" = "10_overall LPS differences at 19h",
                                     "11-1" = "11_overall time differences without LPS",
                                     "12-1" = "12_overall time differences with LPS")

## 3.1) p-values and test statistics
tests.df.sub <- tests.df
pdf(paste0("plots/p-values.pdf"), width=8.5, height=10)

## p values
p <- ggplot(tests.df.sub, aes(testnumber, compound)) +
    geom_tile(aes(fill = cut(Pvalue, c(0, 0.001, 0.01, 0.05, 1), include.lowest = T))) +
    geom_text(aes(label=round(Pvalue,1)), size=2) +
    geom_abline(slope=0, intercept=c(3.5,7.5)) +
    geom_vline(xintercept=c(2.5, 4.5, 8.5, 12.5, 16.5, 20.5, 22.5), colour = "dark gray") +
    scale_fill_brewer(type="seq", palette = "BuGn", direction=-1) +
    facet_grid(assay ~ .) +
    coord_equal() +
    labs(x = "") +
    guides(fill=guide_legend(title="p-values")) +
    theme_minimal() +
    theme(axis.text.x = element_text(angle = 90, vjust = 0.5, hjust=1),
          panel.grid.major = element_blank(), panel.grid.minor = element_blank(),
          panel.spacing.y = unit(4, "mm"),  # distance between the assays
          ## panel.background = element_rect(fill = 'white', colour = 'gray90'),
          strip.background = element_rect(fill = 'gray90', colour = 'gray90'))
print(p)
pdf(paste0("plots/test-stat.pdf"), width=8.5, height=10)

## --- test statistics
p <- ggplot(tests.df.sub, aes(x=testnumber, y=compound)) +
    geom_tile(aes(fill = factor(cut(Pvalue.sign, c(-55, 0, 55), include.lowest = T),
                                labels=c("suppressed", "stimulated")))) +
    geom_text(aes(label=round(stat,1)), size=1.8) +
    facet_grid(assay ~ .) +
    coord_equal() +
    geom_abline(slope=0, intercept=c(3.5,7.5))+
    geom_vline(xintercept=c(2.5, 4.5, 8.5, 12.5, 16.5, 20.5, 22.5), colour = "dark gray") +
    guides(fill=guide_legend(title="immune parameters")) +
    xlab("") +
    theme_minimal() +
    ## theme(axis.text.y = element_text(size=18)) +
    theme(axis.text.x = element_text(angle = 90, vjust = 0.5, hjust=1),
          panel.grid.major = element_blank(), panel.grid.minor = element_blank(),
          panel.spacing.y = unit(4, "mm"),  # distance between the assays
          ## panel.background = element_rect(fill = 'white', colour = 'gray90'),
          strip.background = element_rect(fill = 'gray90', colour = 'gray90')
          )
```

```
print(p)
dev.off()

## 3.2) heat map for results section
## subset the relevant tests and only the ones with the higherst level
tests.df.sub <- tests.df %>%
    filter(testnumber2 %in% c("01", "02", "05", "07", "06", "08"), maxlevel) %>%
    droplevels
## create a new factor which combines the same tests of different time points
tests.df.sub$testnumber3 <- tests.df.sub$testnumber2
levels(tests.df.sub$testnumber3) <- list("control with LPS"=c("01", "02"), "test compound"=c("05", "07"),
"test compound with LPS"=c("06", "08"))
pdf(paste0("plots/heat-map.pdf"), width=8.5, height=10)
## --- publication plot
p <- ggplot(tests.df.sub, aes(x=testnumber3, y=compound)) +
    geom_tile(aes(fill = cut(Pvalue.sign, c(-1, -0.999, -0.99, -0.95, 0,
                                            0.95, 0.99, 0.999, 1),
                             include.lowest = T))) +
    ## geom_text(aes(label=round(stat,2)), size=2) +
    scale_fill_brewer(type="seq", palette = "RdYlBu",
                      labels=c("- [0 - 0.001]", "- [0.001 - 0.01]", "- [0.01 - 0.05]",
                               "- [0.05 - 1]", "+ [1 - 0.05]", "+ [0.05 - 0.01]",
                               "+ [0.01 - 0.001]", "+ [0.001 - 0]")) +
    facet_grid(time ~ assay) +
        coord_equal() +
        geom_abline(slope=0, intercept=c(3.5,7.5))+
        guides(fill=guide_legend(title="p-values")) +
        xlab("") +
        theme_minimal() +
    theme(axis.text.x = element_text(angle = 90, vjust = 0.5, hjust=1),
          ## axis.text.y = element_text(size=18),
          panel.grid.major = element_blank(), panel.grid.minor = element_blank(),
          panel.spacing.x = unit(4, "mm"),
          panel.spacing.y = unit(7, "mm"),
          strip.text = element_text(size=12)
          ## panel.background = element_rect(fill = 'white', colour = 'gray90'),
          ## strip.background = element_rect(fill = 'gray90', colour = 'gray90')
          )
print(p)
dev.off()


## model diagnosis
pdf(paste0("results/" , compound, "_diagnostic-plots.pdf"), width=7, height=5)
ar(mfrow=c(2,2))
plot(mod)
```

S4: Standard deviation and fish n-number

Calculated standard deviation for technical errors applied in step "select valid data" of the R-model for the respiratory burst (NBT) and phagocytosis (PhagoG) activity are listed.
For each immune parameter, the selected fish for statistical analysis and the corresponding n-number are shown.
Test chemicals in alphabetic order.

Benzo(a)pyrene

standard deviation technical errors:	NBT	PhagoG
	0.0187	1.218

Selected for NBT :	Fish B Fish C Fish D Fish E	n=4
Selected for PhagoG :	Fish A Fish B Fish C Fish D Fish E	n=5
Selected for IL10.dCt :	Fish A Fish B Fish C Fish D Fish E	n=5
Selected for TNFa.dCt :	Fish A Fish B Fish C Fish D Fish E	n=5
Selected for IL1B.dCt :	Fish A Fish B Fish C Fish D Fish E	n=5

Bisphenol A

standard deviation technical errors:	NBT	PhagoG
	0.0122	0.7029

Selected for NBT :	Fish A Fish C Fish F	n=3
Selected for PhagoG :	Fish C Fish E Fish F	n=3
Selected for IL10.dCt :	Fish A Fish B Fish D Fish F	n=4
Selected for TNFa.dCt :	Fish A Fish B Fish C Fish D Fish E Fish F	n=6
Selected for IL1B.dCt :	Fish A Fish B Fish C Fish D Fish E Fish F	n=6

Butanol

standard deviation technical errors:	NBT	PhagoG
	0.0105	0.6863

Selected for NBT :	Fish B Fish D Fish E Fish F	n=4
Selected for PhagoG :	Fish A Fish C Fish E Fish F	n=4
Selected for IL10.dCt :	Fish A Fish B Fish C Fish D Fish E Fish F	n=6
Selected for TNFa.dCt :	Fish A Fish B Fish C Fish F	n=4
Selected for IL1B.dCt :	Fish A Fish B Fish C Fish D Fish E Fish F	n=6

Diclofenac

standard deviation technical errors:	NBT	PhagoG
	0.009	0.8964

Selected for NBT :	Fish A Fish B Fish D Fish F	n=4
Selected for PhagoG :	Fish A Fish B Fish C Fish D Fish F Fish G	n=6
Selected for IL10.dCt :	Fish A Fish B Fish D Fish E	n=4
Selected for TNFa.dCt :	Fish A Fish B Fish C Fish E Fish G	n=5
Selected for IL1B.dCt :	Fish A Fish B Fish C Fish D Fish E Fish F Fish G	n=7

Dexamethasone

standard deviation technical errors:	NBT	PhagoG
	0.0093	0.6

Selected for NBT :	Fish B Fish C Fish D Fish E Fish F	n=5
Selected for PhagoG :	Fish A Fish C Fish E Fish F	n=4
Selected for IL10.dCt :	Fish A Fish B Fish C Fish D Fish E Fish F	n=6
Selected for TNFa.dCt :	Fish A Fish B Fish C Fish E Fish F	n=5
Selected for IL1B.dCt :	Fish A Fish B Fish C Fish D Fish E Fish F	n=6

Ethinylestradiol

standard deviation technical errors:	NBT	PhagoG
	0.0178	0.8022

Selected for NBT :	Fish A Fish B Fish D Fish E	n=4
Selected for PhagoG :	Fish A Fish B Fish E	n=3
Selected for IL10.dCt :	Fish A Fish B Fish E Fish F	n=4
Selected for TNFa.dCt :	Fish A Fish B Fish C Fish D Fish E Fish F	n=6
Selected for IL1B.dCt :	Fish A Fish B Fish C Fish D Fish E Fish F	n=6

Ethylene glycol

standard deviation technical errors:	NBT	PhagoG
	0.0151	0.5169

Selected for NBT :	Fish A Fish B Fish C Fish E Fish F	n=5
Selected for PhagoG :	Fish A Fish B Fish C Fish D Fish E Fish F	n=6
Selected for IL10.dCt :	Fish A Fish B Fish D Fish E Fish F	n=5
Selected for TNFa.dCt :	Fish A Fish B Fish C Fish E Fish F	n=5
Selected for IL1B.dCt :	Fish A Fish B Fish C Fish D Fish E Fish F	n=6

Trichlorobenzene

standard deviation technical errors:	NBT	PhagoG
	0.0132	0.5518

Selected for NBT :	Fish D Fish E Fish F	n=3
Selected for PhagoG :	Fish A Fish C Fish D Fish E Fish F	n=5
Selected for IL10.dCt :	Fish A Fish B Fish C Fish D Fish E Fish F	n=6
Selected for TNFa.dCt :	Fish A Fish B Fish C Fish D Fish E Fish F	n=6
Selected for IL1B.dCt :	Fish A Fish B Fish C Fish D Fish E Fish F	n=6

S5: Diagnostic plots

Test chemicals in alphabetic order

Benzo(a)pyrene

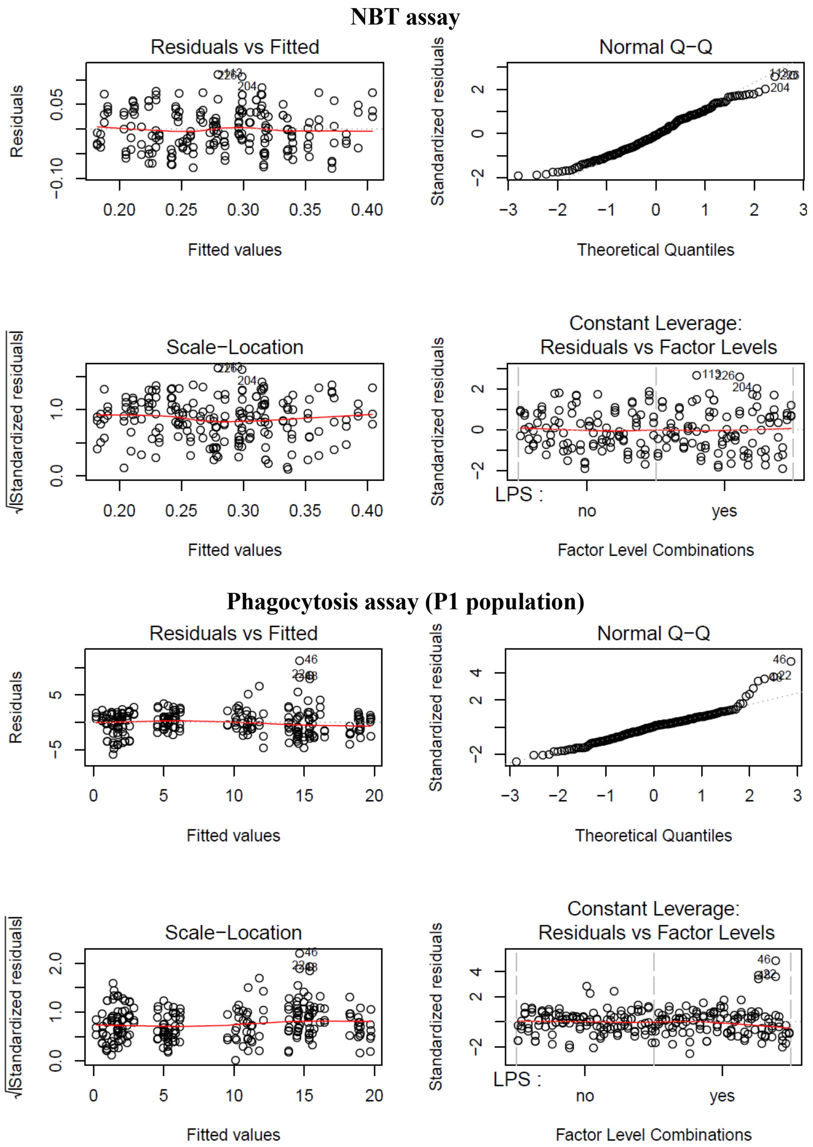

Fig. S1a Diagnostic plots for Benzo(a)pyrene

***IL-10* transcription (delta Ct values)**

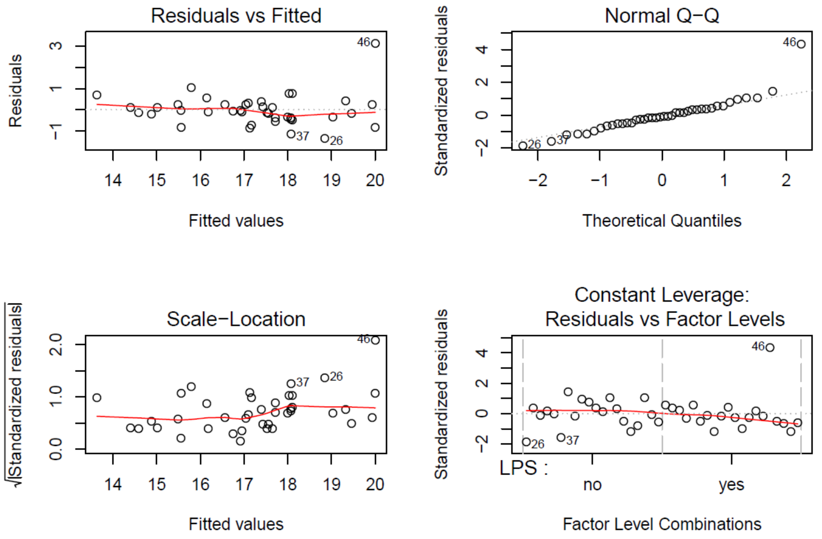

***TNFα* transcription (delta Ct values)**

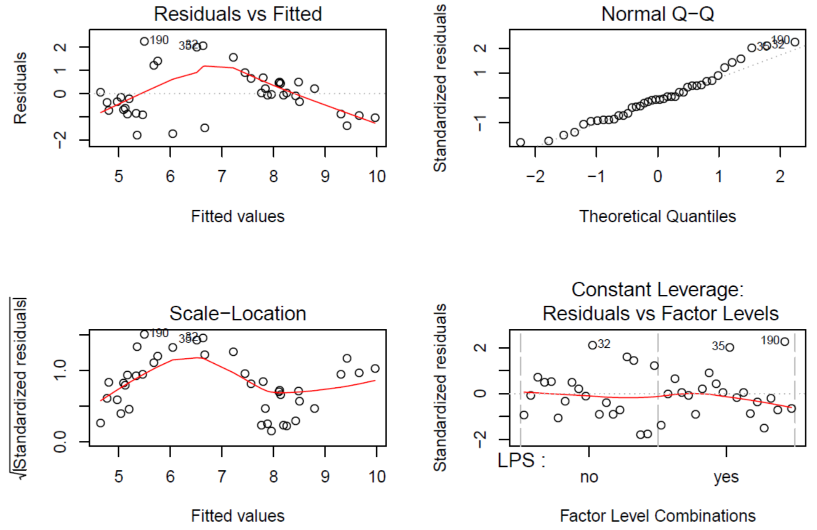

***IL-1β* transcription (delta Ct values)**

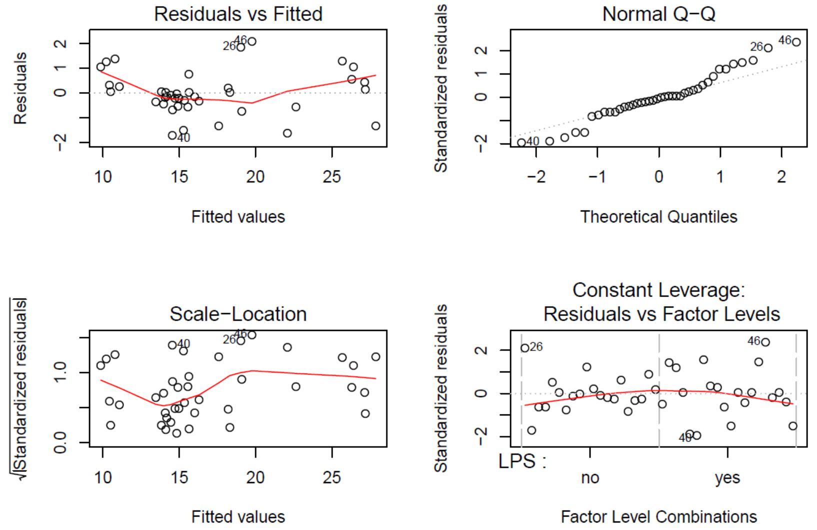

Fig. S1b Diagnostic plots for Benzo(a)pyrene

Bisphenol A

NBT assay

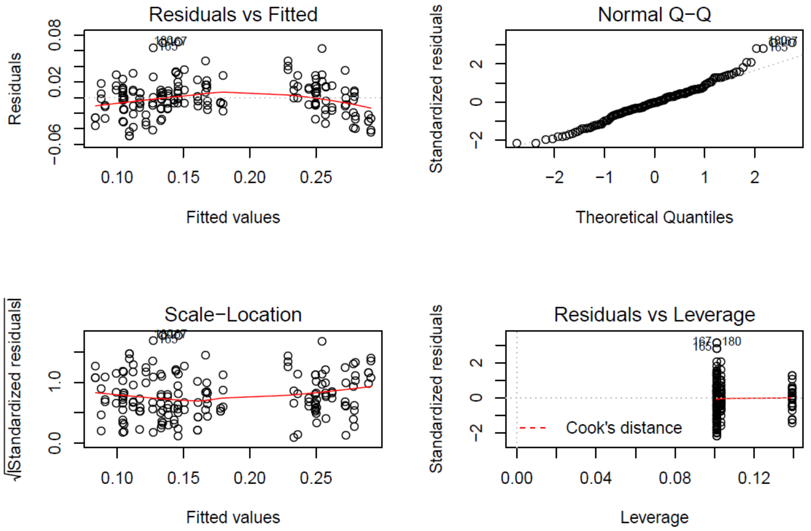

Phagocytosis assay (P1 population)

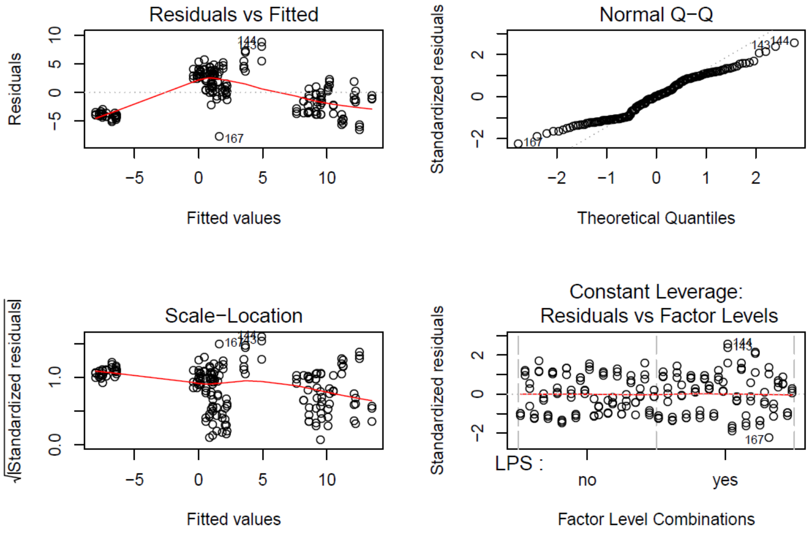

***IL-10* transcription (delta Ct values)**

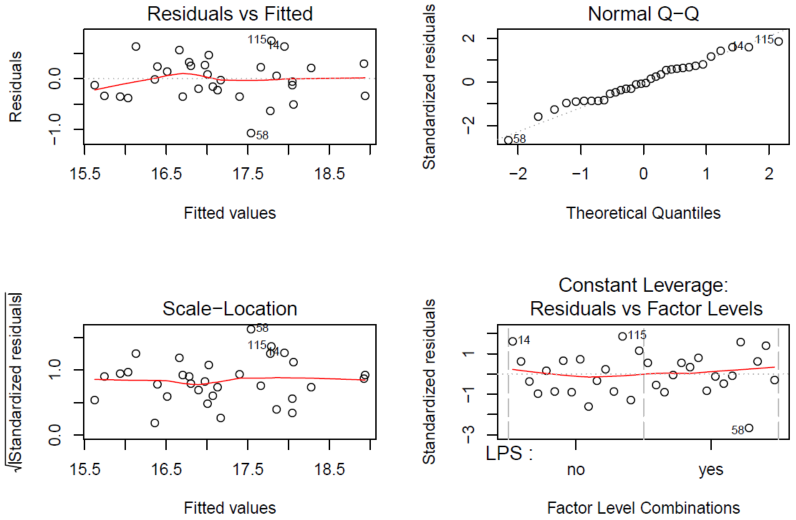

Fig. S2a Diagnostic plots for Bisphenol A

***TNFα* transcription (delta Ct values)**

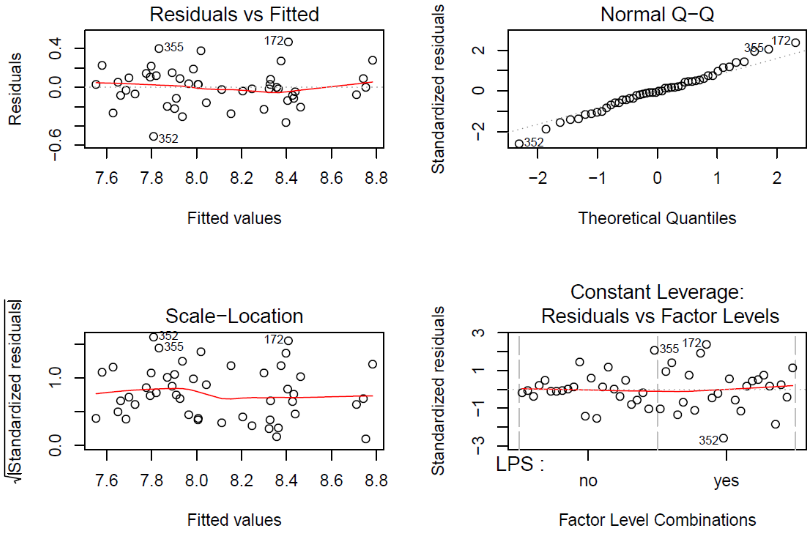

***IL-1β* transcription (delta Ct values)**

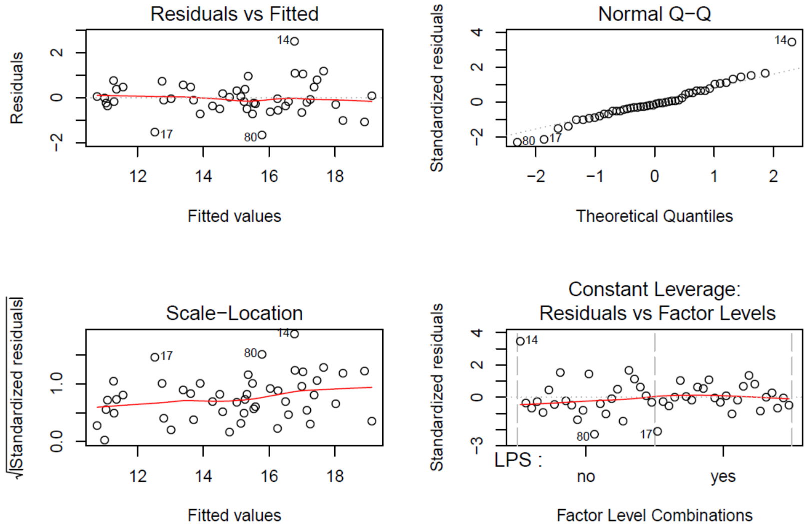

Fig. S2b Diagnostic plots for Bisphenol A

Butanol

NBT assay

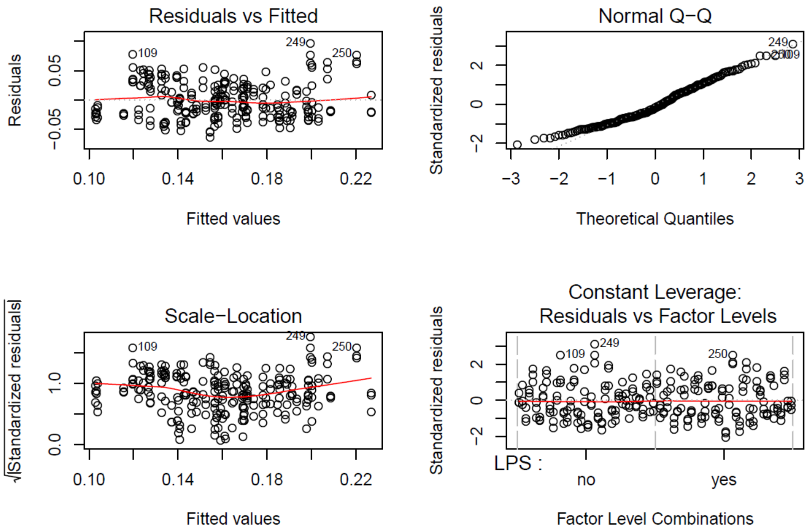

Phagocytosis assay (P1 population)

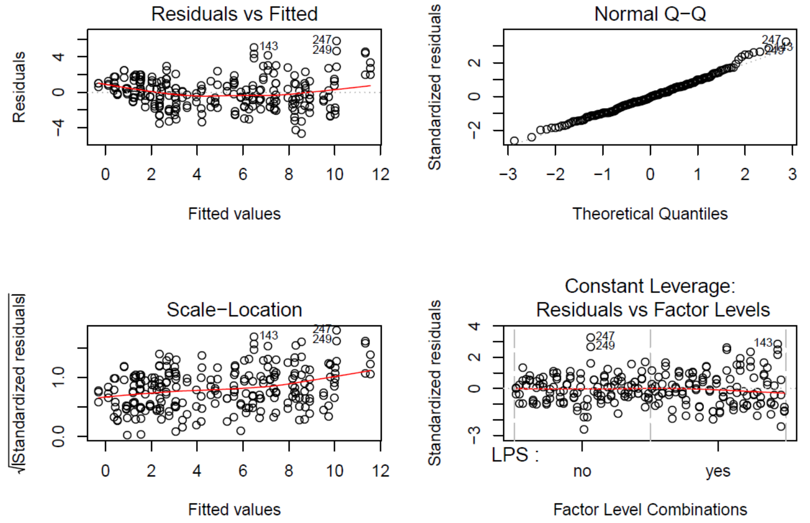

IL-10 transcription (delta Ct values)

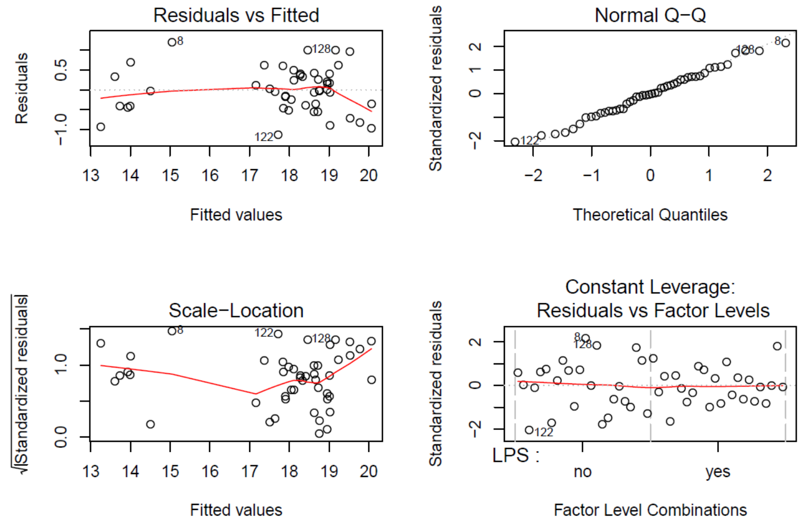

Fig. S3a Diagnostic plots for Butanol

***TNFα* transcription (delta Ct values)**

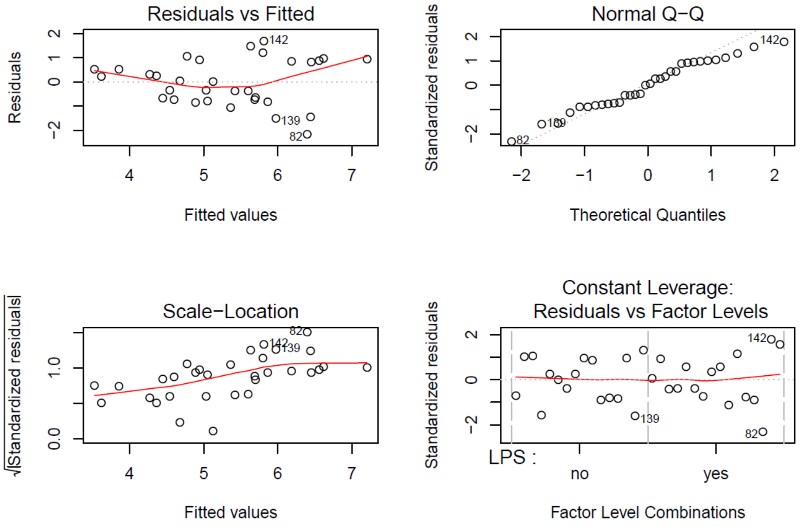

***IL-1β* transcription (delta Ct values)**

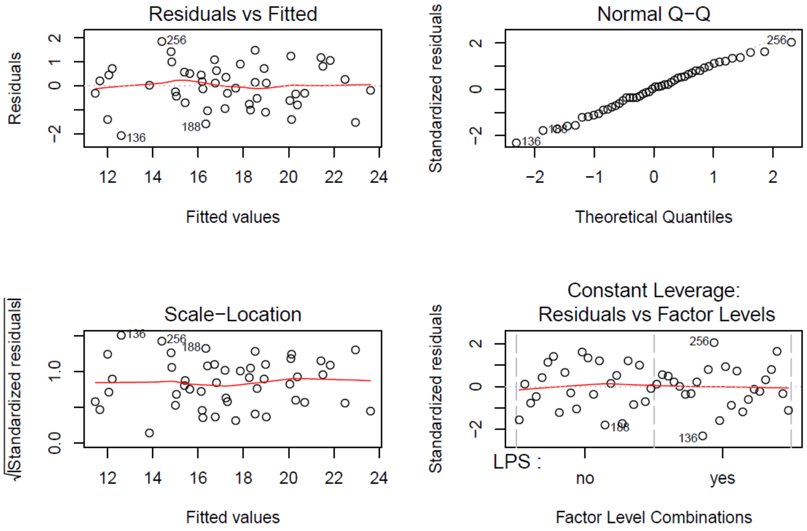

Fig. S3b Diagnostic plots for Butanol

Diclofenac

NBT assay

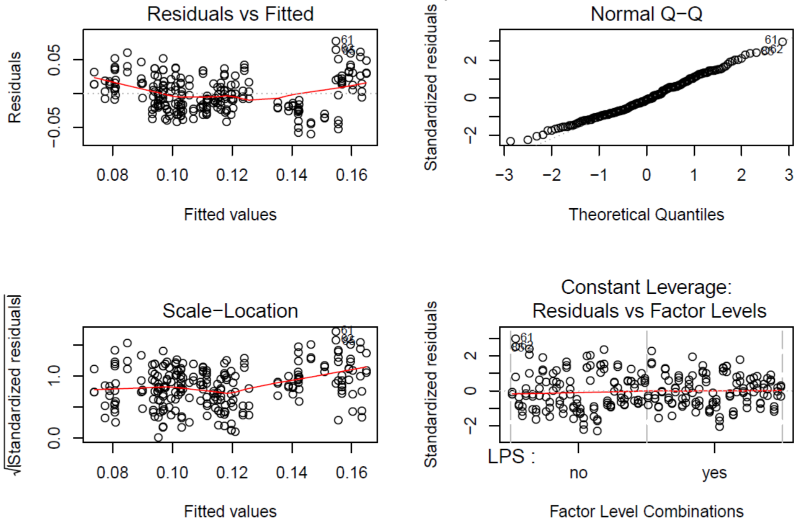

Phagocytosis assay (P1 population)

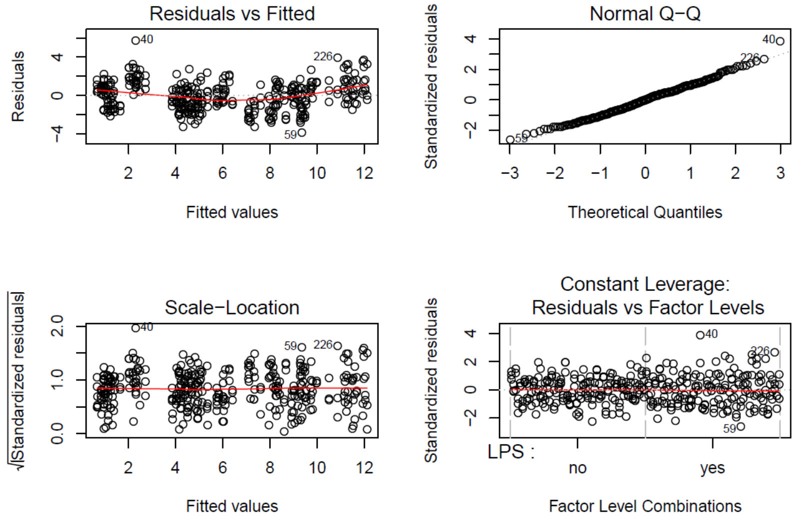

***IL-10* transcription (delta Ct values)**

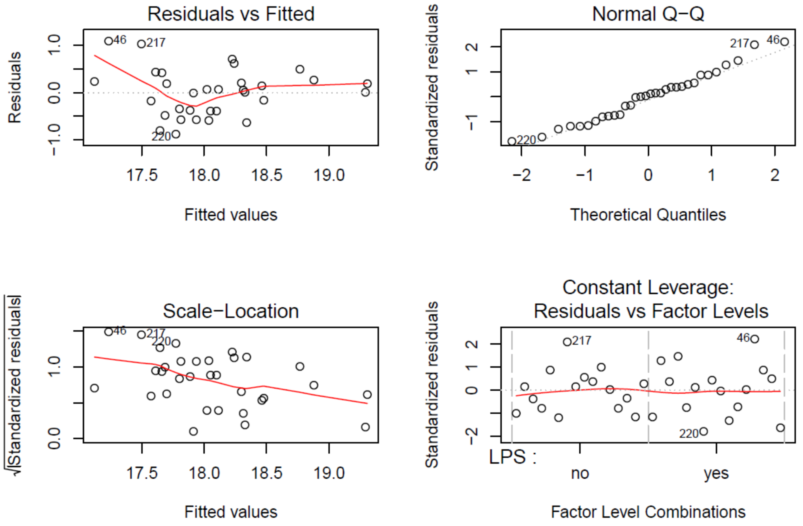

Fig. S4a Diagnostic plots for Diclofenac

***TNFα* transcription (delta Ct values)**

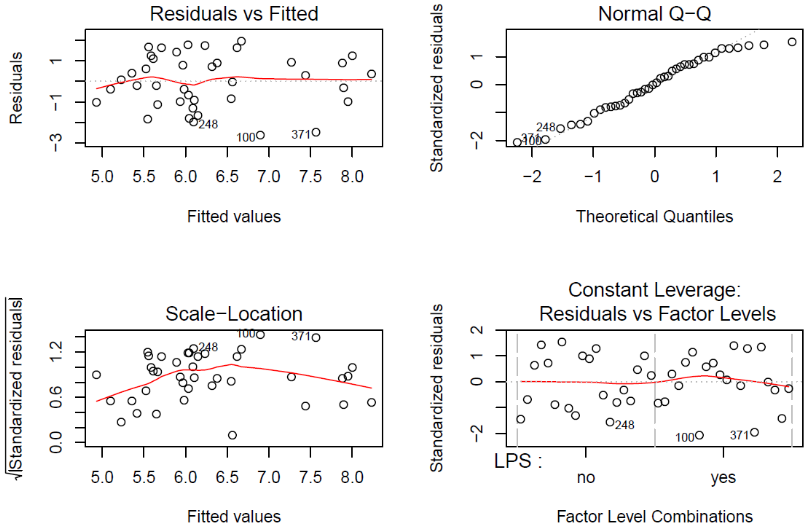

***IL-1β* transcription (delta Ct values)**

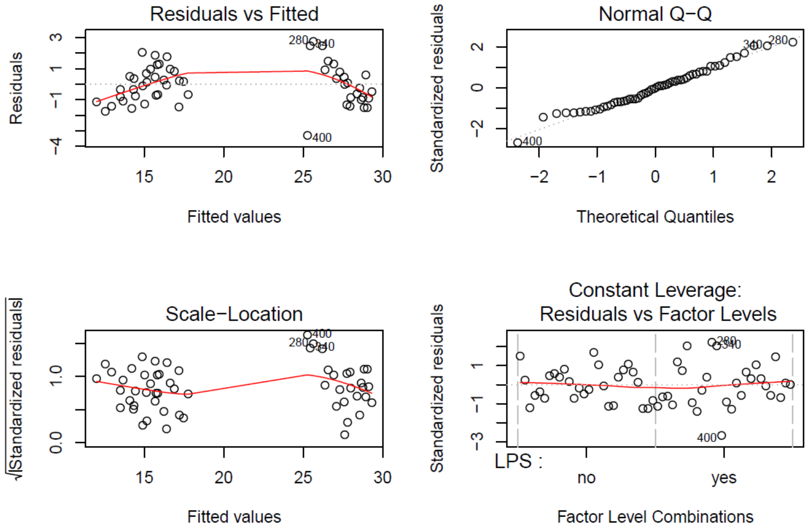

Fig. S4b Diagnostic plots for Diclofenac

Dexamethasone

NBT assay

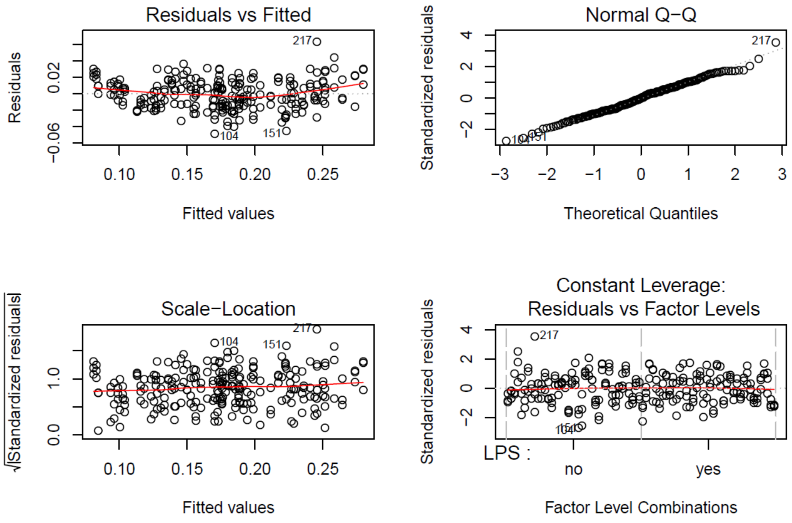

Phagocytosis assay (P1 population)

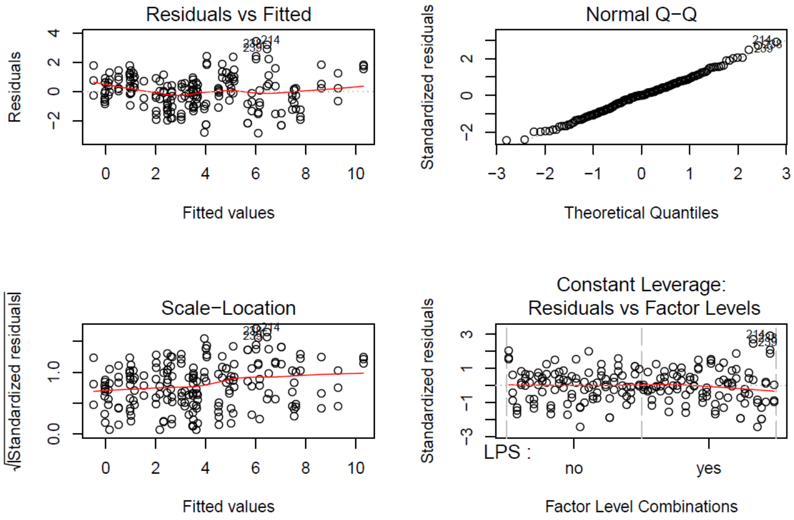

***IL-10* transcription (delta Ct values)**

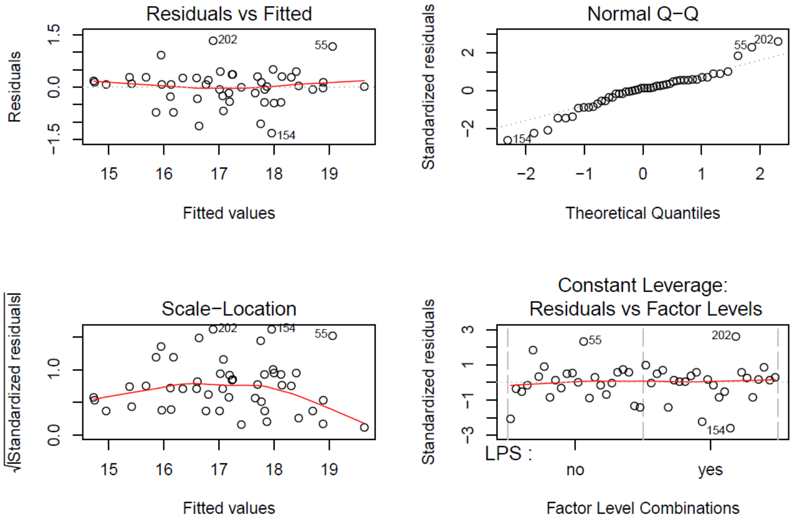

Fig. S5a Diagnostic plots for Dexamethasone

***TNFα* transcription (delta Ct values)**

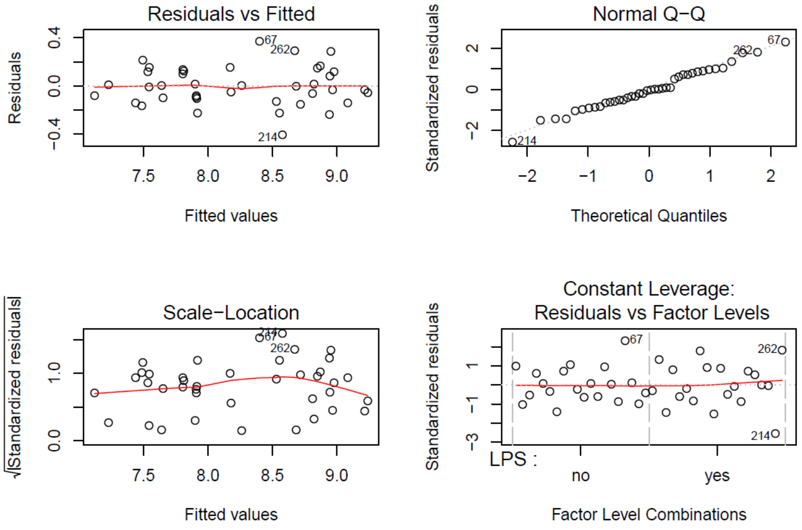

***IL-1β* transcription (delta Ct values)**

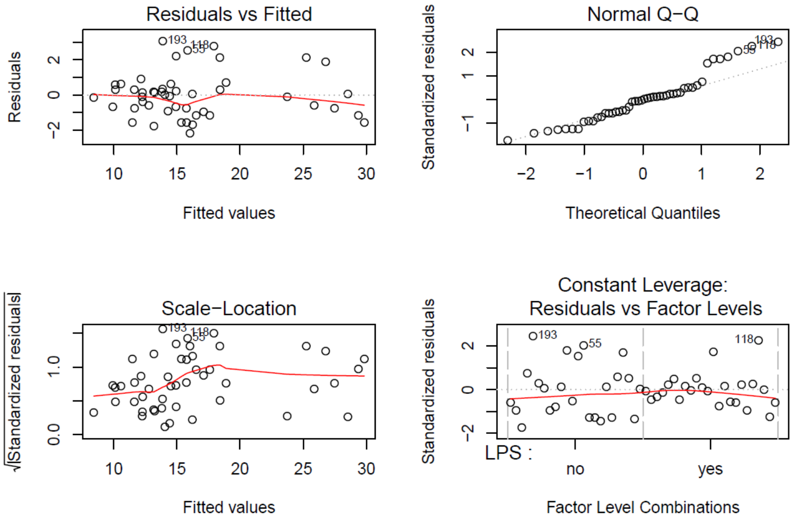

Fig. S5b Diagnostic plots for Dexamethasone

Ethinylestradiol

NBT assay

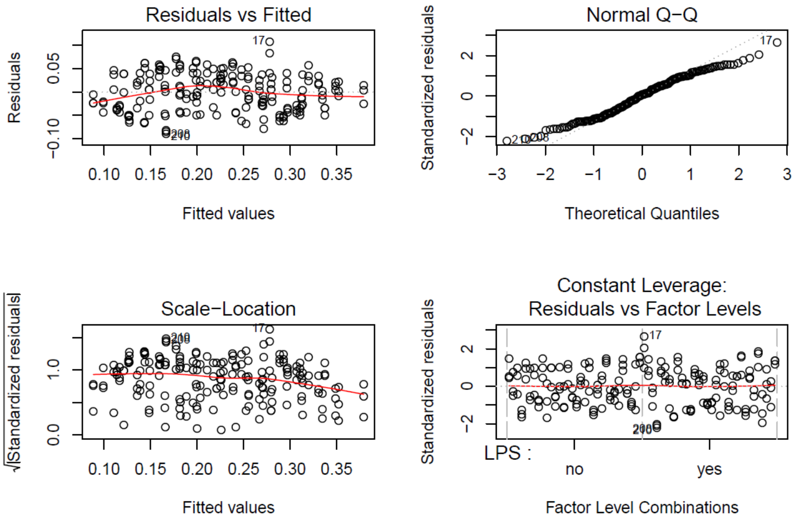

Phagocytosis assay (P1 population)

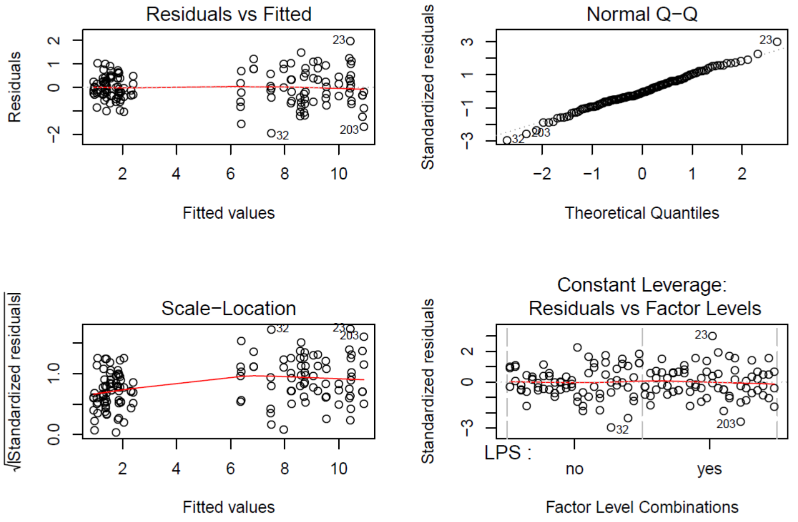

IL-10 transcription (delta Ct values)

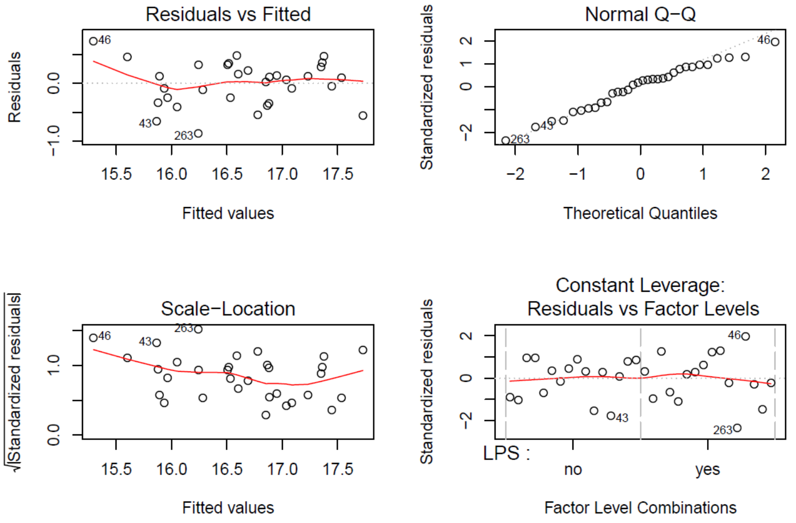

Fig. S6a Diagnostic plots for Ethinylestradiol

***TNFα* transcription (delta Ct values)**

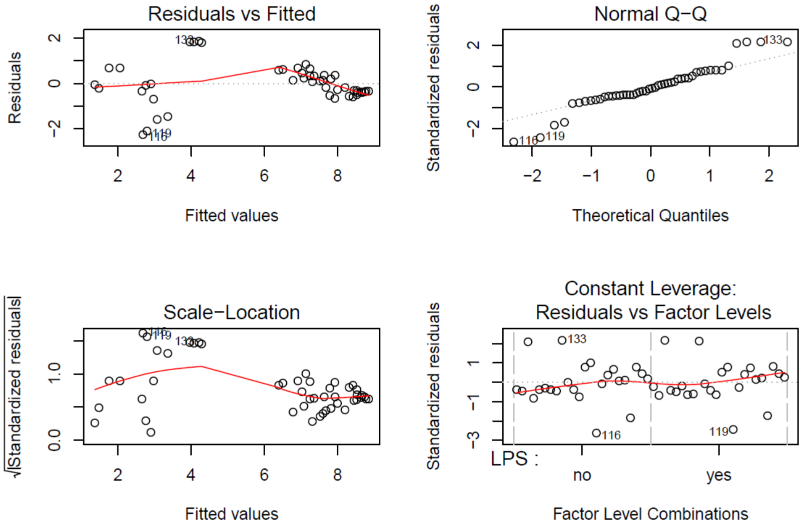

***IL-1β* transcription (delta Ct values)**

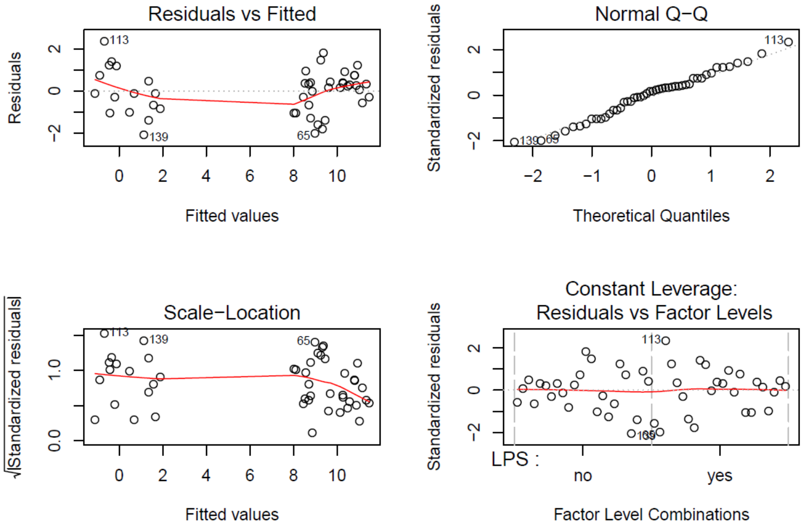

Fig. S6b Diagnostic plots for Ethinylestradiol

Ethylene glycol

NBT assay

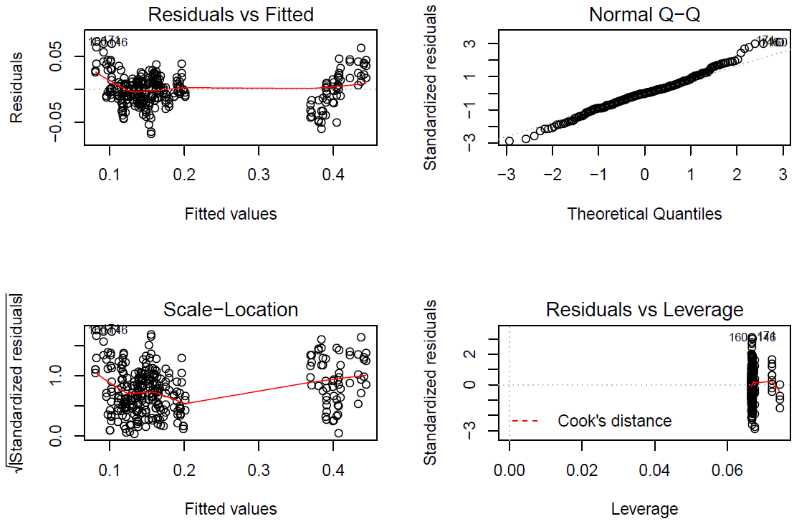

Phagocytosis assay (P1 population)

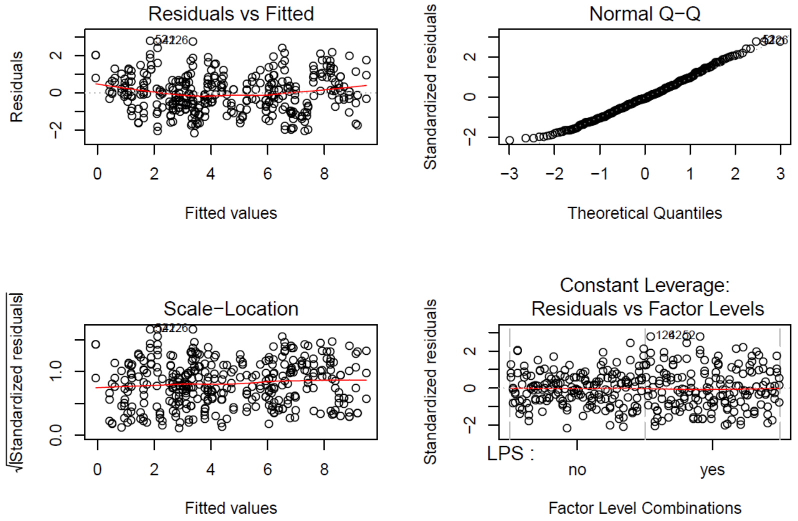

IL-10 transcription (delta Ct values)

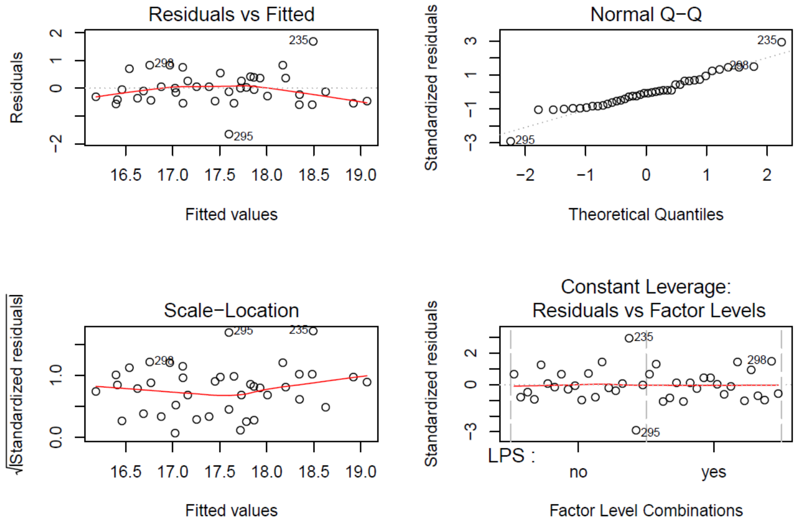

Fig. S7a Diagnostic plots for Ethylene glycol

***TNFα* transcription (delta Ct values)**

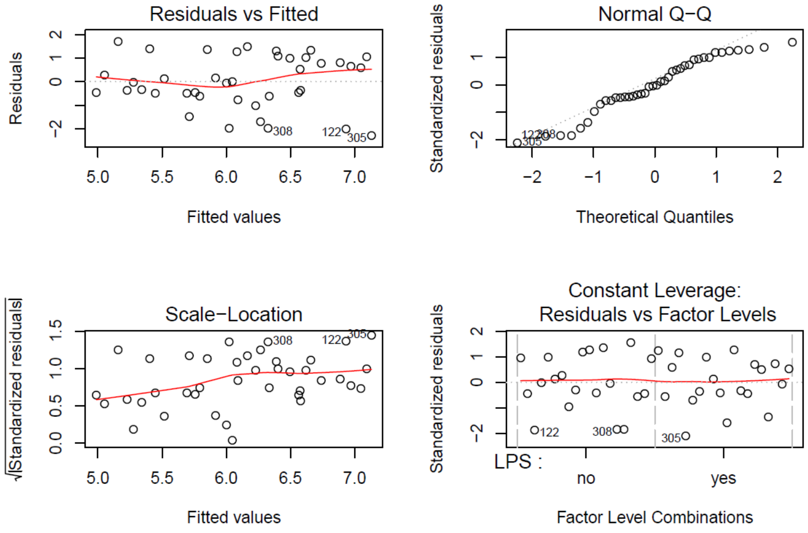

***IL-1β* transcription (delta Ct values)**

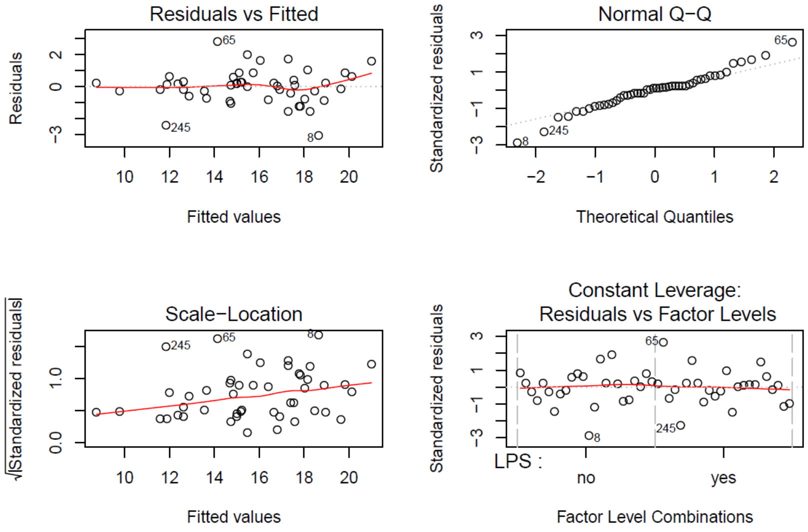

Fig. S7b Diagnostic plots for Ethylene glycol

Trichlorobenzene

NBT assay

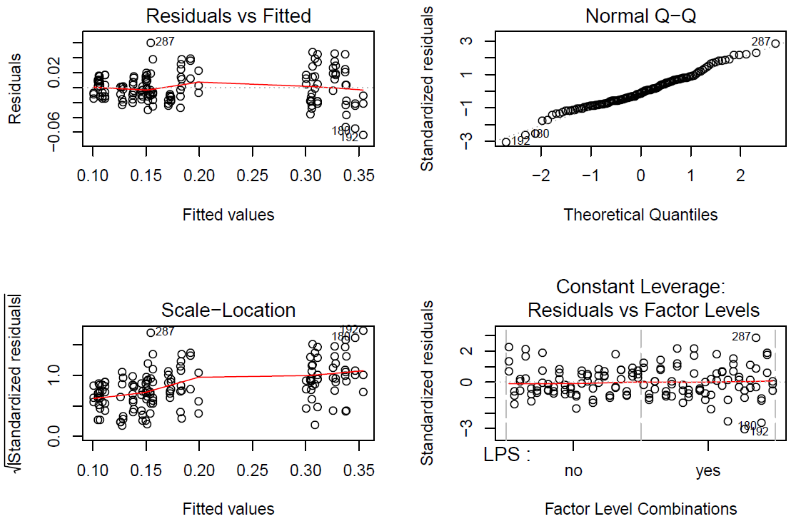

Phagocytosis assay (P1 population)

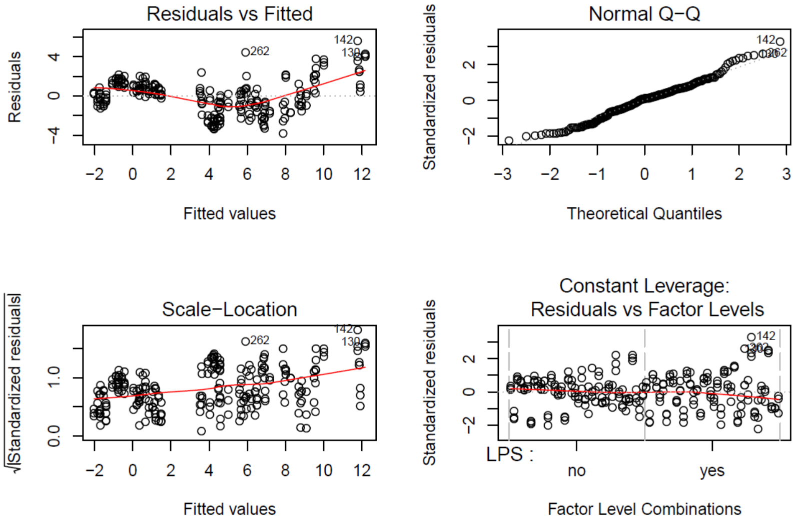

***IL-10* transcription (delta Ct values)**

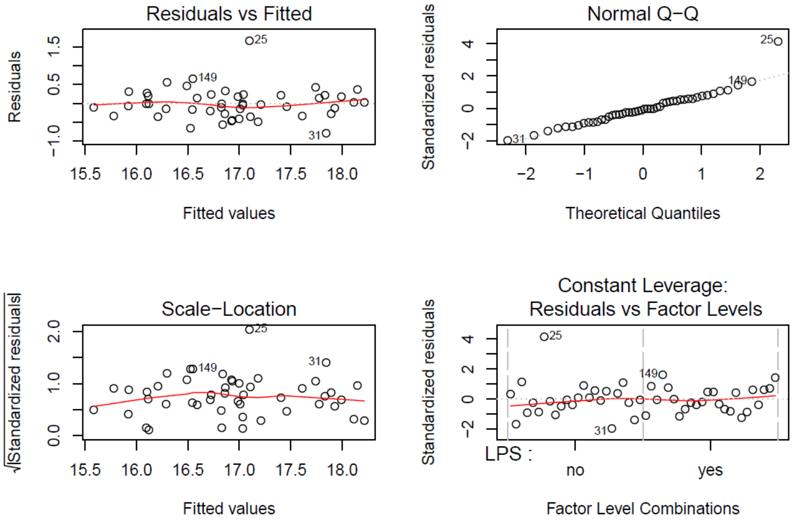

Fig. S8a Diagnostic plots for Trichlorobenzene

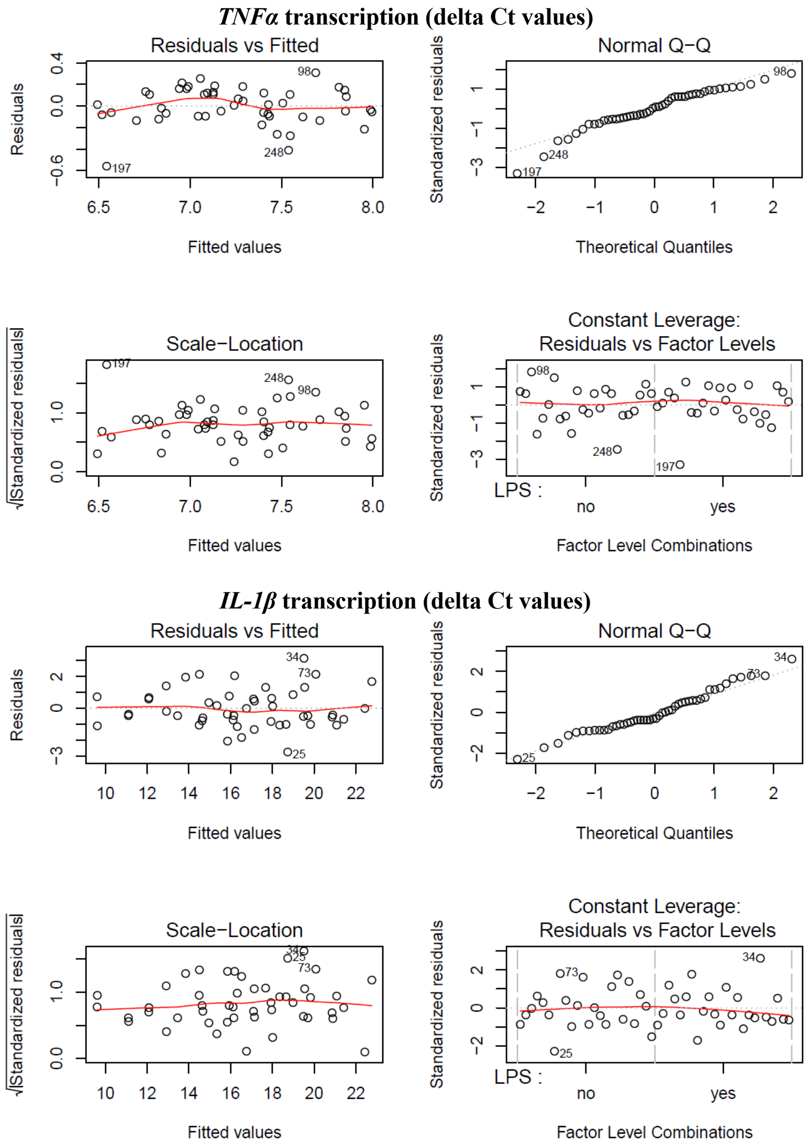

Fig. S8b Diagnostic plots for Trichlorobenzene

S6: Cytotoxicity assays to determine cell viability

Test chemicals in alphabetic order

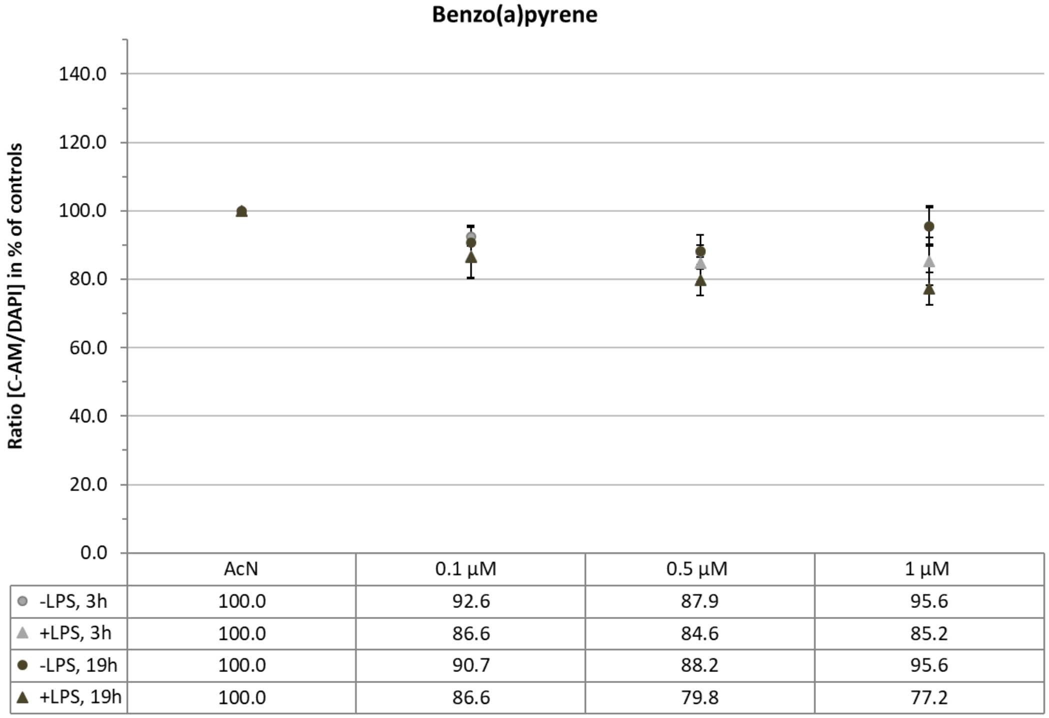

	AcN	0.1 µM	0.5 µM	1 µM
-LPS, 3h	100.0	92.6	87.9	95.6
+LPS, 3h	100.0	86.6	84.6	85.2
-LPS, 19h	100.0	90.7	88.2	95.6
+LPS, 19h	100.0	86.6	79.8	77.2

Fig. S9 Cytotoxicity assays to determine cell viability for different concentrations of Benzo(a)pyrene. Viability in percentage of control for short- (3 h, gray) and long-term (19 h, black) exposure, without (dot) or with (triangle) previous LPS stimulation.

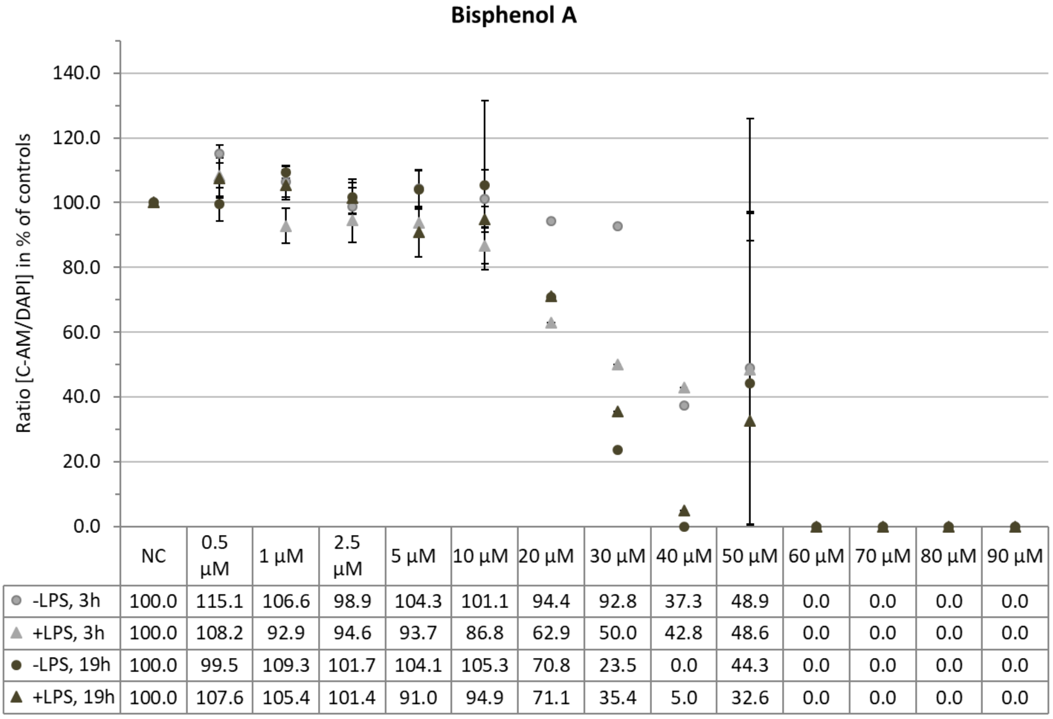

	NC	0.5 µM	1 µM	2.5 µM	5 µM	10 µM	20 µM	30 µM	40 µM	50 µM	60 µM	70 µM	80 µM	90 µM
-LPS, 3h	100.0	115.1	106.6	98.9	104.3	101.1	94.4	92.8	37.3	48.9	0.0	0.0	0.0	0.0
+LPS, 3h	100.0	108.2	92.9	94.6	93.7	86.8	62.9	50.0	42.8	48.6	0.0	0.0	0.0	0.0
-LPS, 19h	100.0	99.5	109.3	101.7	104.1	105.3	70.8	23.5	0.0	44.3	0.0	0.0	0.0	0.0
+LPS, 19h	100.0	107.6	105.4	101.4	91.0	94.9	71.1	35.4	5.0	32.6	0.0	0.0	0.0	0.0

Fig. S10 Cytotoxicity assays to determine cell viability for different concentrations of Bisphenol A. Viability in percentage of control for short- (3 h, gray) and long-term (19 h, black) exposure, without (dot) or with (triangle) previous LPS stimulation.

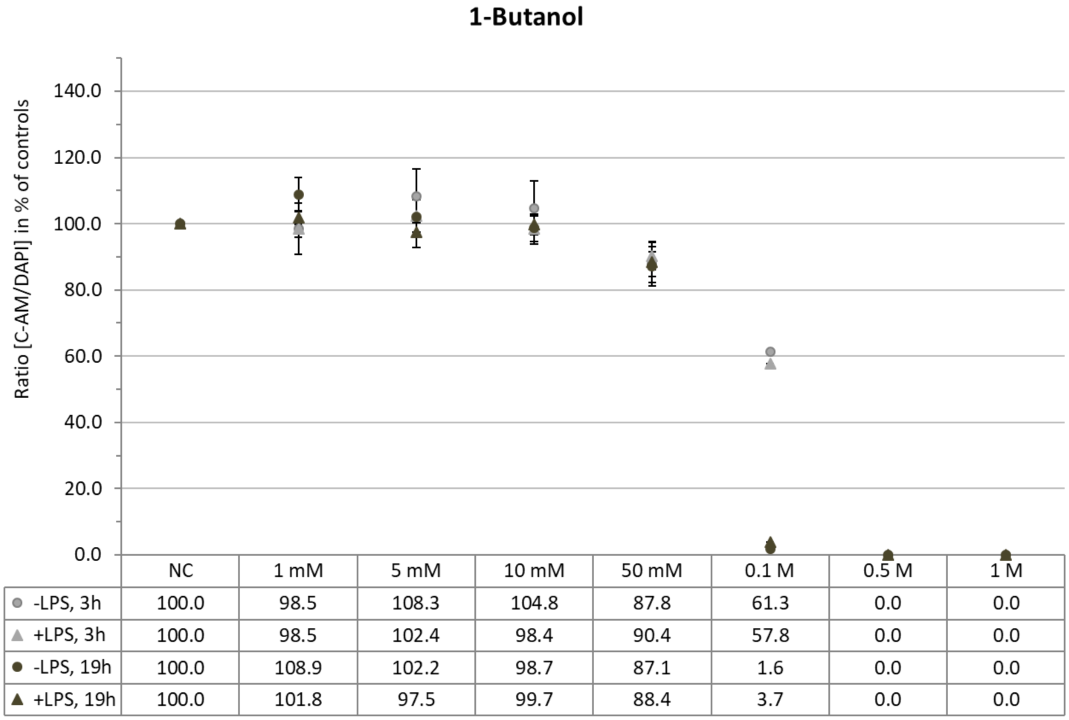

	NC	1 mM	5 mM	10 mM	50 mM	0.1 M	0.5 M	1 M
-LPS, 3h	100.0	98.5	108.3	104.8	87.8	61.3	0.0	0.0
+LPS, 3h	100.0	98.5	102.4	98.4	90.4	57.8	0.0	0.0
-LPS, 19h	100.0	108.9	102.2	98.7	87.1	1.6	0.0	0.0
+LPS, 19h	100.0	101.8	97.5	99.7	88.4	3.7	0.0	0.0

Fig. S11 Cytotoxicity assays to determine cell viability for different concentrations of Butanol. Viability in percentage of control for short- (3 h, gray) and long-term (19 h, black) exposure, without (dot) or with (triangle) previous LPS stimulation.

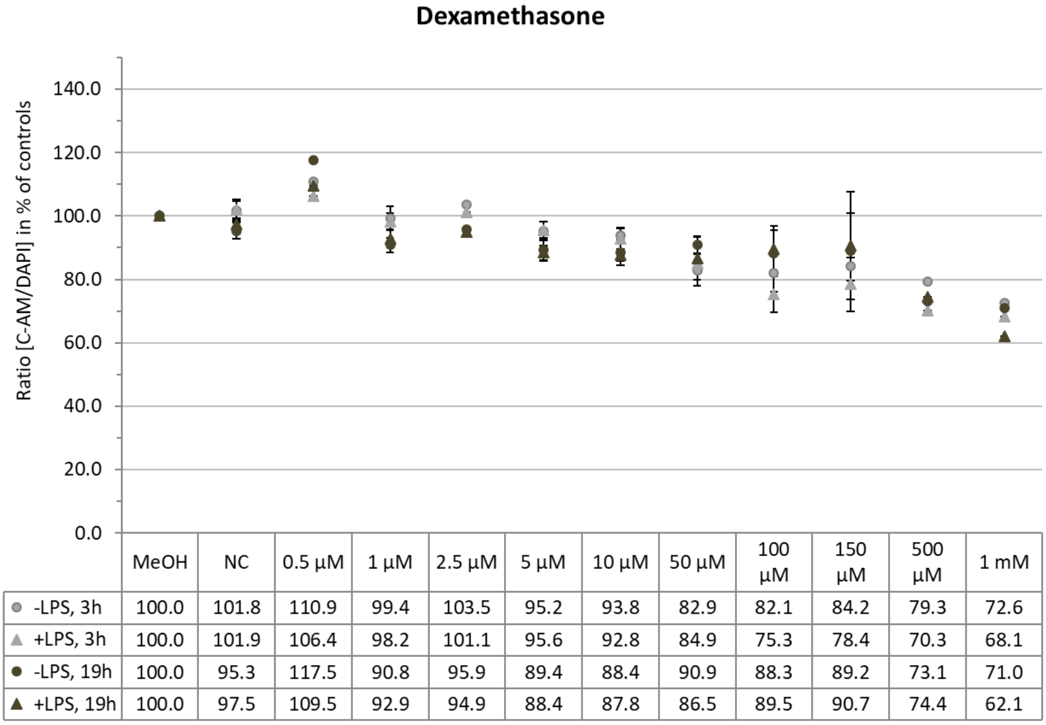

	MeOH	NC	0.5 µM	1 µM	2.5 µM	5 µM	10 µM	50 µM	100 µM	150 µM	500 µM	1 mM
-LPS, 3h	100.0	101.8	110.9	99.4	103.5	95.2	93.8	82.9	82.1	84.2	79.3	72.6
+LPS, 3h	100.0	101.9	106.4	98.2	101.1	95.6	92.8	84.9	75.3	78.4	70.3	68.1
-LPS, 19h	100.0	95.3	117.5	90.8	95.9	89.4	88.4	90.9	88.3	89.2	73.1	71.0
+LPS, 19h	100.0	97.5	109.5	92.9	94.9	88.4	87.8	86.5	89.5	90.7	74.4	62.1

Fig. S12 Cytotoxicity assays to determine cell viability for different concentrations of Dexamethasone. Concentrations were tested up to solubility. Viability in percentage of control for short- (3 h, gray) and long-term (19 h, black) exposure, without (dot) or with (triangle) previous LPS stimulation.

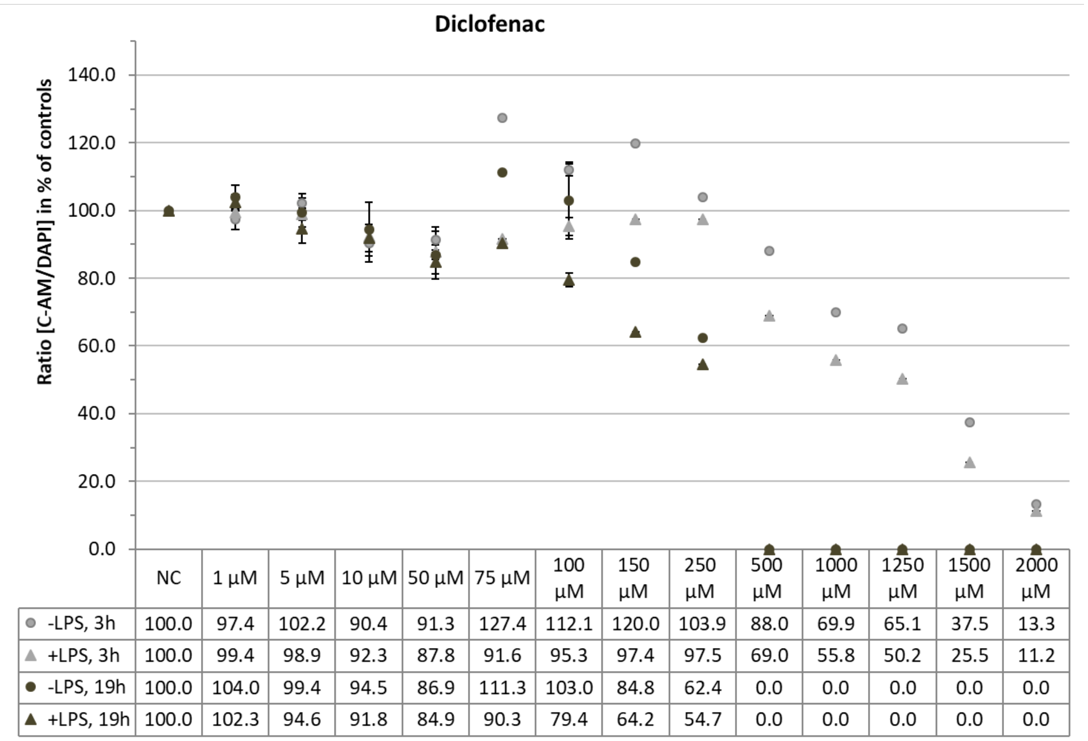

	NC	1 µM	5 µM	10 µM	50 µM	75 µM	100 µM	150 µM	250 µM	500 µM	1000 µM	1250 µM	1500 µM	2000 µM
-LPS, 3h	100.0	97.4	102.2	90.4	91.3	127.4	112.1	120.0	103.9	88.0	69.9	65.1	37.5	13.3
+LPS, 3h	100.0	99.4	98.9	92.3	87.8	91.6	95.3	97.4	97.5	69.0	55.8	50.2	25.5	11.2
-LPS, 19h	100.0	104.0	99.4	94.5	86.9	111.3	103.0	84.8	62.4	0.0	0.0	0.0	0.0	0.0
+LPS, 19h	100.0	102.3	94.6	91.8	84.9	90.3	79.4	64.2	54.7	0.0	0.0	0.0	0.0	0.0

Fig. S13 Cytotoxicity assays to determine cell viability for different concentrations of Diclofenac. Viability in percentage of control for short- (3 h, gray) and long-term (19 h, black) exposure, without (dot) or with (triangle) previous LPS stimulation.

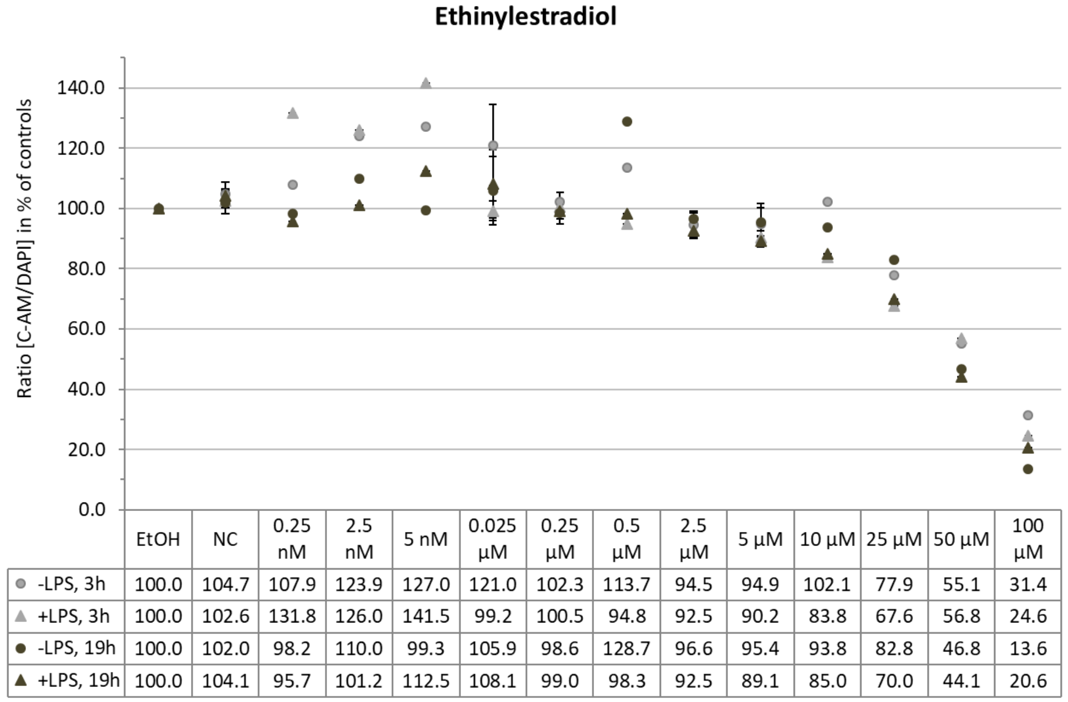

	EtOH	NC	0.25 nM	2.5 nM	5 nM	0.025 µM	0.25 µM	0.5 µM	2.5 µM	5 µM	10 µM	25 µM	50 µM	100 µM
-LPS, 3h	100.0	104.7	107.9	123.9	127.0	121.0	102.3	113.7	94.5	94.9	102.1	77.9	55.1	31.4
+LPS, 3h	100.0	102.6	131.8	126.0	141.5	99.2	100.5	94.8	92.5	90.2	83.8	67.6	56.8	24.6
-LPS, 19h	100.0	102.0	98.2	110.0	99.3	105.9	98.6	128.7	96.6	95.4	93.8	82.8	46.8	13.6
+LPS, 19h	100.0	104.1	95.7	101.2	112.5	108.1	99.0	98.3	92.5	89.1	85.0	70.0	44.1	20.6

Fig. S14 Cytotoxicity assays to determine cell viability for different concentrations of Ethinylestradiol. Viability in percentage of control for short- (3 h, gray) and long-term (19 h, black) exposure, without (dot) or with (triangle) previous LPS stimulation.

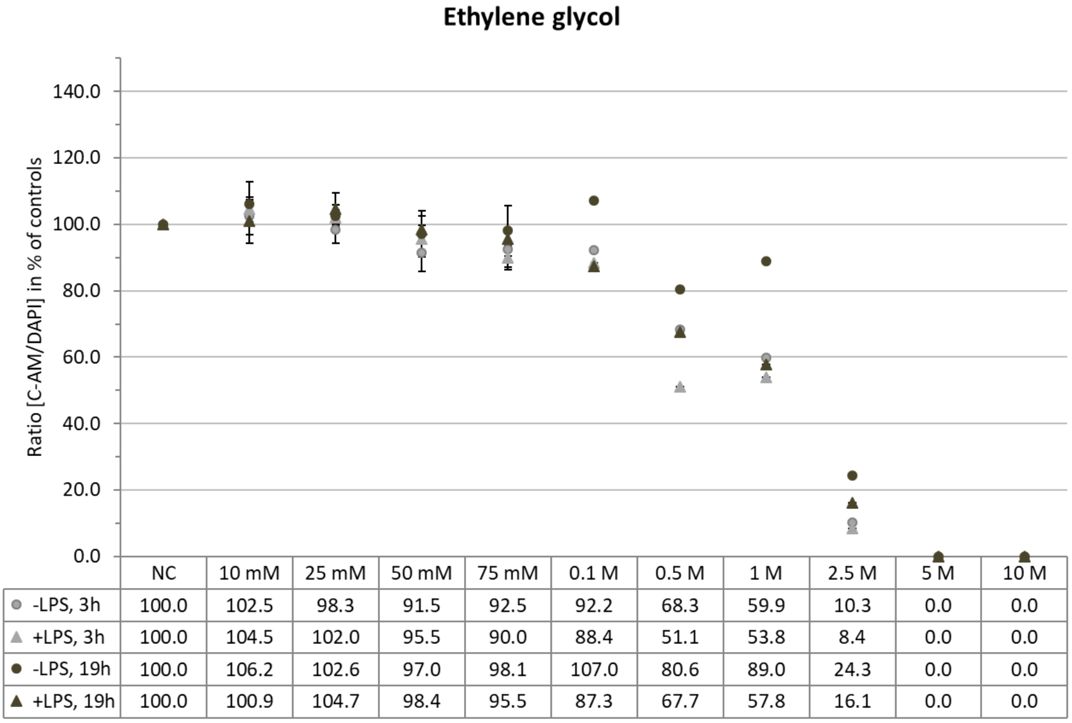

	NC	10 mM	25 mM	50 mM	75 mM	0.1 M	0.5 M	1 M	2.5 M	5 M	10 M
-LPS, 3h	100.0	102.5	98.3	91.5	92.5	92.2	68.3	59.9	10.3	0.0	0.0
+LPS, 3h	100.0	104.5	102.0	95.5	90.0	88.4	51.1	53.8	8.4	0.0	0.0
-LPS, 19h	100.0	106.2	102.6	97.0	98.1	107.0	80.6	89.0	24.3	0.0	0.0
+LPS, 19h	100.0	100.9	104.7	98.4	95.5	87.3	67.7	57.8	16.1	0.0	0.0

Fig. S15 Cytotoxicity assays to determine cell viability for different concentrations of Ethylene glycol. Viability in percentage of control for short- (3 h, gray) and long-term (19 h, black) exposure, without (dot) or with (triangle) previous LPS stimulation.

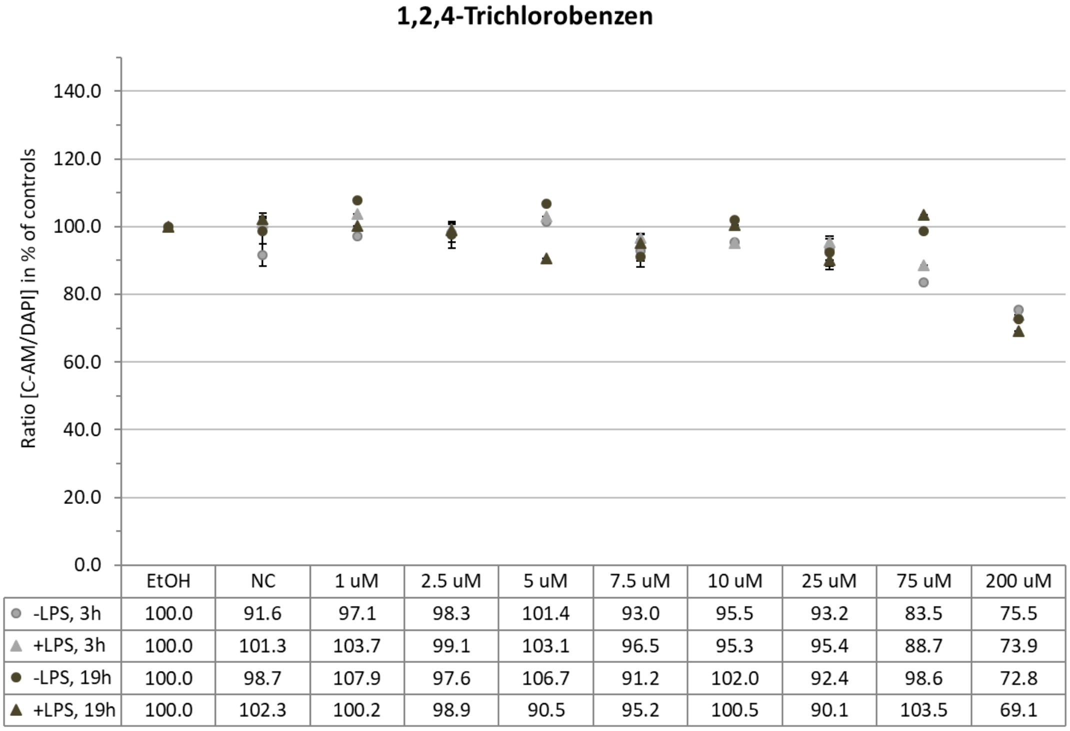

	EtOH	NC	1 uM	2.5 uM	5 uM	7.5 uM	10 uM	25 uM	75 uM	200 uM
-LPS, 3h	100.0	91.6	97.1	98.3	101.4	93.0	95.5	93.2	83.5	75.5
+LPS, 3h	100.0	101.3	103.7	99.1	103.1	96.5	95.3	95.4	88.7	73.9
-LPS, 19h	100.0	98.7	107.9	97.6	106.7	91.2	102.0	92.4	98.6	72.8
+LPS, 19h	100.0	102.3	100.2	98.9	90.5	95.2	100.5	90.1	103.5	69.1

Fig. S16 Cytotoxicity assays to determine cell viability for different concentrations of Trichlorobenzene. Concentrations were tested up to solubility. Viability in percentage of control for short- (3 h, gray) and long-term (19 h, black) exposure, without (dot) or with (triangle) previous LPS stimulation.

S7: p-values

p−values
- [0,0.001]
- (0.001,0.01]
- (0.01,0.05]
- (0.05,1]

01 = 03h_stimulated vs unstimulated control
02 = 19h_stimulated vs unstimulated control
03 = control short-vs long-term exposure
04= stimulated control short-vs long-term exposure
05.1= 03h_compound vs control_conc.1
05.2= 03h_compound vs control_conc.2
05.3= 03h_compound vs control_conc.3
05.4= 03h_compound vs control_conc.4

06.1= 03h_compound vs control (stimulated)_conc.1
06.2= 03h_compound vs control (stimulated)_conc.2
06.3= 03h_compound vs control (stimulated)_conc.3
06.4= 03h_compound vs control (stimulated)_conc.4
07.1= 19h_compound vs control_conc.1
07.2= 19h_compound vs control_conc.2
07.3= 19h_compound vs control_conc.3
07.4= 19h_compound vs control_conc.4

08.1= 19h_compound vs control (stimulated)_conc.1
08.2= 19h_compound vs control (stimulated)_conc.2
08.3= 19h_compound vs control (stimulated)_conc.3
08.4= 19h_compound vs control (stimulated)_conc.4
09 = overall differences due to LPS-stimulation at 3h
10 = overall differences due to LPS-stimulation at 19h
11 = overall time differences without stimulation
12 = overall time differences with stimulation

Phagocytosis

	01	02	03	04	05.1	05.2	05.3	05.4	06.1	06.2	06.3	06.4	07.1	07.2	07.3	07.4	08.1	08.2	08.3	08.4	09	10	11	12
Dex	0.215	0	0	0	0.21	0.021	0.204		0.049	0.037	0.016		0	0	0		0	0	0		0.213	0	0	0
DCF	0.701	0	0	0	0.529	0.692	0.512	0.974	0.638	0.489	0.222	0.789	0.696	0.845	0.88	0.083	0.817	0.921	0.679	0.151	0.292	0	0	0
BaP	0.479	0	0	0	0.659	0.268	0.143		0.62	0.366	0.145		0.275	0.238	0.053		0.252	0.328	0.054		0.293	0	0	0
BPA	0.349	0.023	0	0	0.767	0.929	0.556	0.929	0.97	0.922	0.457	0.266	0.541	0.498	0.362	0.196	0.345	0.388	0.026	0.021	0.61	0.005	0	0
EE2	0.77	0	0	0	0.108	0.047	0.002		0.043	0.225	0.003		0.559	0	0		0.868	0.002	0		0.493	0	0	0
TCB	0.721	0	0	0	0.604	0.563	0.256		0.574	0.773	0.421		0.891	0.972	0.43		0.926	0.799	0.647		0.753	0	0	0
EG	0.068	0	0	0	0.053	0.002	0.001	0	0.07	0.008	0.001	0.011	0.002	0.009	0	0	0.003	0.025	0	0.001	0.012	0	0	0
But	0.614	0.032	0	0	0.275	0.158	0.041	0.005	0.544	0.021	0.005	0.001	0.88	0.005	0.001	0	0.738	0	0	0	0.12	0.003	0	0

Fig. S17a p-values of each test chemical (y-axis, left) and treatment comparisons (x-axis) for the indicated immune parameter (y-axis, right)

Respiratory burst

	01	02	03	04	05.1	05.2	05.3	05.4	06.1	06.2	06.3	06.4	07.1	07.2	07.3	07.4	08.1	08.2	08.3	08.4	09	10	11	12
Dex	0.004	0	0	0	0.03	0	0		0	0	0		0	0	0		0	0	0		0.237	0	0	0
DCF	0.569	0.121	0.144	0.63	0.395	0.288	0.56	0.365	0.953	0.76	0.67	0.76	0.662	1	0.47	0.848	0.637	0.172	0.084	0.162	0.45	0.344	0	0.02
BaP	0.154	0.002	0.126	0.844	0.582	0	0		0.157	0	0		0.86	0.134	0.007		0.297	0.32	0.032		0.031	0	0	0.013
BPA	0.004	0.197	0.619	0.258	0.509	0.031	0.004	0.001	0.657	0.072	0.013	0.016	0.118	0.096	0	0	0.643	0.195	0	0	0	0.298	0.022	0.678
EE2	0.004	0.026	0	0	0.474	0.059	0.009		0.116	0.007	0		0.34	0.015	0.001		0.07	0.001	0		0.001	0.017	0	0
TCB	0.02	0	0.805	0.13	0.599	0.8	0.508		0.781	0.211	0.867		0.867	0.447	0.926		0.936	0.08	0.357		0	0	1	0.006
EG	0	0	0.002	0	0.807	0.6	0.053	0.079	0.804	0.246	0.018	0.011	0.882	0.848	0.394	0.048	0.726	0.391	0.174	0.005	0	0	0	0
But	0.02	0.042	0.726	0.523	0.694	0.266	0.124	0.128	0.11	0.014	0.02	0.001	0.955	0.321	0.01	0.008	0.249	0.019	0.001	0	0.043	0.126	0.211	0.081

***IL-1β* (ΔCt)**

	01	02	03	04	05.1	05.2	05.3	05.4	06.1	06.2	06.3	06.4	07.1	07.2	07.3	07.4	08.1	08.2	08.3	08.4	09	10	11	12
Dex	0	0.039	0.341	0.041	0.145				0.005				0				0				0	0.095	0.611	0
DCF	0	0.316	0.04	0.269	0.395				0.154				0.103				0.289				0	0.116	0.302	0.006
BaP	0	0.279	0.147	0	0.205				0.322				0.121				0.001				0	0.952	0.957	0
BPA	0	0	0.543	0	0.005				0.515				0				0.039				0	0	0.105	0
EE2	0.001	0.245	0.004	0.466	0.595				0.568				0.011				0.01				0	0.163	0.02	0.682
TCB	0	0.033	0	0	0.58				0.985				0.626				0.962				0	0.004	0	0
EG	0	0.435	0.061	0	0.015				0.099				0.681				0				0	0.112	0.582	0
But	0	0.873	0.016	0	0.947				0.3				0.005				0				0	0.384	0	0

Fig. S17b p-values of each test chemical (y-axis, left) and treatment comparisons (x-axis) for the indicated immune parameter (y-axis, right)

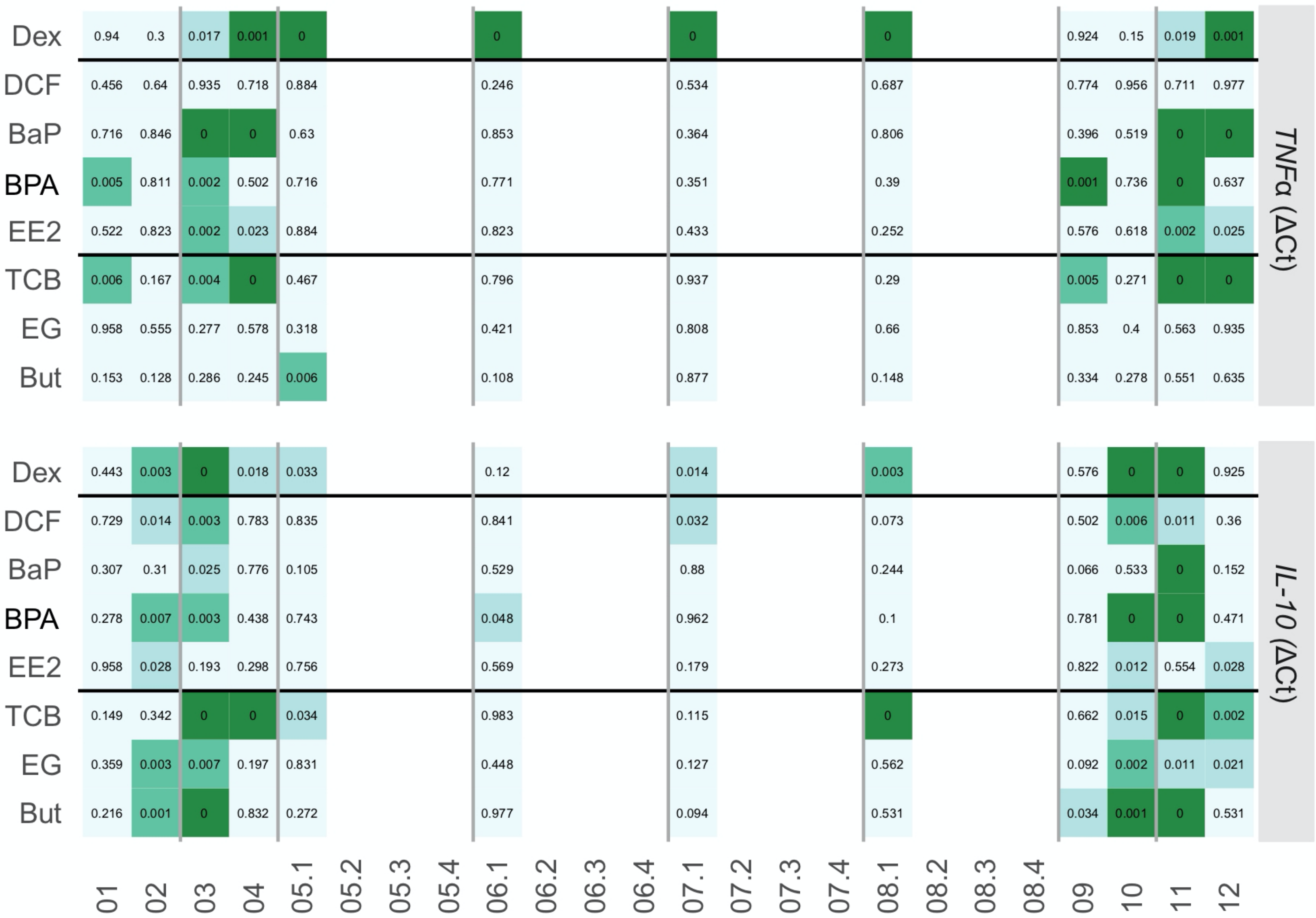

Fig. S17c p-values of each test chemical (y-axis, left) and treatment comparisons (x-axis) for the indicated immune parameter (y-axis, right)

S8: Test statistics

(test statistics = effect size normalized by standard error)

Test statistics: Immune parameters
- suppressed
- stimulated

01 = 03h_stimulated vs unstimulated control
02 = 19h_stimulated vs unstimulated control
03 = control short-vs long-term exposure
04= stimulated control short-vs long-term exposure
05.1= 03h_compound vs control_conc.1
05.2= 03h_compound vs control_conc.2
05.3= 03h_compound vs control_conc.3
05.4= 03h_compound vs control_conc4
06.1= 03h_compound vs control (stimulated)_conc.1
06.2= 03h_compound vs control (stimulated)_conc.2
06.3= 03h_compound vs control (stimulated)_conc.3
06.4= 03h_compound vs control (stimulated)_conc.4
07.1= 19h_compound vs control_conc.1
07.2= 19h_compound vs control_conc.2
07.3= 19h_compound vs control_conc.3
07.4= 19h_compound vs control_conc.4
08.1= 19h_compound vs control (stimulated)_conc.1
08.2= 19h_compound vs control (stimulated)_conc.2
08.3= 19h_compound vs control (stimulated)_conc.3
08.4= 19h_compound vs control (stimulated)_conc.4
09 = overall differences due to LPS-stimulation at 3h
10 = overall differences due to LPS-stimulation at 19h
11 = overall time differences without stimulation
12 = overall time differences with stimulation

Phagocytosis

	01	02	03	04	05.1	05.2	05.3	05.4	06.1	06.2	06.3	06.4	07.1	07.2	07.3	07.4	08.1	08.2	08.3	08.4	09	10	11	12
Dex	-1.2	-4.3	-11.7	-14.7	1.3	2.3	1.3		2	2.1	2.4		5.7	6.8	6.5		6.4	6.6	7.6		-1.2	-6.1	-12.3	-17.1
DCF	-0.4	-5.4	-13.1	-18.2	-0.6	-0.4	-0.7	0	-0.5	-0.7	-1.2	-0.3	-0.4	0.2	0.2	1.7	-0.2	-0.1	-0.4	1.4	-1.1	-9.8	-21.4	-30.2
BaP	0.7	-6.3	-13.4	-20.4	0.4	1.1	1.5		0.5	0.9	1.5		1.1	1.2	1.9		1.1	1	1.9		1.1	-10	-20.7	-31.7
BPA	-0.9	-2.3	-7.3	-8.6	-0.3	-0.1	-0.6	0.1	0	0.1	0.7	1.1	0.6	0.7	0.9	1.3	0.9	0.9	2.3	2.3	-0.5	-2.9	-10.9	-13.2
EE2	-0.3	-7.1	-26.3	-33.1	1.6	2	3.2		2	1.2	3.1		-0.6	3.9	7.9		-0.2	3.1	7.7		-0.7	-11.4	-39.7	-50.5
TCB	-0.4	-4.6	-11.1	-15.4	-0.5	-0.6	-1.1		-0.6	-0.3	-0.8		0.1	0	-0.8		0.1	0.3	-0.5		-0.3	-7.1	-16.9	-23.7
EG	-1.8	-8.1	-11.9	-18.2	-1.9	-3.1	-3.4	-3.8	-1.8	-2.7	-3.4	-2.5	-3.2	-2.6	-4.2	-4.5	-3	-2.2	-4.3	-3.2	-2.5	-13.4	-21.6	-32.5
But	0.5	-2.2	-8.9	-11.6	1.1	1.4	2.1	2.9	0.6	2.3	2.8	3.5	0.2	2.8	3.3	4.1	-0.3	3.7	4	4.7	1.6	-3	-14.4	-19

Fig. S18a Test statistics of each test chemical (y-axis, left) and treatment comparisons (x-axis) for the indicated immune parameter (y-axis, right)

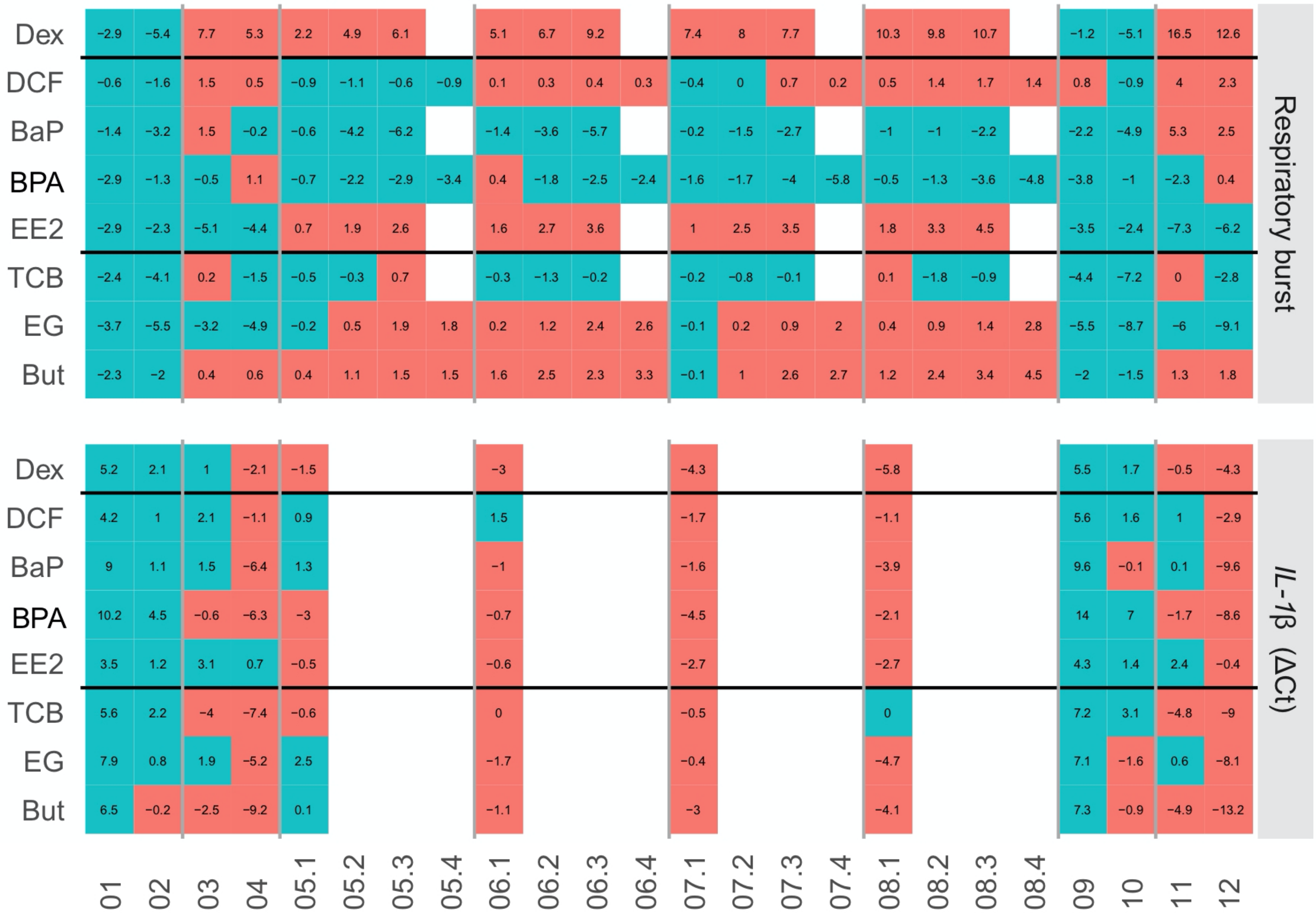

Fig. S18b Test statistics of each test chemical (y-axis, left) and treatment comparisons (x-axis) for the indicated immune parameter (y-axis, right)

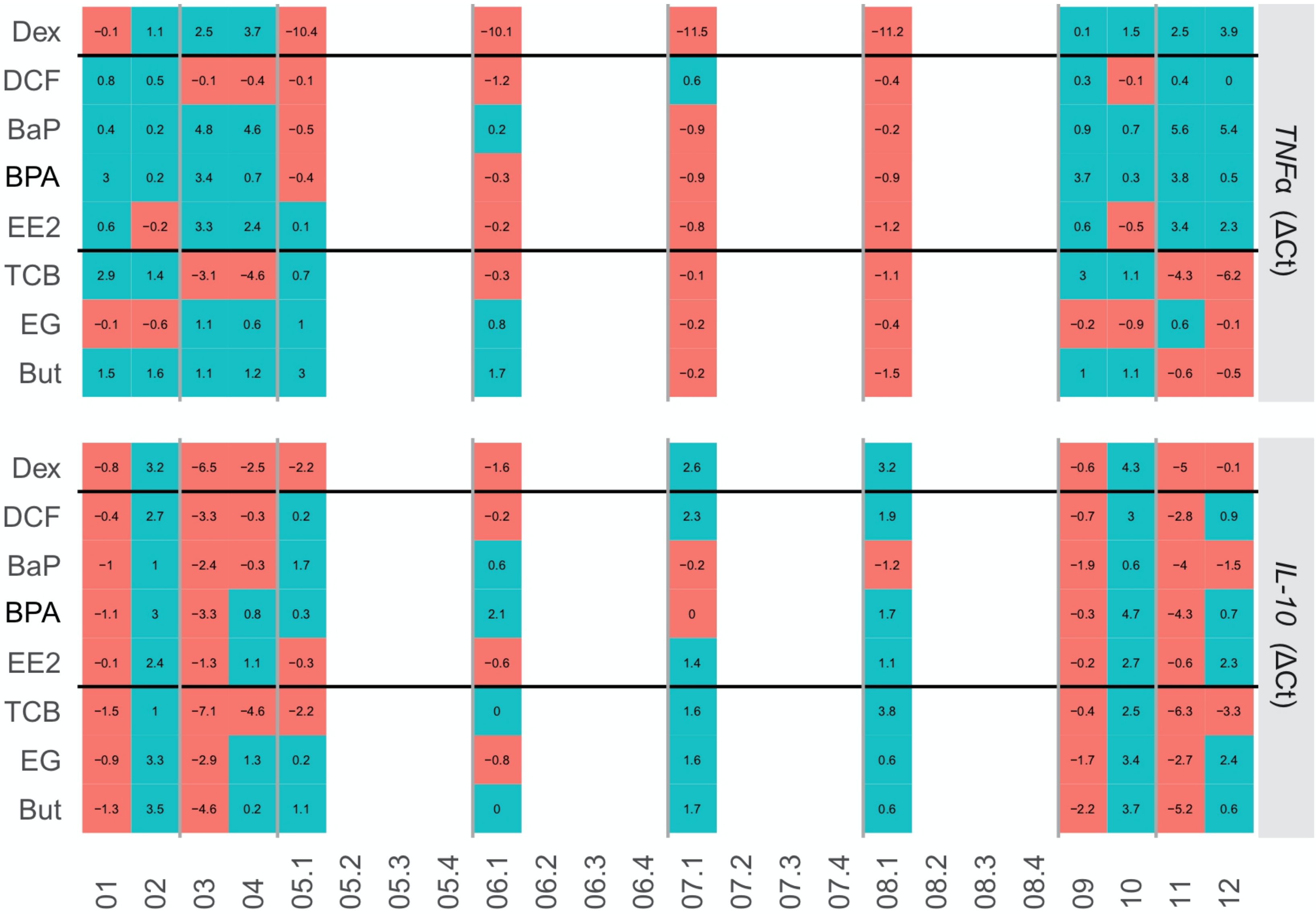

Fig. S18c Test statistics of each test chemical (y-axis, left) and treatment comparisons (x-axis) for the indicated immune parameter (y-axis, right)

S9: Approaches for the data analysis / statistic

Modifications of the data analysis were carried out to test for analysis-introduced bias:

- The raw data of each measurement was calculated in percent of the control (control was set as 100%) to account for natural variance. For this trial, the mean of the three technical replicates was used. The reaction pattern was similar to the results obtained with R (described in the main text).
- Model equation with all interactions was tried, but did not change the overall findings
- The effect size was expressed in percentage for each compound and endpoint. No differences between the two categories (immunomodulating and non-immunomodulating compounds) were seen.
- An overview was done whether observed effects are more / less significant depending on LPS stimulation. There was a trend for stronger effects with previous LPS stimulation, but for both compound categories – hence, overall findings were the same.
- Clustering of the compound categories by the observed reaction pattern (up / down regulation) failed
- The step “select valid data” was skipped for the immune genes. Those data was included if an LPS stimulation was detected at least for the phagocytosis or the NBT assay at any time point. This changed the results for p-values, test statistics and boxplots just minor.
- Only significant compound effects were compered if additionally the corresponding LPS stimulation was significant, too. The results (significant effect pattern and underlying reaction pattern) were similar and did not change the overall findings. This was done for both “select valid data” approaches.

The results showed that the findings which are shown in the main text were not influenced by an analysis-introduced bias

S10: Boxplots for each test compound

Test chemicals in alphabetic order

Benzo(a)pyrene

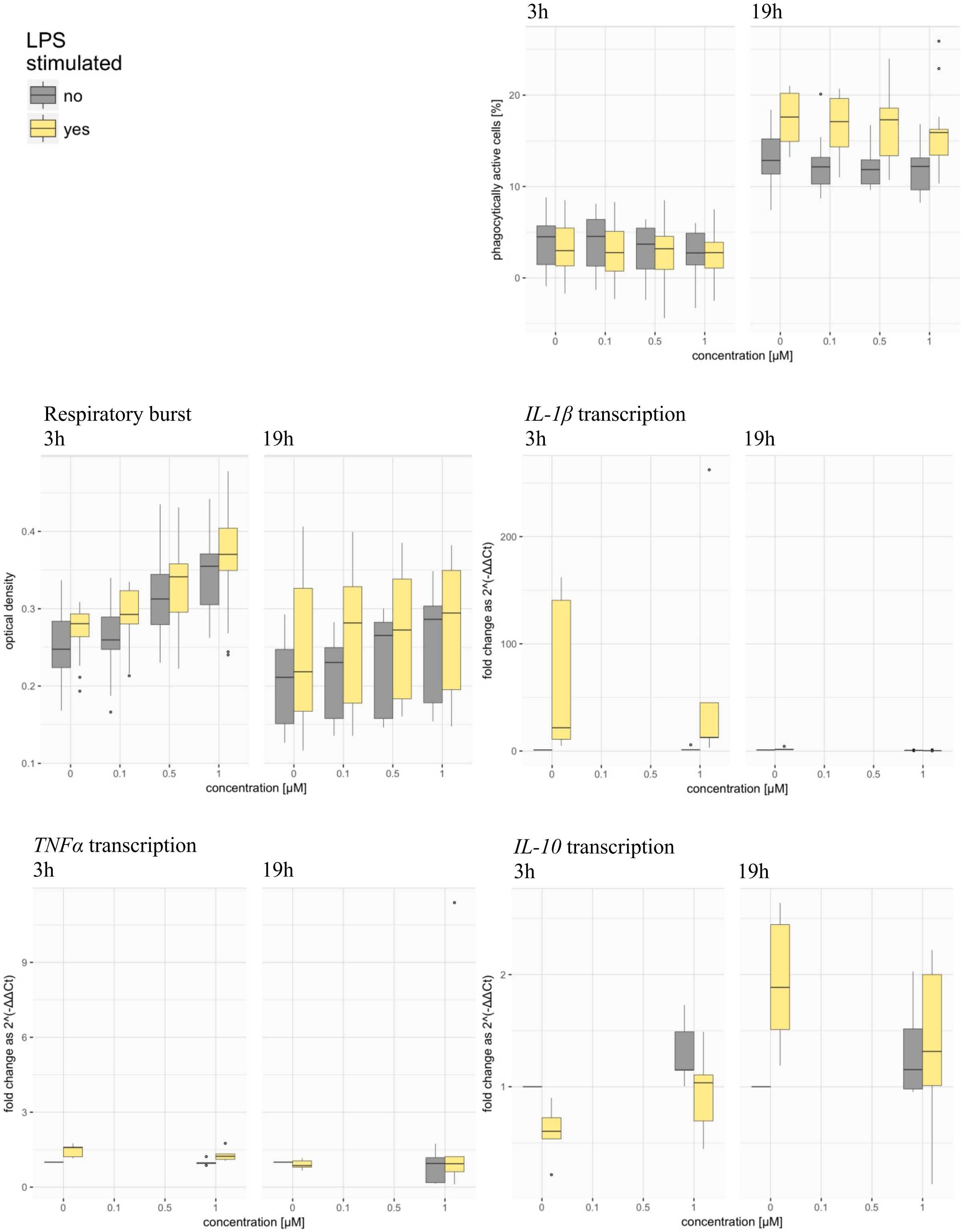

Fig. S19 Boxplots for each immune parameter after short- (3 h) and long-term (19 h) exposure with Benzo(a)pyrene – without (gray) or with (yellow) previous LPS stimulation

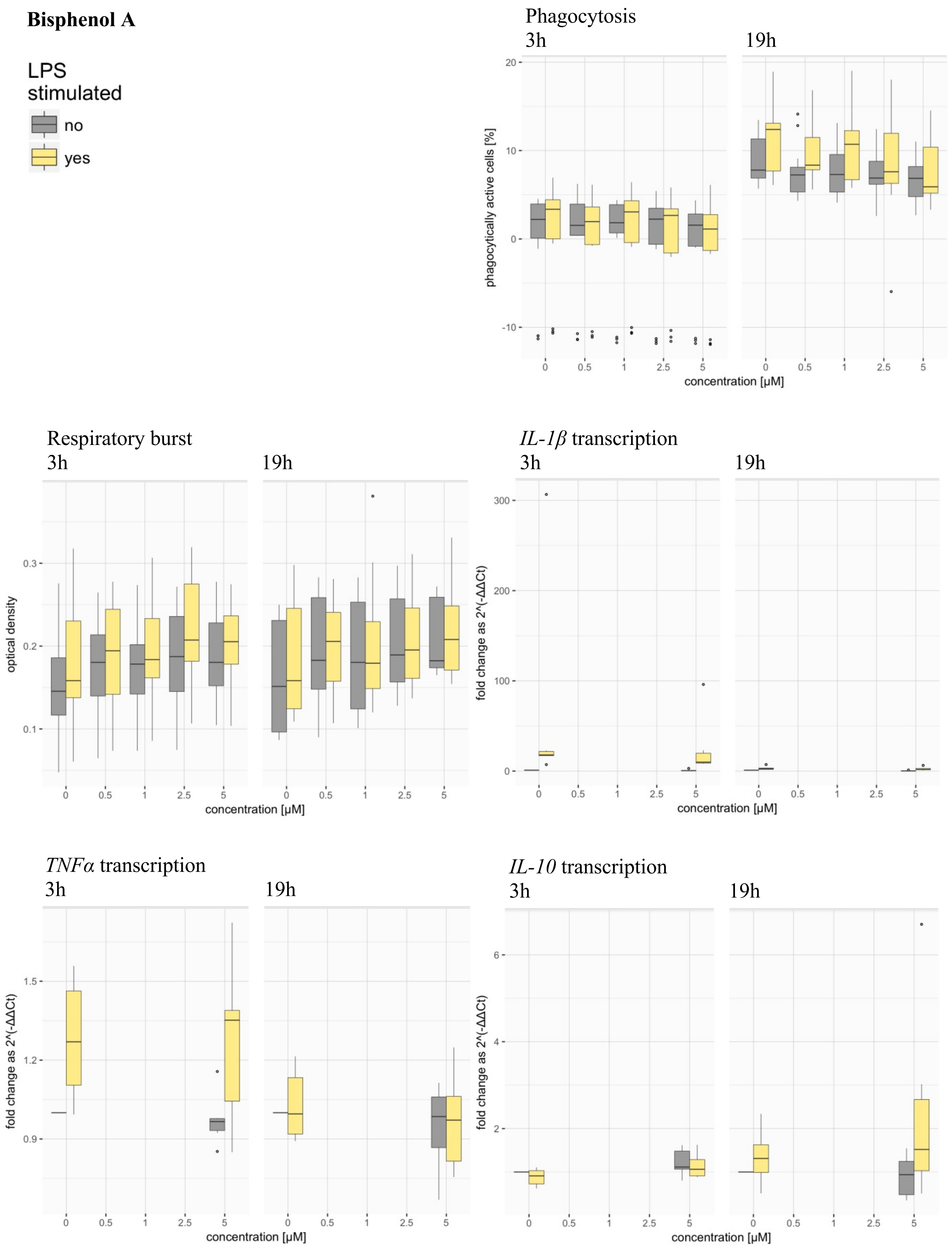

Fig. S20 Boxplots for each immune parameter after short- (3 h) and long-term (19 h) exposure with Bisphenol A – without (gray) or with (yellow) previous LPS stimulation

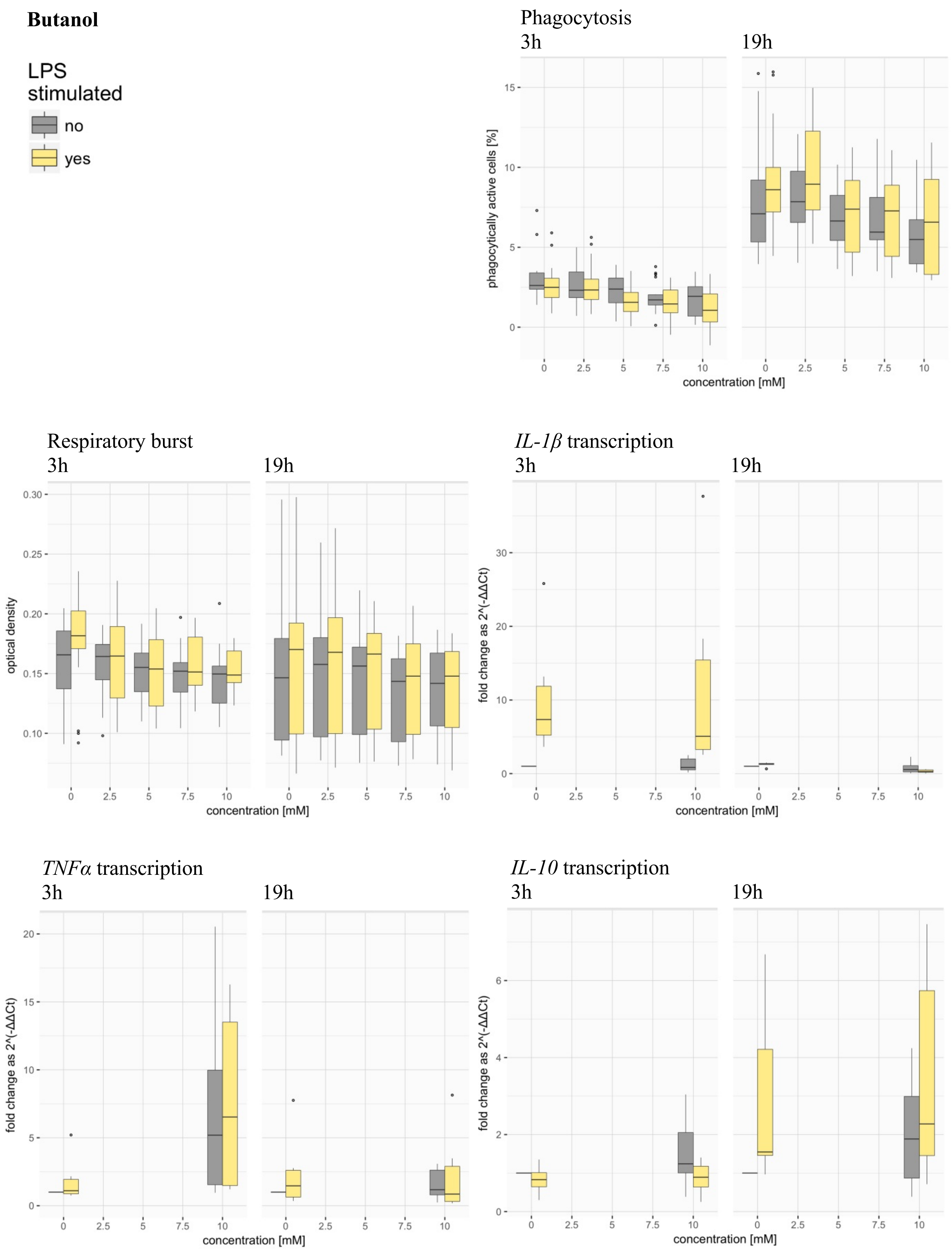

Fig. S21 Boxplots for each immune parameter after short- (3 h) and long-term (19 h) exposure with Butanol – without (gray) or with (yellow) previous LPS stimulation

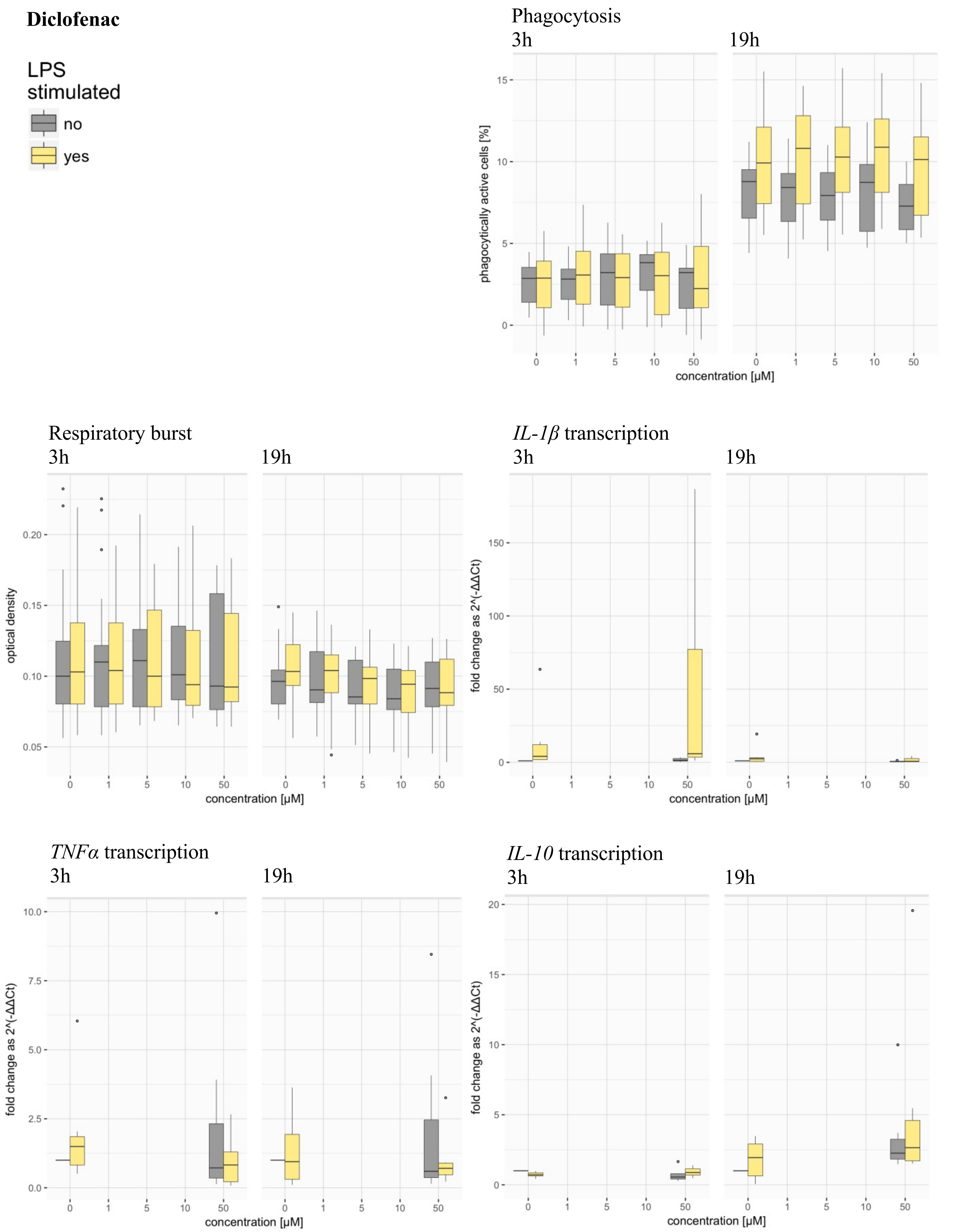

Fig. S22 Boxplots for each immune parameter after short- (3 h) and long-term (19 h) exposure with Diclofenac – without (gray) or with (yellow) previous LPS stimulation

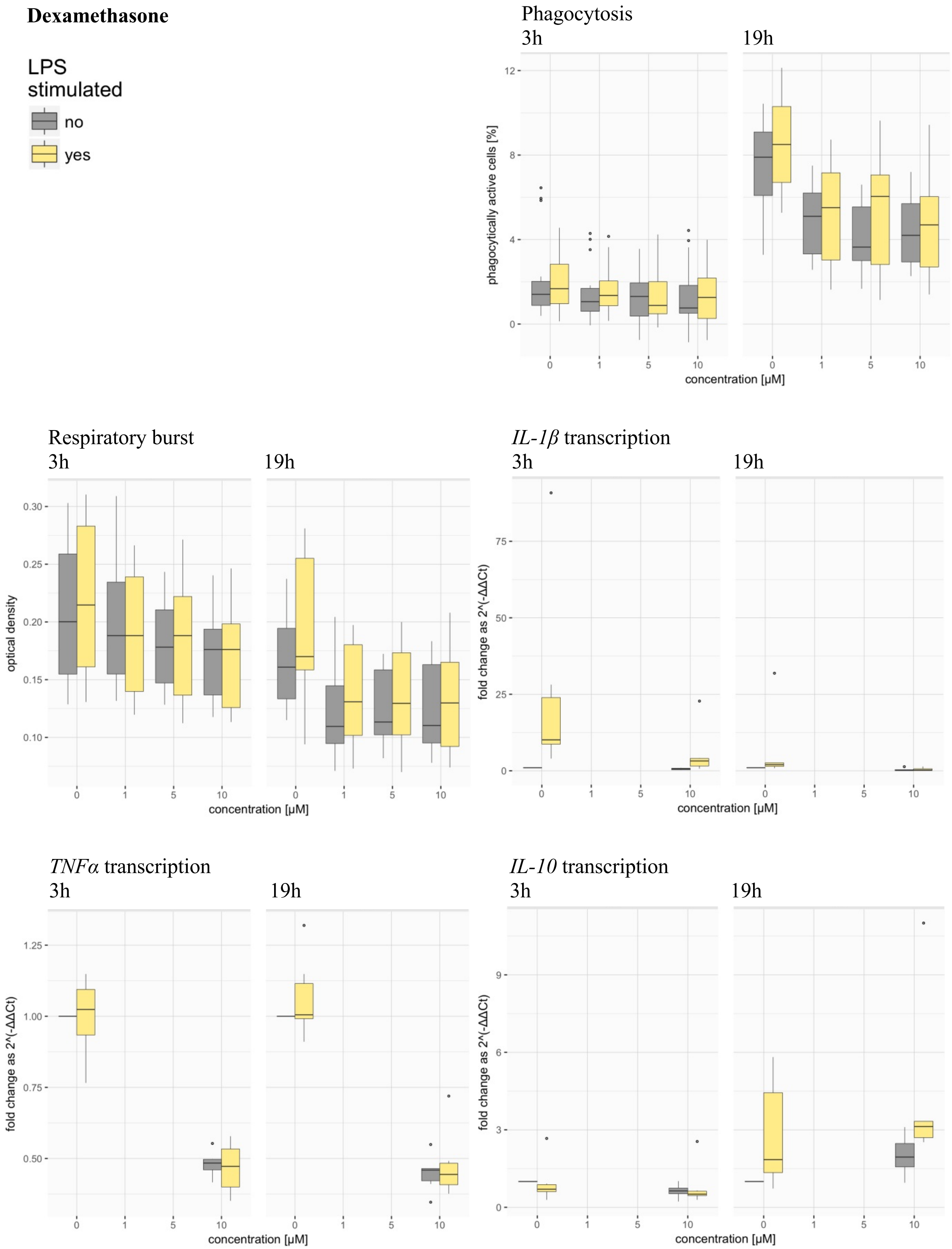

Fig. S23 Boxplots for each immune parameter after short- (3 h) and long-term (19 h) exposure with Dexamethasone – without (gray) or with (yellow) previous LPS stimulation

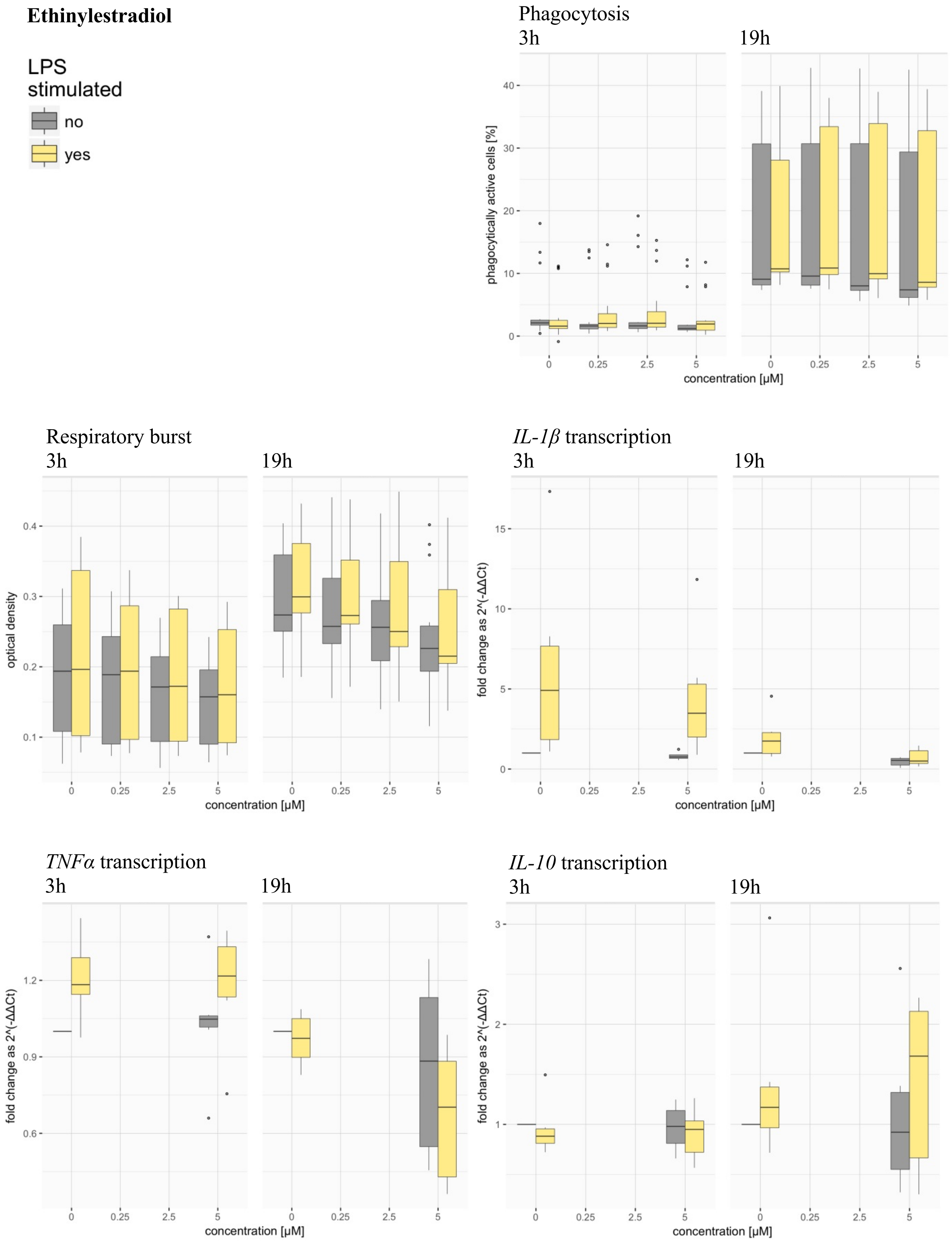

Fig. S24 Boxplots for each immune parameter after short- (3 h) and long-term (19 h) exposure with Ethinylestradiol – without (gray) or with (yellow) previous LPS stimulation

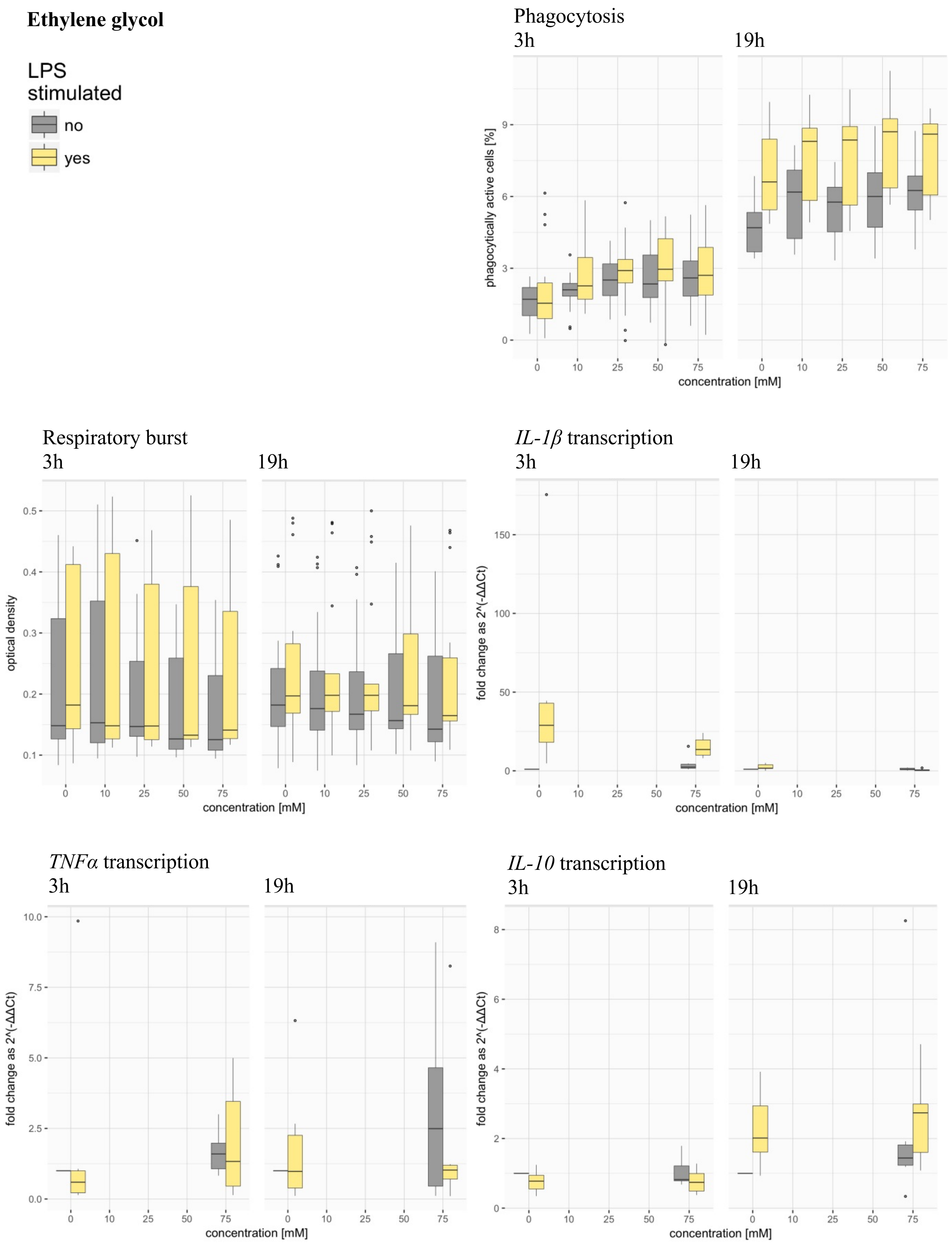

Fig. S25 Boxplots for each immune parameter after short- (3 h) and long-term (19 h) exposure with Ethylene glycol – without (gray) or with (yellow) previous LPS stimulation

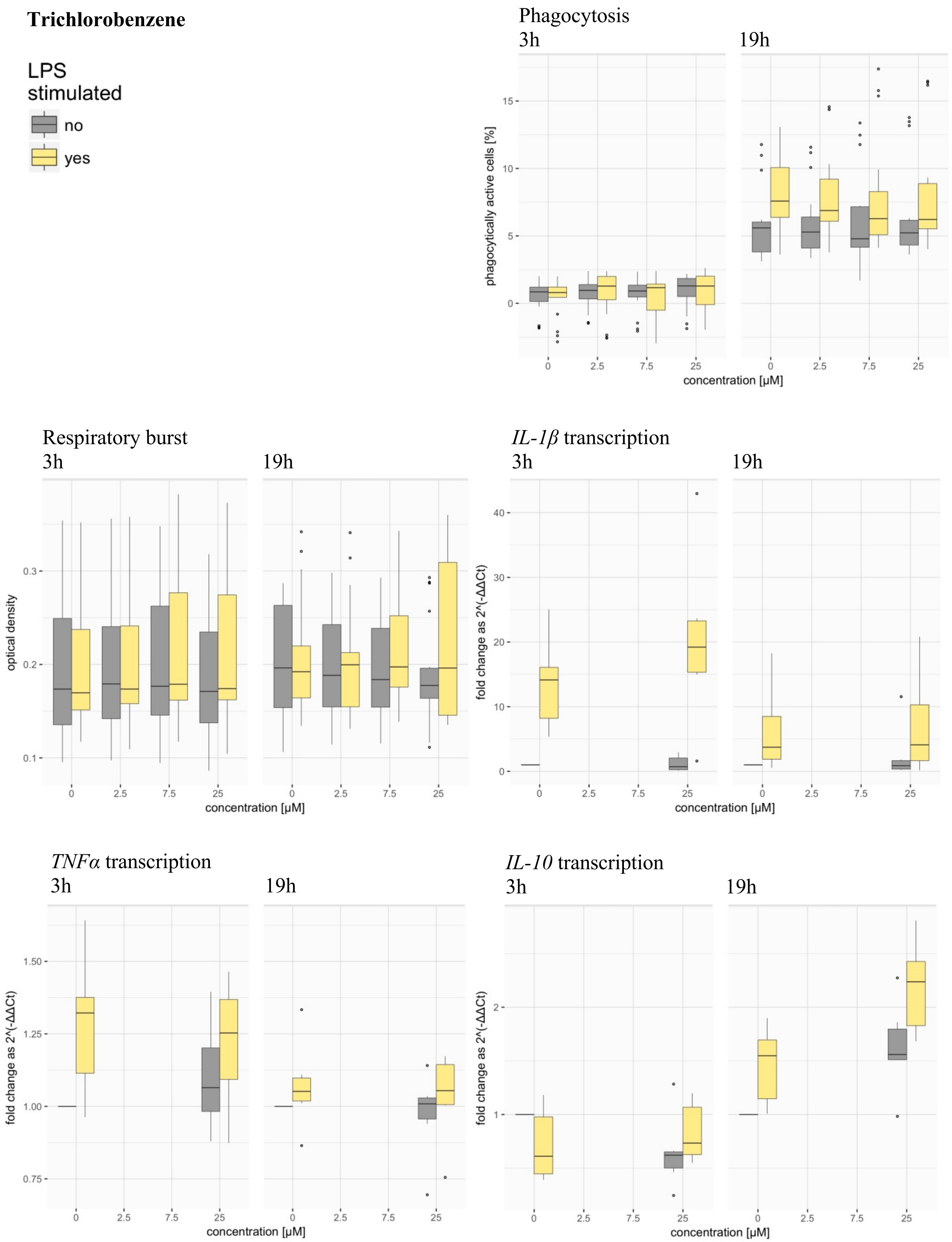

Fig. S26 Boxplots for each immune parameter after short- (3 h) and long-term (19 h) exposure with Trichlorobenzene – without (gray) or with (yellow) previous LPS stimulation

S11: Assay response patterns

When analyzing the response patterns of the assay immune parameters to the two groups of test chemicals, immunotoxicants and baseline toxicants, in more detail: (I) Did the immunotoxicants cause significant effects while the non-immunotoxicants induce non-significant effects? (II) Did they differ in effect size (normalized by standard error, supplement S8)? This was not the case. Both chemical groups resulted in significant as well as non-significant responses of the immune parameters and there was no distinct pattern for the effect size. (III) Did the immunotoxicants cause effects that are different to those induced by the baseline toxicants? For instance, did immunotoxicants always inhibit phagocytosis while the non-immunotoxicants always activate it? This did not apply. (IV) Did the presence of LPS modify the assay response differently of immunotoxicants and baseline toxicants? This was not the case; there was no consistently different influence of the LPS stimulation on the immune parameter responses to immunotoxicants and non-immunotoxicants.

S12: Heat map

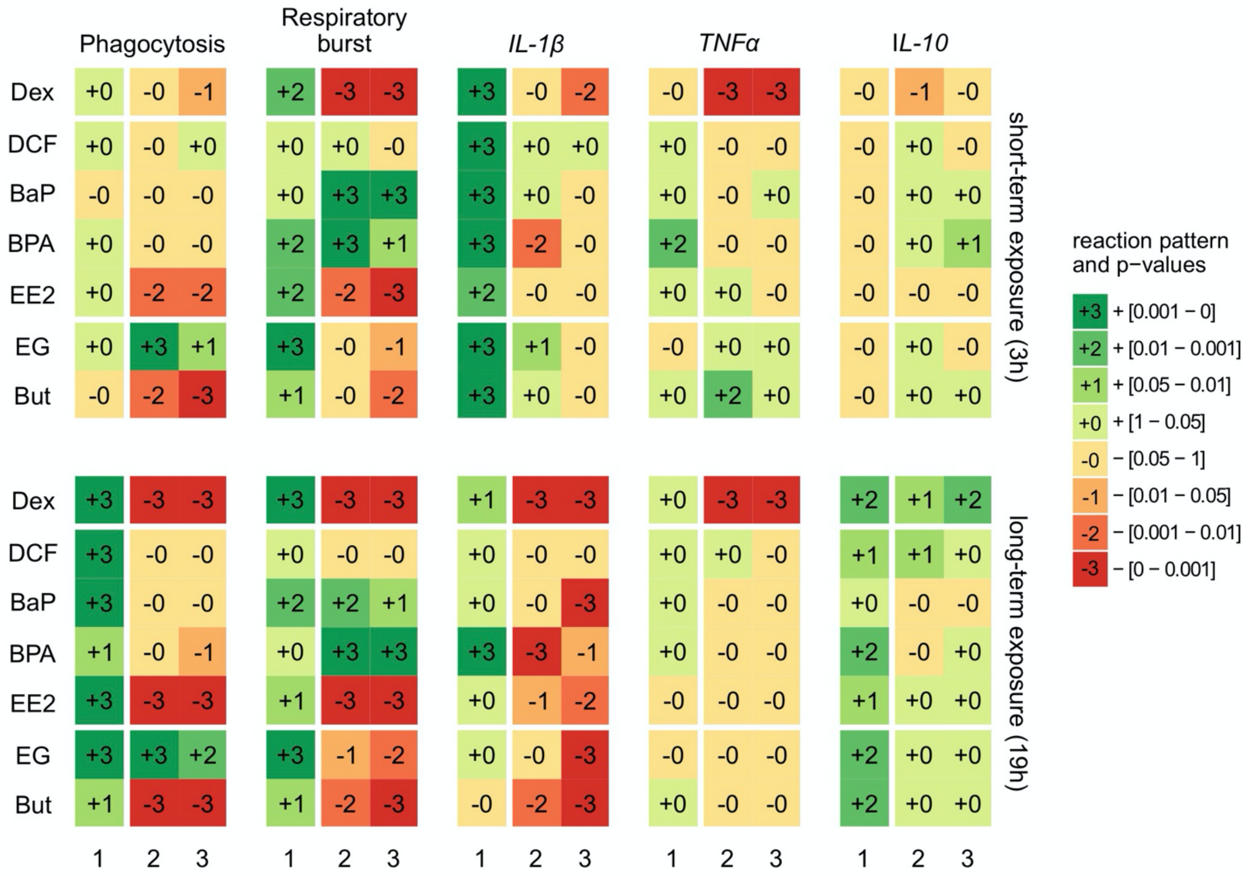

Fig. S27 Overview of the in vitro immuno-assay results. Depictured are the results for the comparison of the LPS-stimulated control to the non-stimulated control (column 1 of each immune parameter) and the results for the comparison of the highest applied chemical concentration without (column 2) and with LPS stimulation (column 3) compared to the control without or with LPS stimulation, respectively. Data is displayed for short- (3 h, top) and long-term (19 h, bottom) chemical exposure separately. Colors represent the p-values of the comparisons with underlying decreasing effects in red and for increasing effects in green; the darker the color the higher the statistical significance in relation to the corresponding control. For the transcription of cytokines, the statistical analysis was based on ΔCt values. The reference compound dexamethasone (Dex), the other immunotoxic chemicals (diclofenac: DCF, benzo(a)pyrene: BaP, bisphenol A: BPA and ethinylestradiol: EE2) and the chemicals with no reported immunotoxic action (ethylene glycol: EG and butanol: But) were optically clustered with horizontal, white dividing lines
